# Supplementary figures and images for: 3D-Analysis of a non-planispiral ammonoid from the Hunsrück Slate: natural or pathological variation? (part 2 of 2)
Source: PeerJ. 2017 Jun 30;5:e3526. doi: 10.7717/peerj.3526 (PMC5494166; doi:10.7717/peerj.3526)

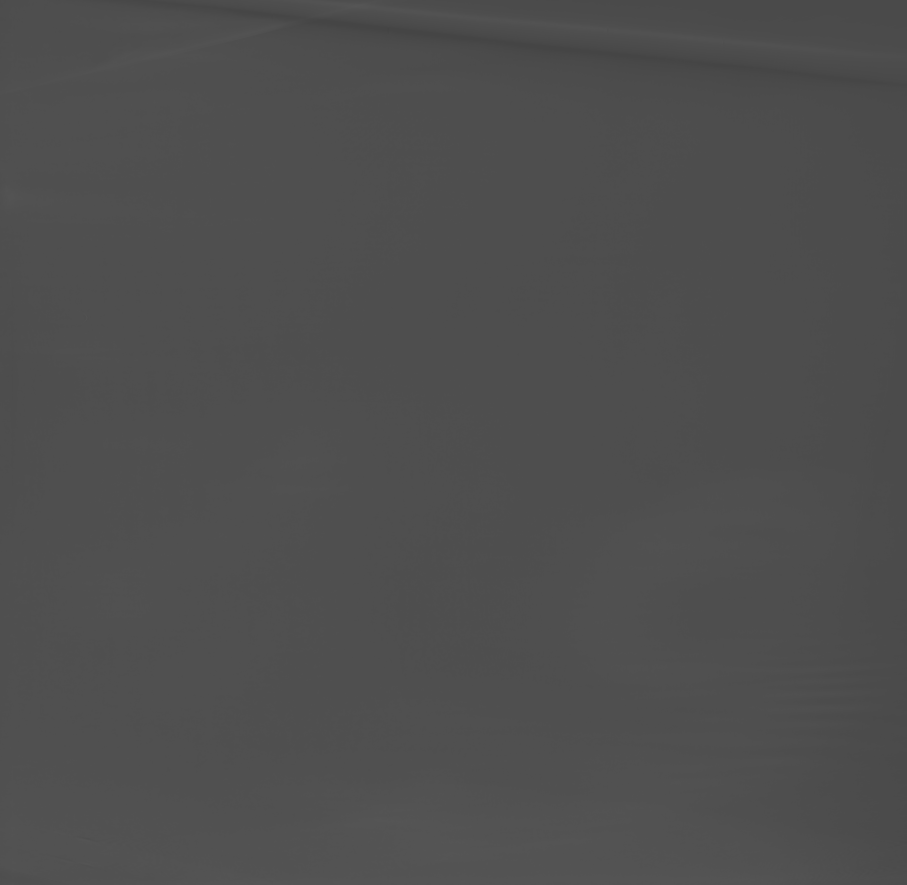

Supplement: Supplemental Information 2 — The used imagestack in the x-z-plane (bmp format) as it was obtained and used in SPIERS. [file peerj-05-3526-s002.zip › front_x-z-plane_100.bmp]

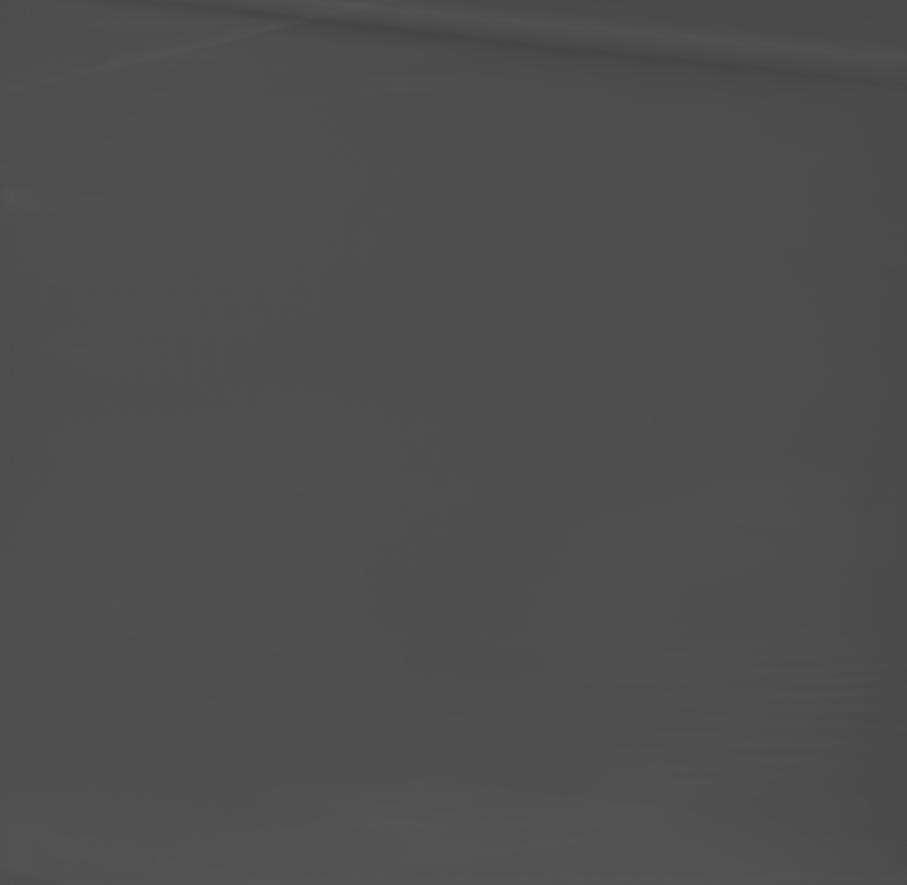

Supplement: Supplemental Information 2 — The used imagestack in the x-z-plane (bmp format) as it was obtained and used in SPIERS. [file peerj-05-3526-s002.zip › front_x-z-plane_101.bmp]

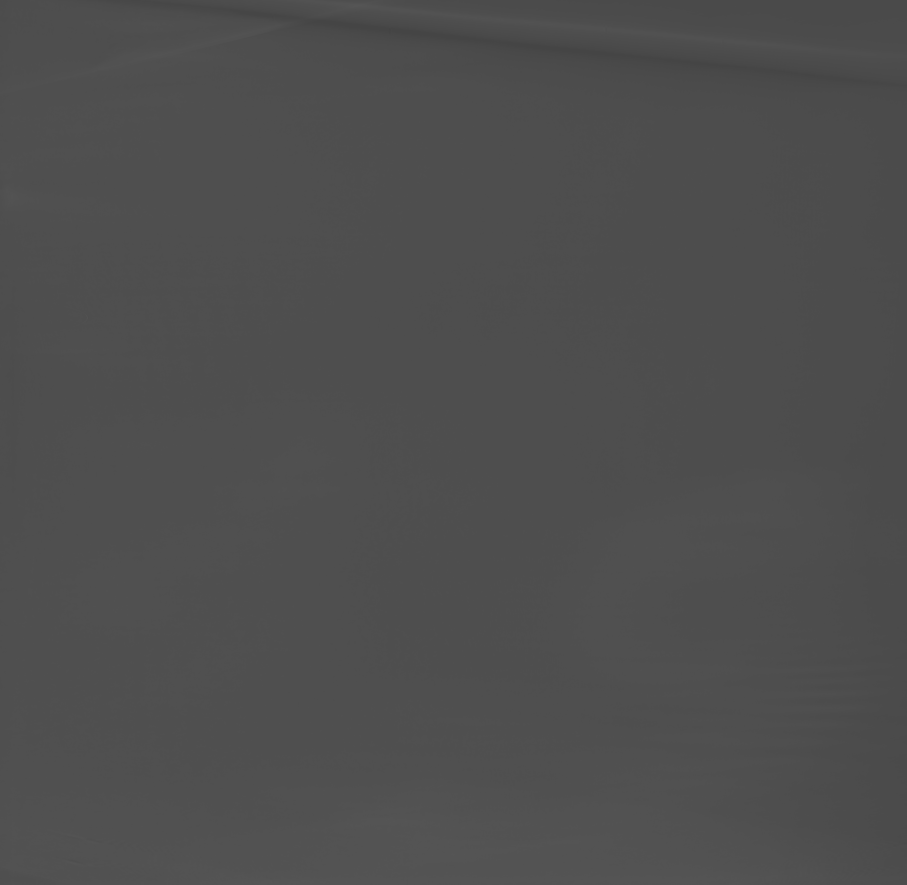

Supplement: Supplemental Information 2 — The used imagestack in the x-z-plane (bmp format) as it was obtained and used in SPIERS. [file peerj-05-3526-s002.zip › front_x-z-plane_102.bmp]

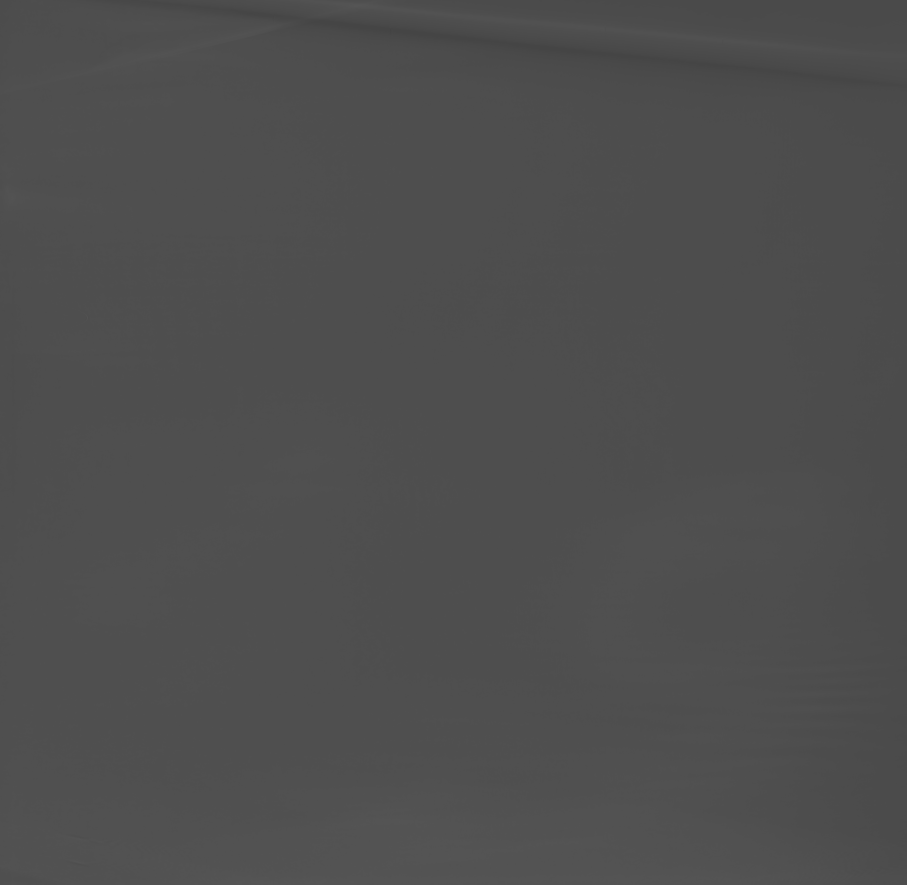

Supplement: Supplemental Information 2 — The used imagestack in the x-z-plane (bmp format) as it was obtained and used in SPIERS. [file peerj-05-3526-s002.zip › front_x-z-plane_103.bmp]

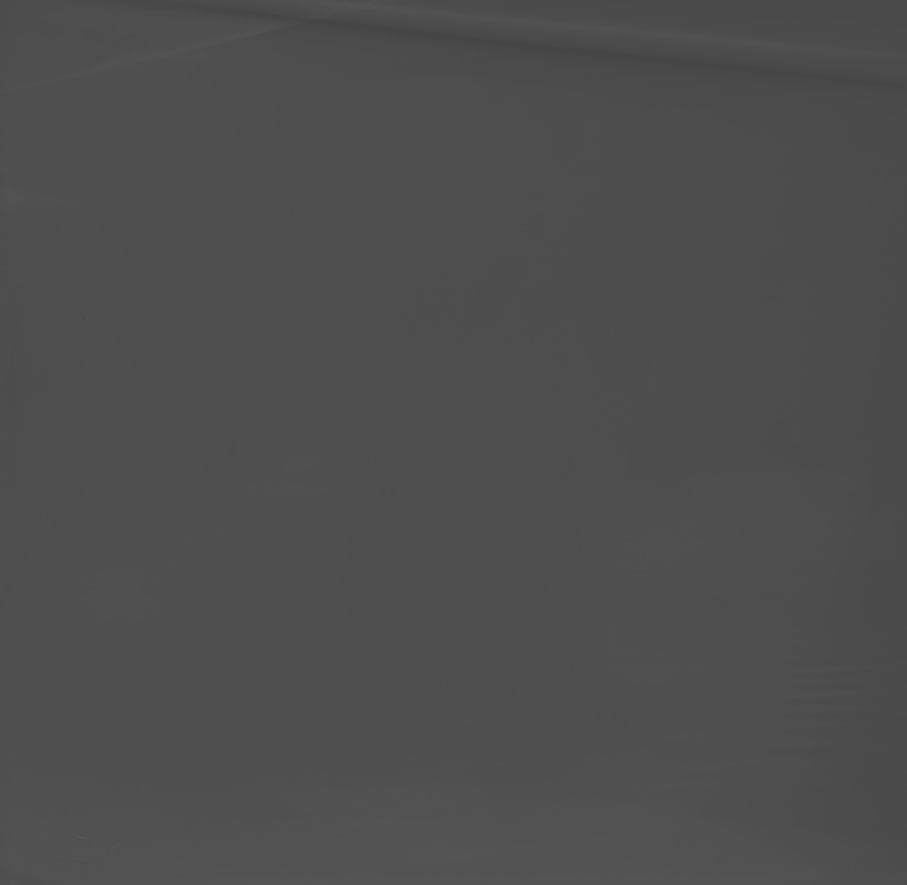

Supplement: Supplemental Information 2 — The used imagestack in the x-z-plane (bmp format) as it was obtained and used in SPIERS. [file peerj-05-3526-s002.zip › front_x-z-plane_104.bmp]

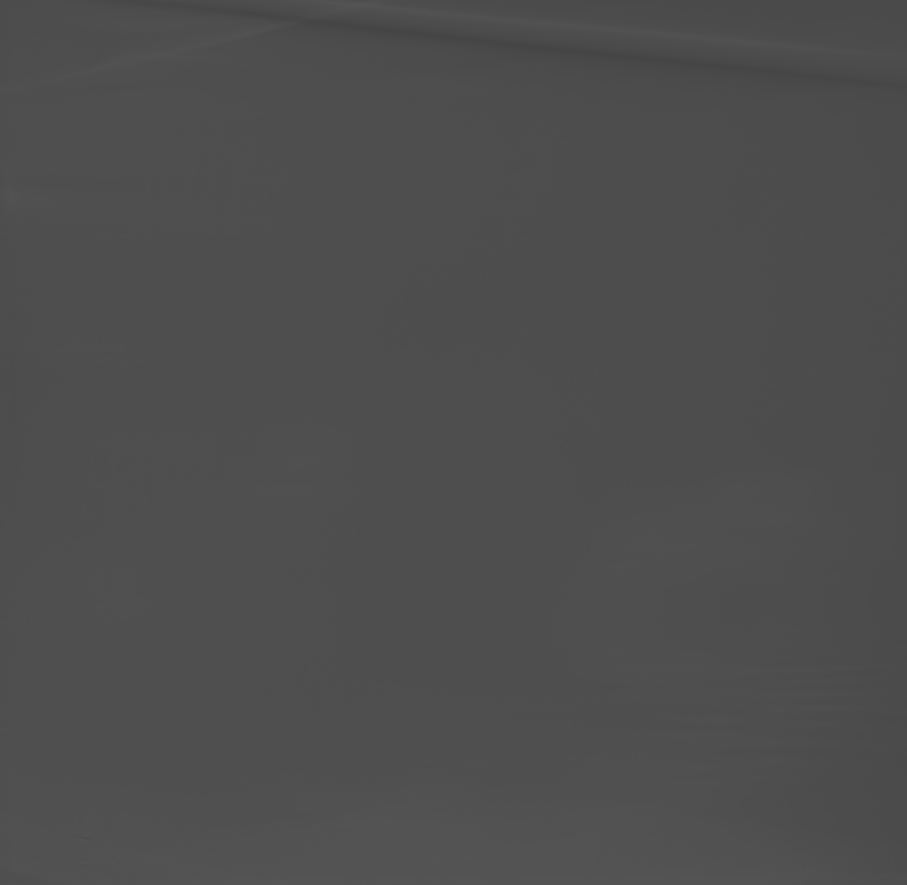

Supplement: Supplemental Information 2 — The used imagestack in the x-z-plane (bmp format) as it was obtained and used in SPIERS. [file peerj-05-3526-s002.zip › front_x-z-plane_105.bmp]

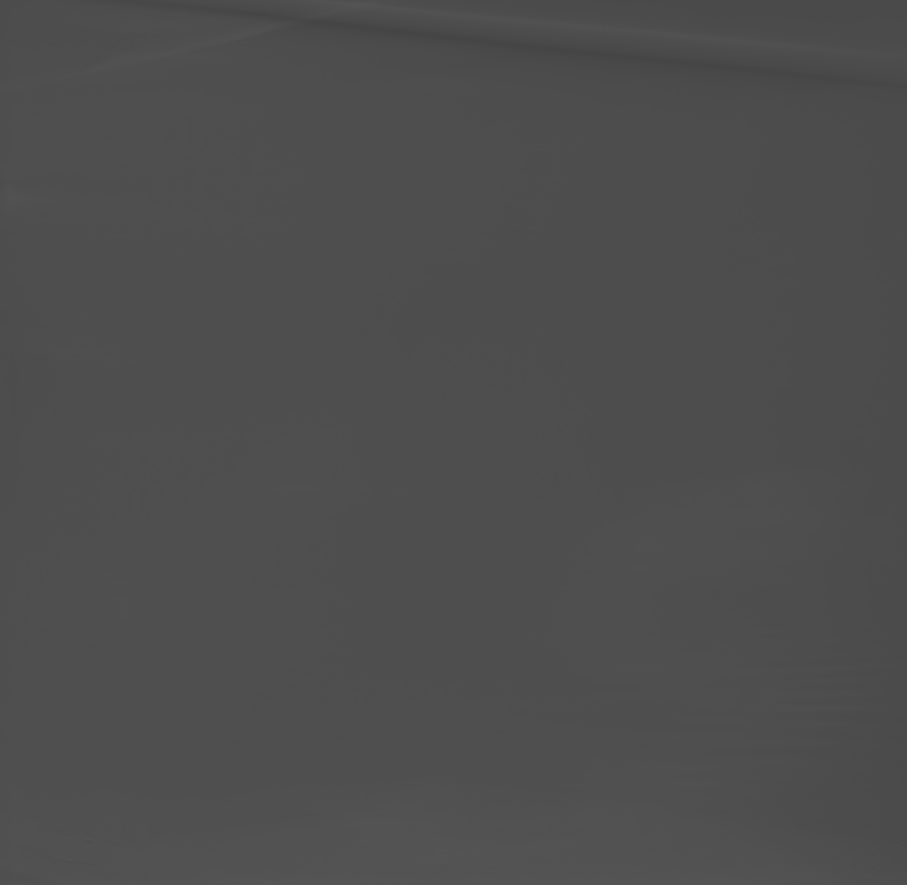

Supplement: Supplemental Information 2 — The used imagestack in the x-z-plane (bmp format) as it was obtained and used in SPIERS. [file peerj-05-3526-s002.zip › front_x-z-plane_106.bmp]
