# Supplementary figures and images for: 3D-Analysis of a non-planispiral ammonoid from the Hunsrück Slate: natural or pathological variation? (part 1 of 2)
Source: PeerJ. 2017 Jun 30;5:e3526. doi: 10.7717/peerj.3526 (PMC5494166; doi:10.7717/peerj.3526)

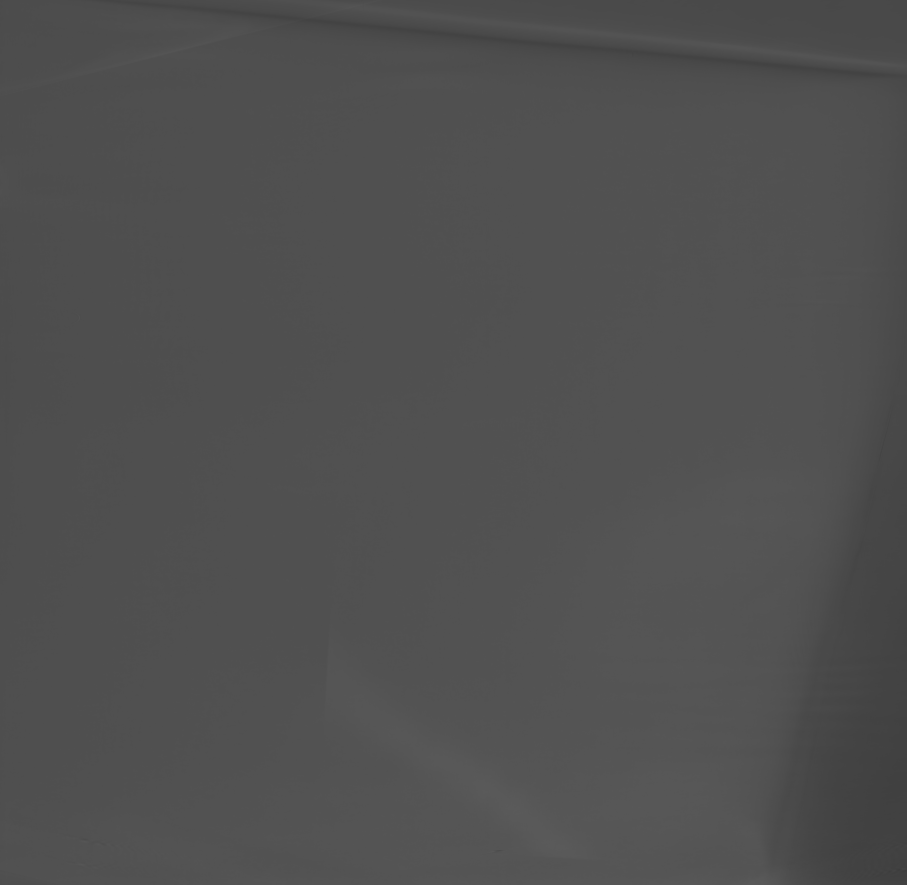

Supplement: Supplemental Information 2 — The used imagestack in the x-z-plane (bmp format) as it was obtained and used in SPIERS. [file peerj-05-3526-s002.zip › front_x-z-plane_000.bmp]

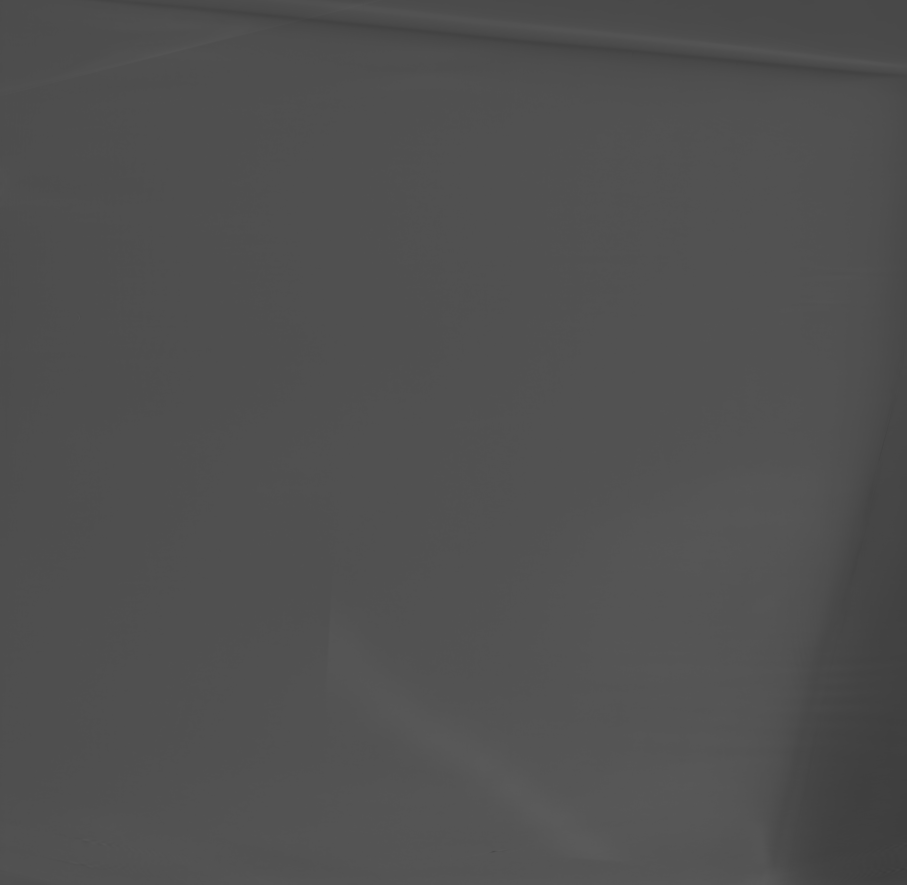

Supplement: Supplemental Information 2 — The used imagestack in the x-z-plane (bmp format) as it was obtained and used in SPIERS. [file peerj-05-3526-s002.zip › front_x-z-plane_001.bmp]

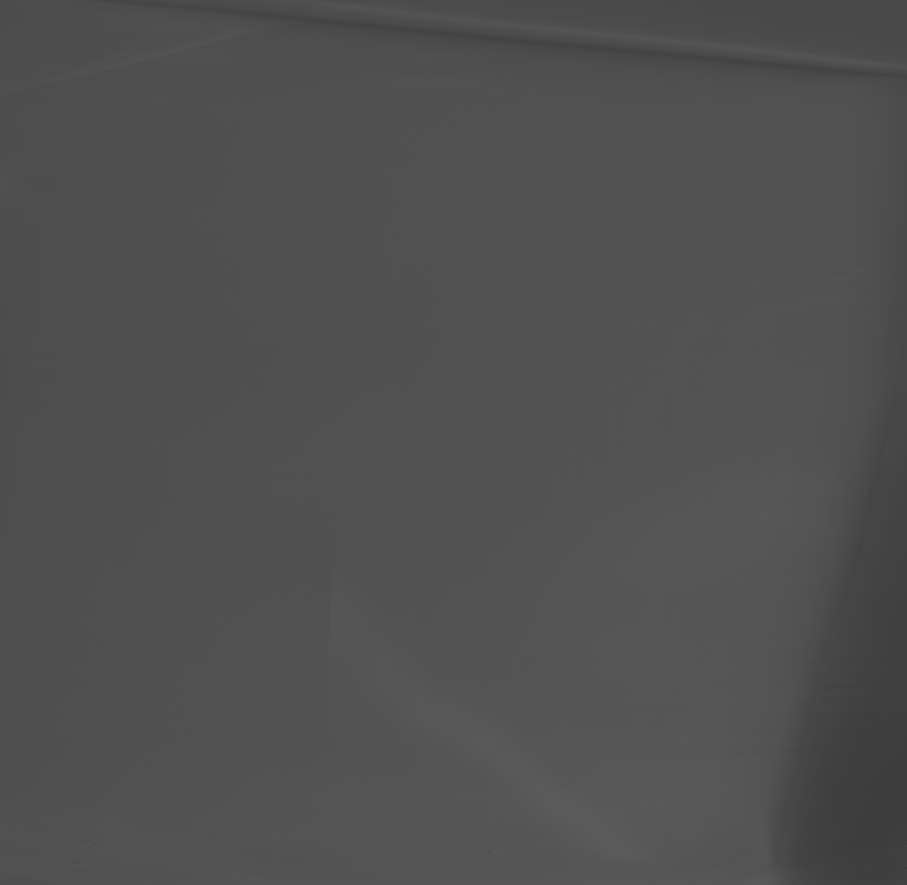

Supplement: Supplemental Information 2 — The used imagestack in the x-z-plane (bmp format) as it was obtained and used in SPIERS. [file peerj-05-3526-s002.zip › front_x-z-plane_002.bmp]

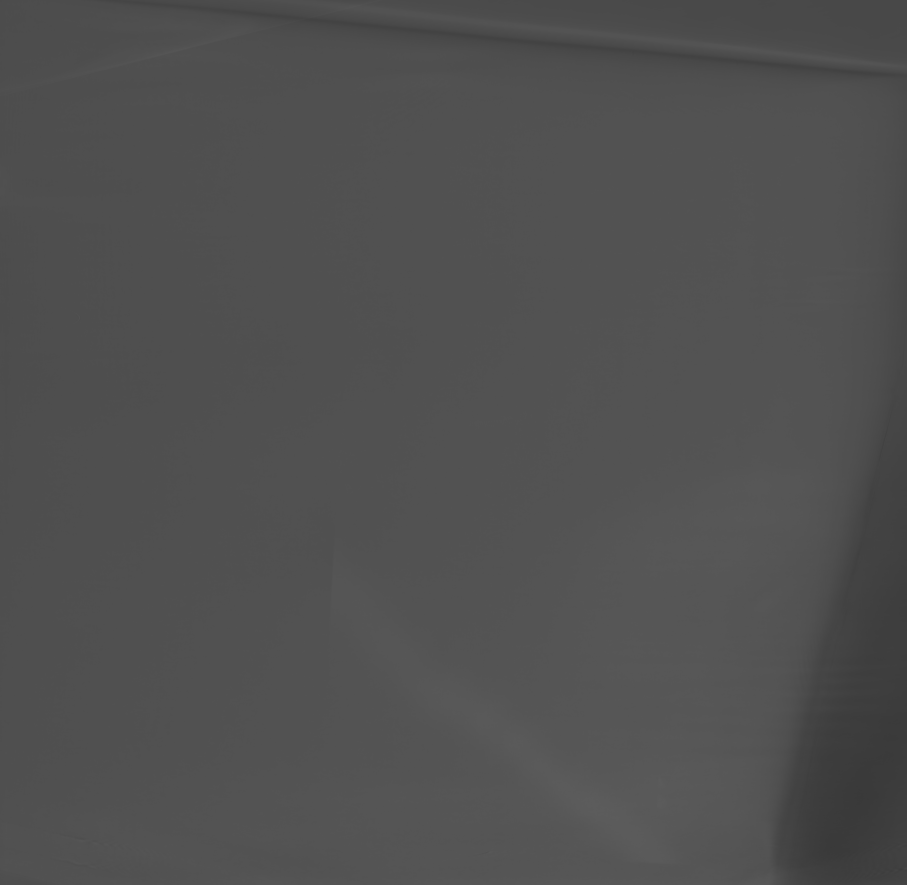

Supplement: Supplemental Information 2 — The used imagestack in the x-z-plane (bmp format) as it was obtained and used in SPIERS. [file peerj-05-3526-s002.zip › front_x-z-plane_003.bmp]

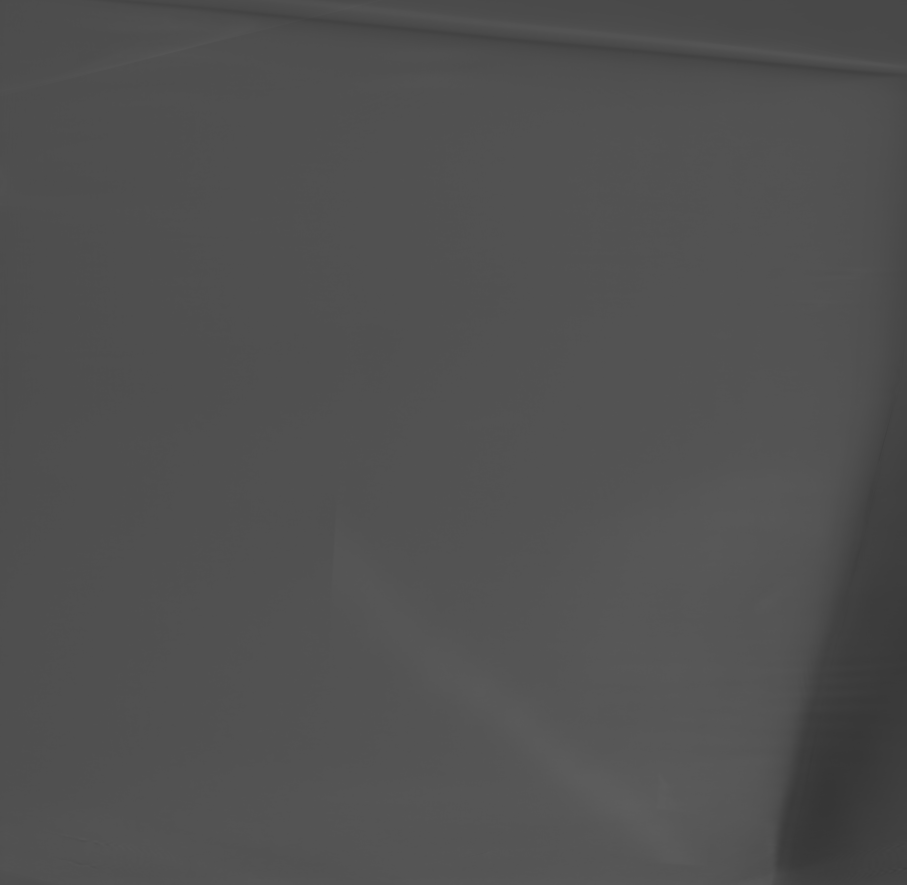

Supplement: Supplemental Information 2 — The used imagestack in the x-z-plane (bmp format) as it was obtained and used in SPIERS. [file peerj-05-3526-s002.zip › front_x-z-plane_004.bmp]

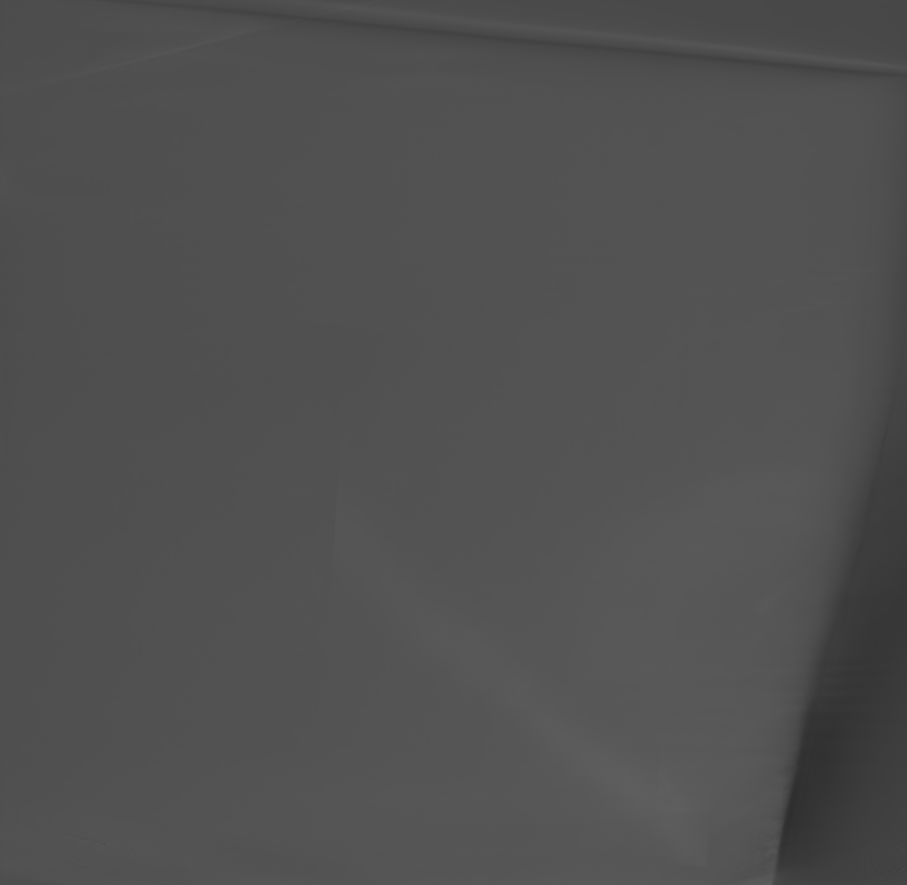

Supplement: Supplemental Information 2 — The used imagestack in the x-z-plane (bmp format) as it was obtained and used in SPIERS. [file peerj-05-3526-s002.zip › front_x-z-plane_005.bmp]

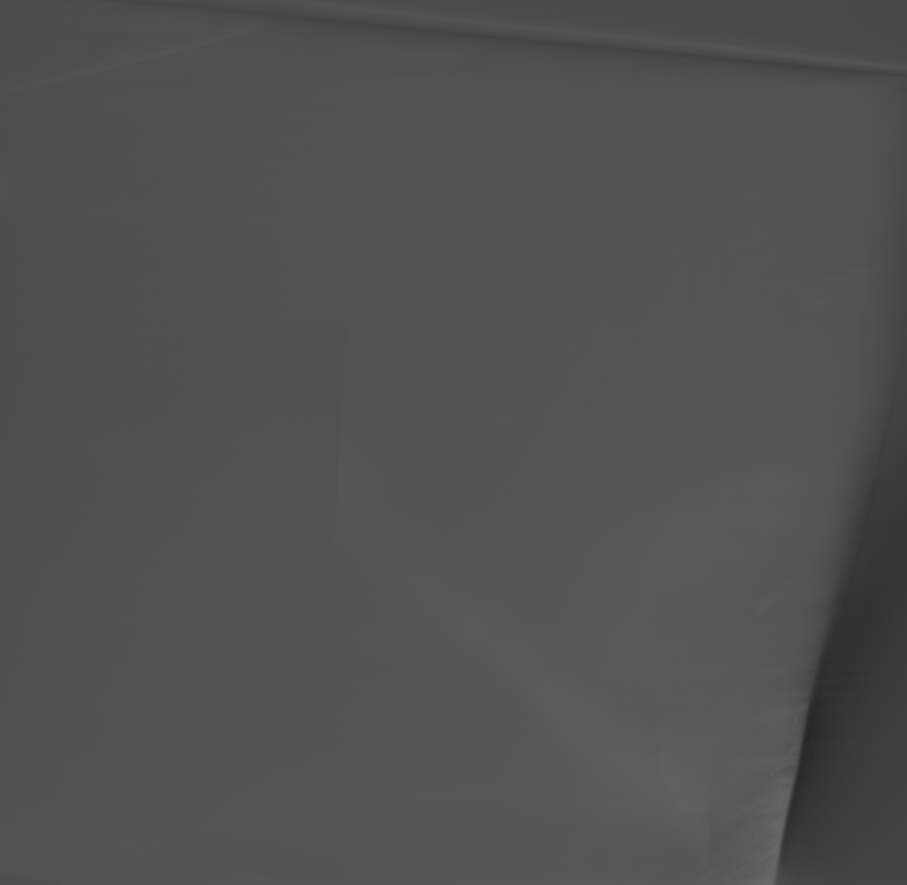

Supplement: Supplemental Information 2 — The used imagestack in the x-z-plane (bmp format) as it was obtained and used in SPIERS. [file peerj-05-3526-s002.zip › front_x-z-plane_006.bmp]

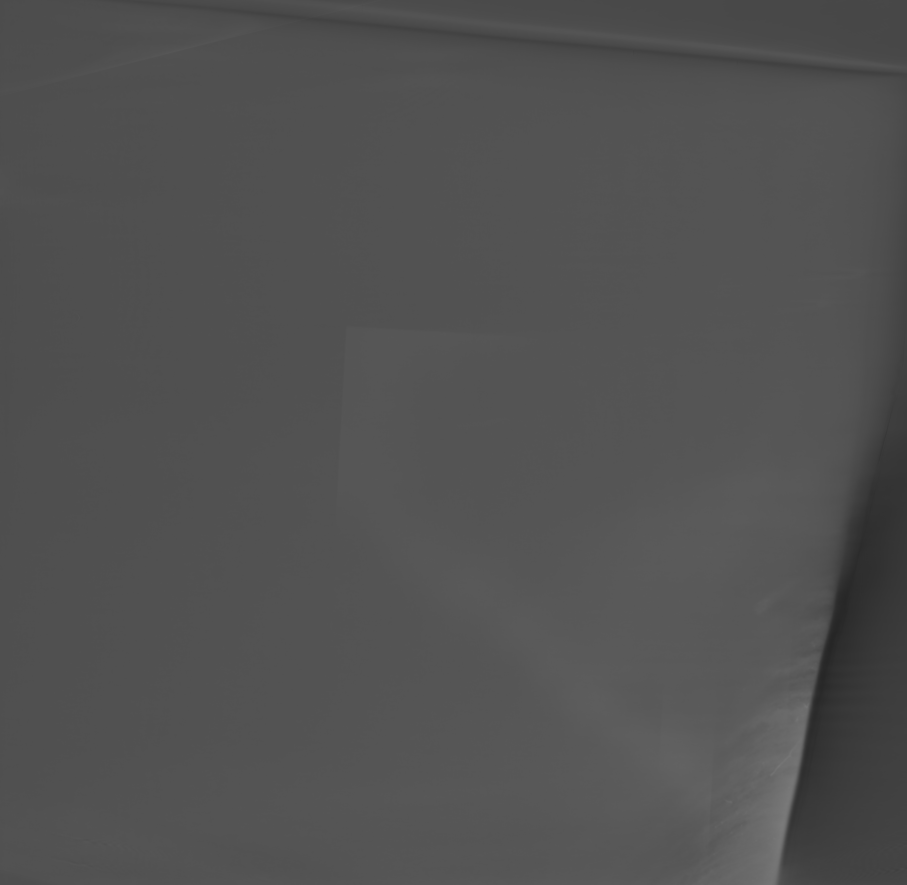

Supplement: Supplemental Information 2 — The used imagestack in the x-z-plane (bmp format) as it was obtained and used in SPIERS. [file peerj-05-3526-s002.zip › front_x-z-plane_007.bmp]

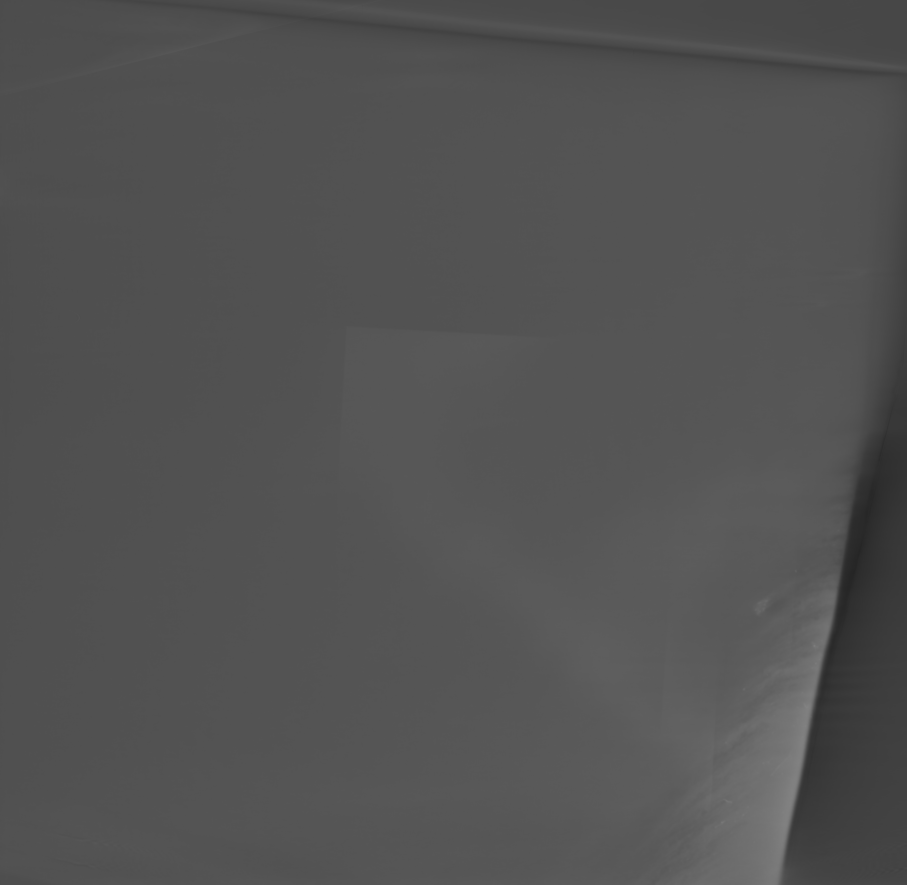

Supplement: Supplemental Information 2 — The used imagestack in the x-z-plane (bmp format) as it was obtained and used in SPIERS. [file peerj-05-3526-s002.zip › front_x-z-plane_008.bmp]

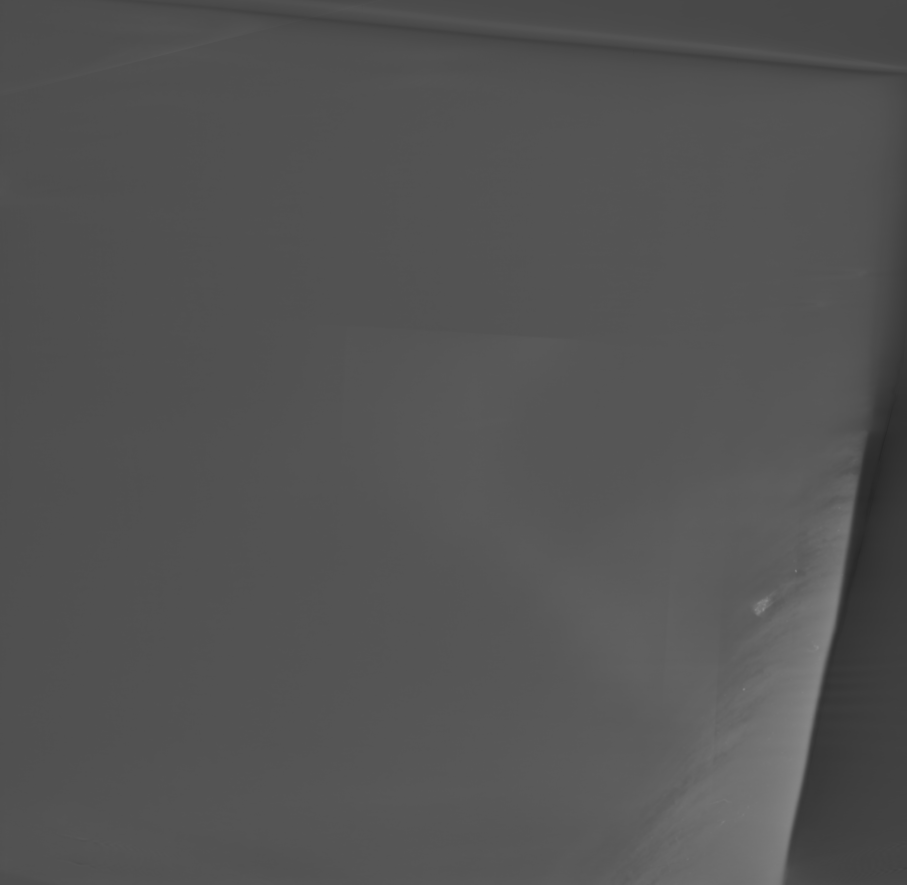

Supplement: Supplemental Information 2 — The used imagestack in the x-z-plane (bmp format) as it was obtained and used in SPIERS. [file peerj-05-3526-s002.zip › front_x-z-plane_009.bmp]

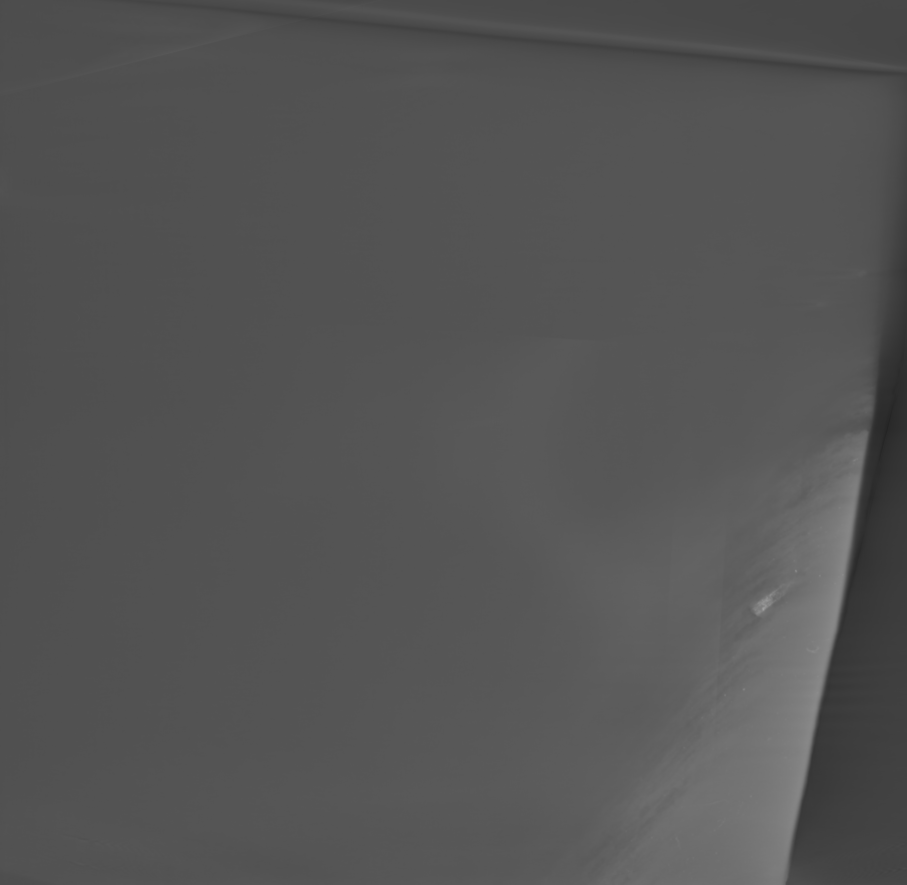

Supplement: Supplemental Information 2 — The used imagestack in the x-z-plane (bmp format) as it was obtained and used in SPIERS. [file peerj-05-3526-s002.zip › front_x-z-plane_010.bmp]

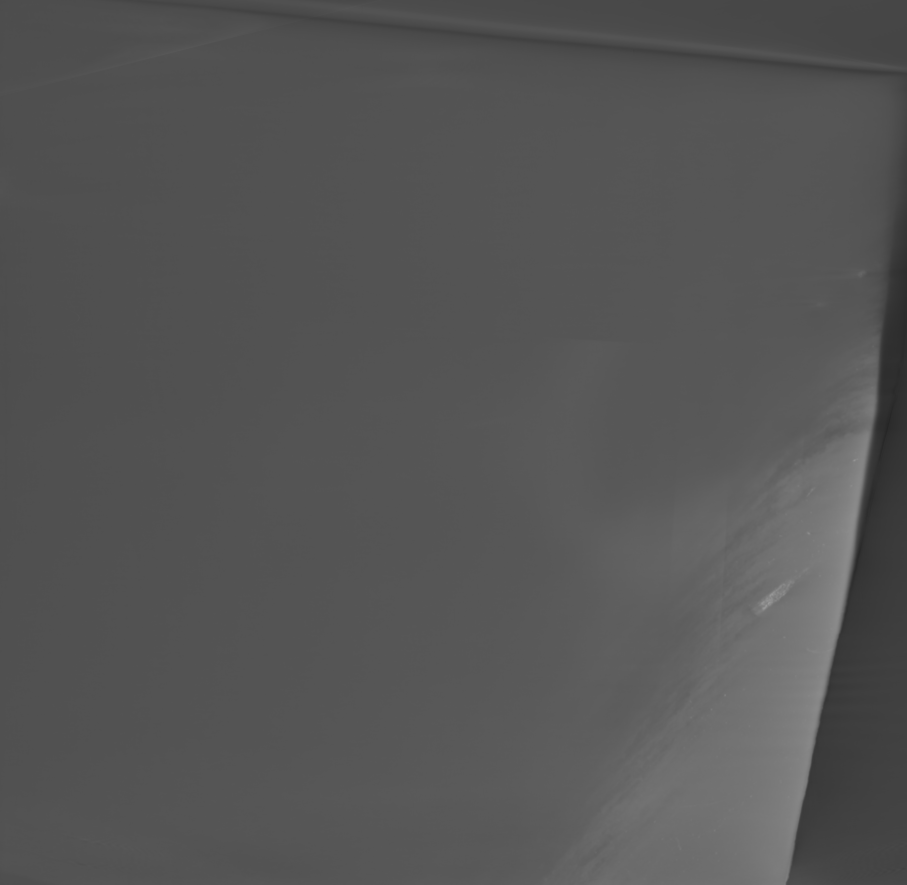

Supplement: Supplemental Information 2 — The used imagestack in the x-z-plane (bmp format) as it was obtained and used in SPIERS. [file peerj-05-3526-s002.zip › front_x-z-plane_011.bmp]

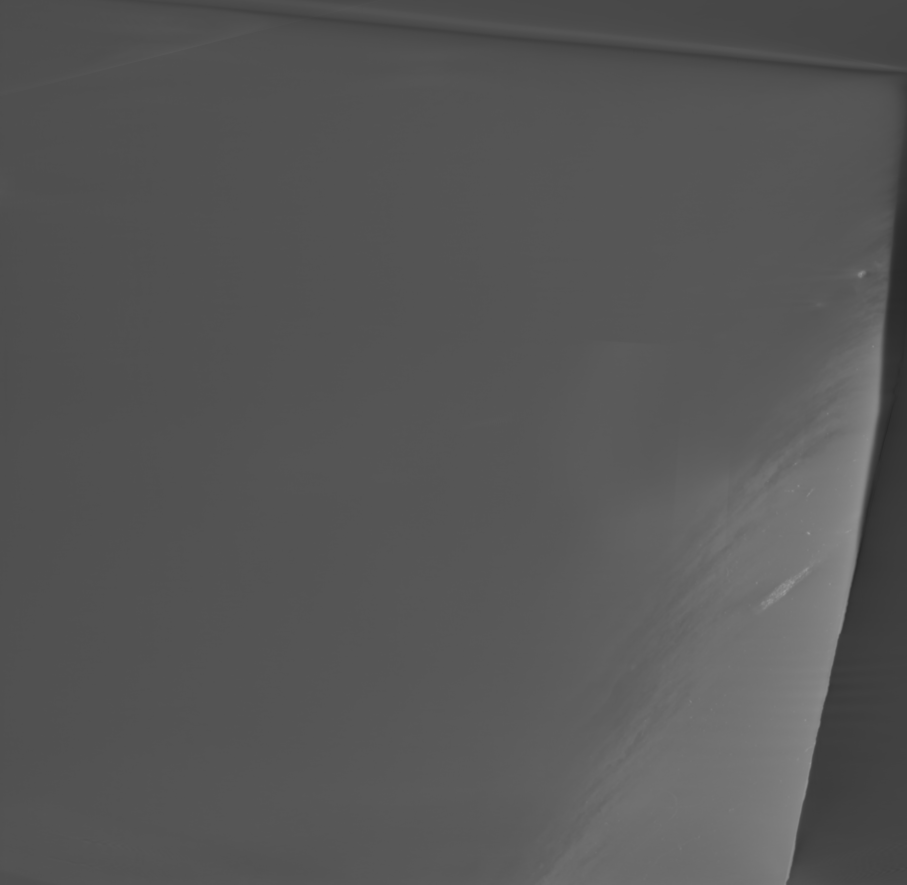

Supplement: Supplemental Information 2 — The used imagestack in the x-z-plane (bmp format) as it was obtained and used in SPIERS. [file peerj-05-3526-s002.zip › front_x-z-plane_012.bmp]

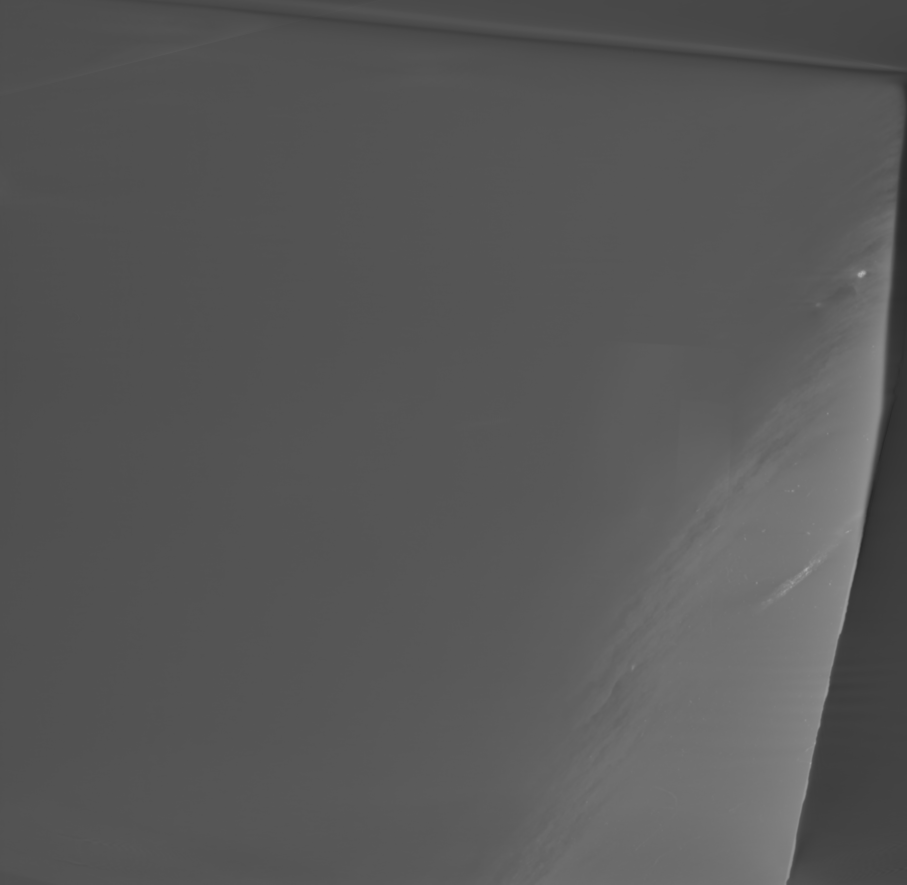

Supplement: Supplemental Information 2 — The used imagestack in the x-z-plane (bmp format) as it was obtained and used in SPIERS. [file peerj-05-3526-s002.zip › front_x-z-plane_013.bmp]

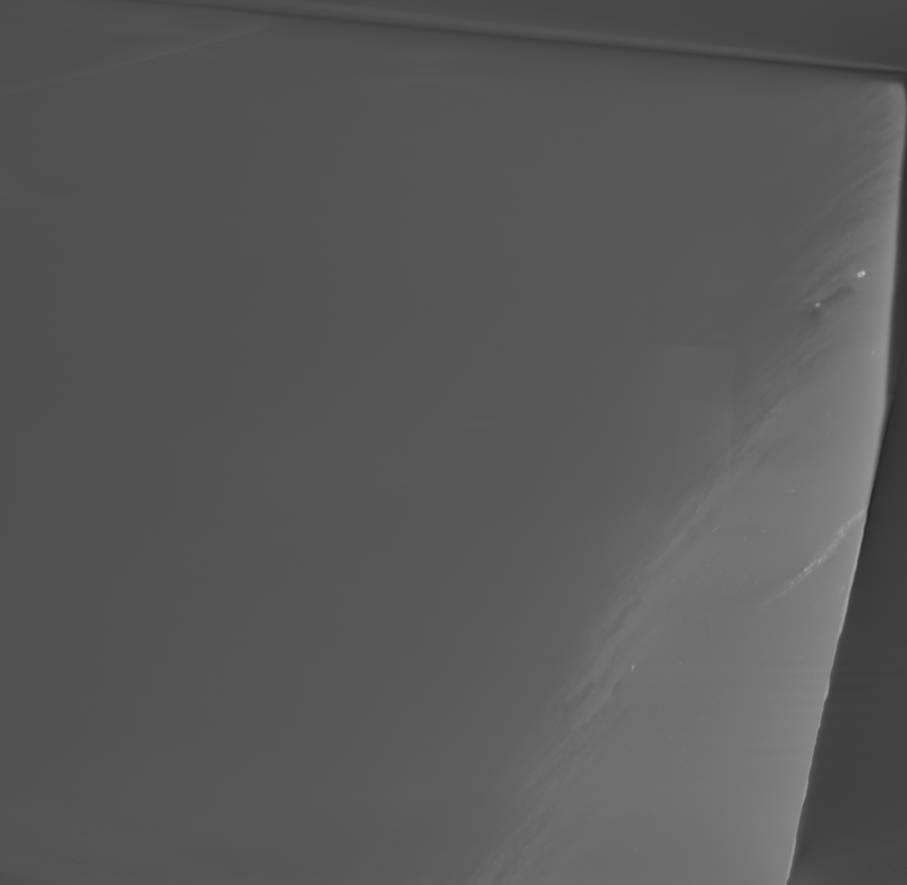

Supplement: Supplemental Information 2 — The used imagestack in the x-z-plane (bmp format) as it was obtained and used in SPIERS. [file peerj-05-3526-s002.zip › front_x-z-plane_014.bmp]

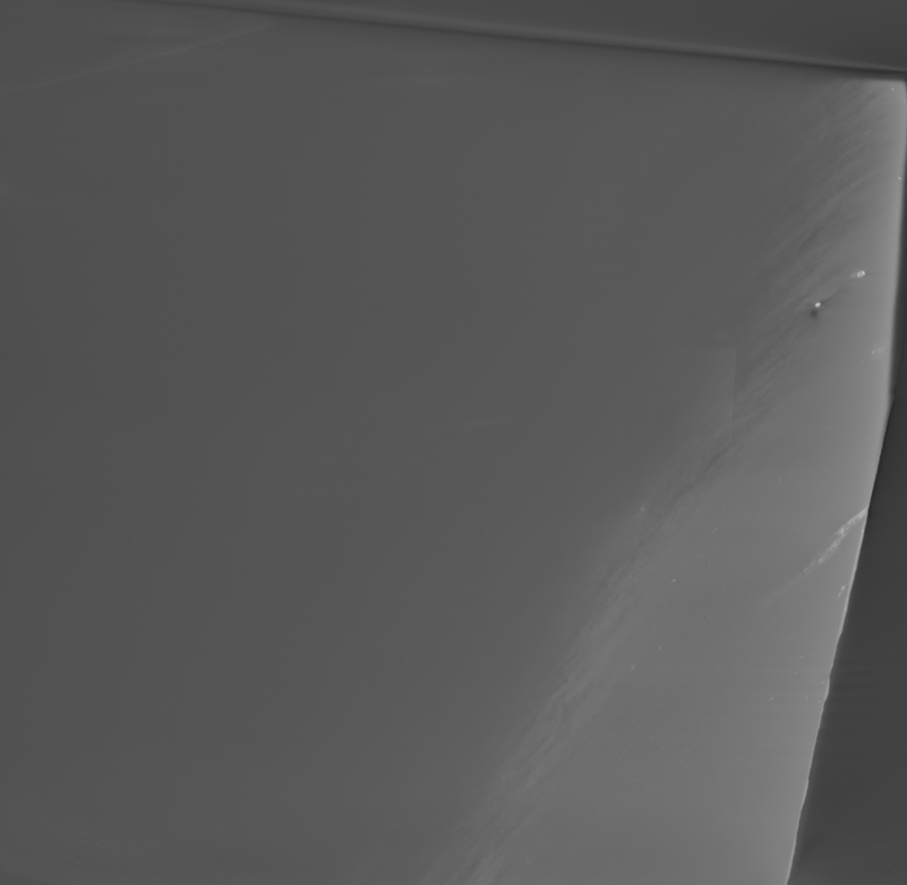

Supplement: Supplemental Information 2 — The used imagestack in the x-z-plane (bmp format) as it was obtained and used in SPIERS. [file peerj-05-3526-s002.zip › front_x-z-plane_015.bmp]

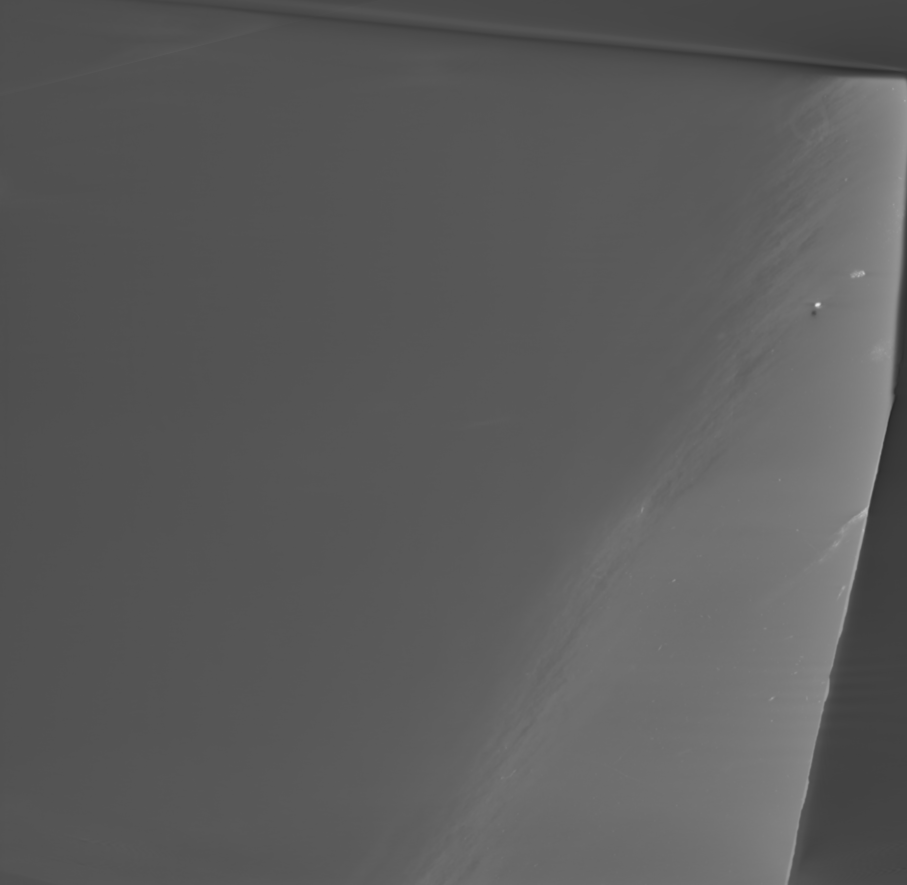

Supplement: Supplemental Information 2 — The used imagestack in the x-z-plane (bmp format) as it was obtained and used in SPIERS. [file peerj-05-3526-s002.zip › front_x-z-plane_016.bmp]

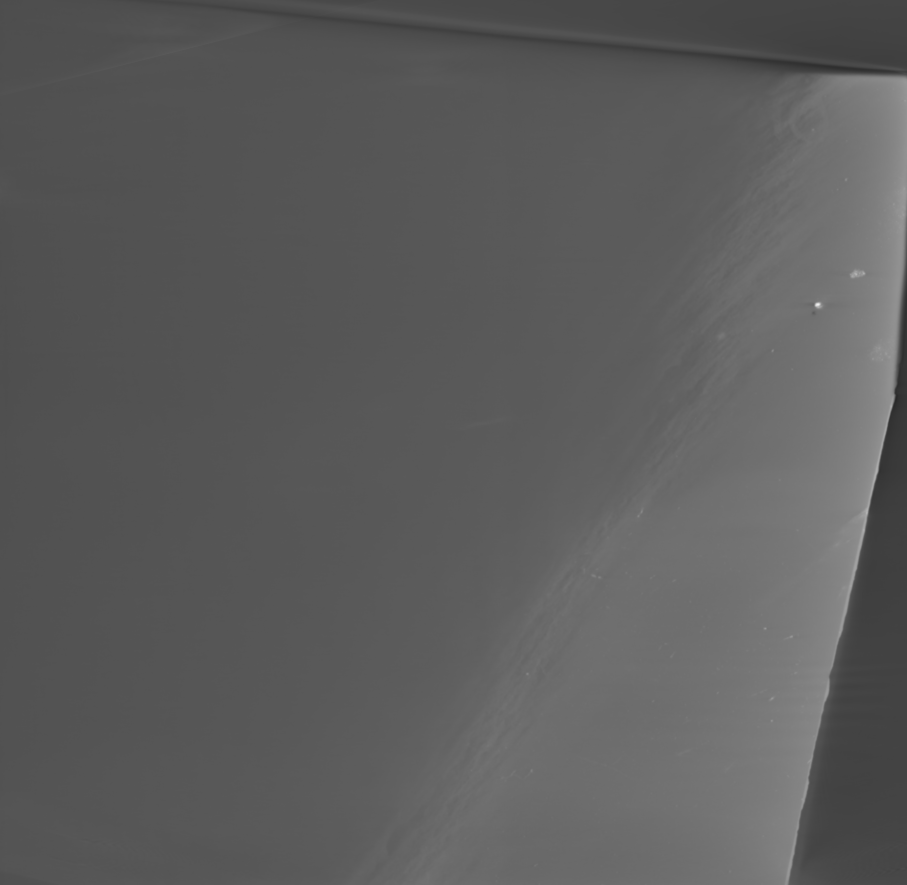

Supplement: Supplemental Information 2 — The used imagestack in the x-z-plane (bmp format) as it was obtained and used in SPIERS. [file peerj-05-3526-s002.zip › front_x-z-plane_017.bmp]

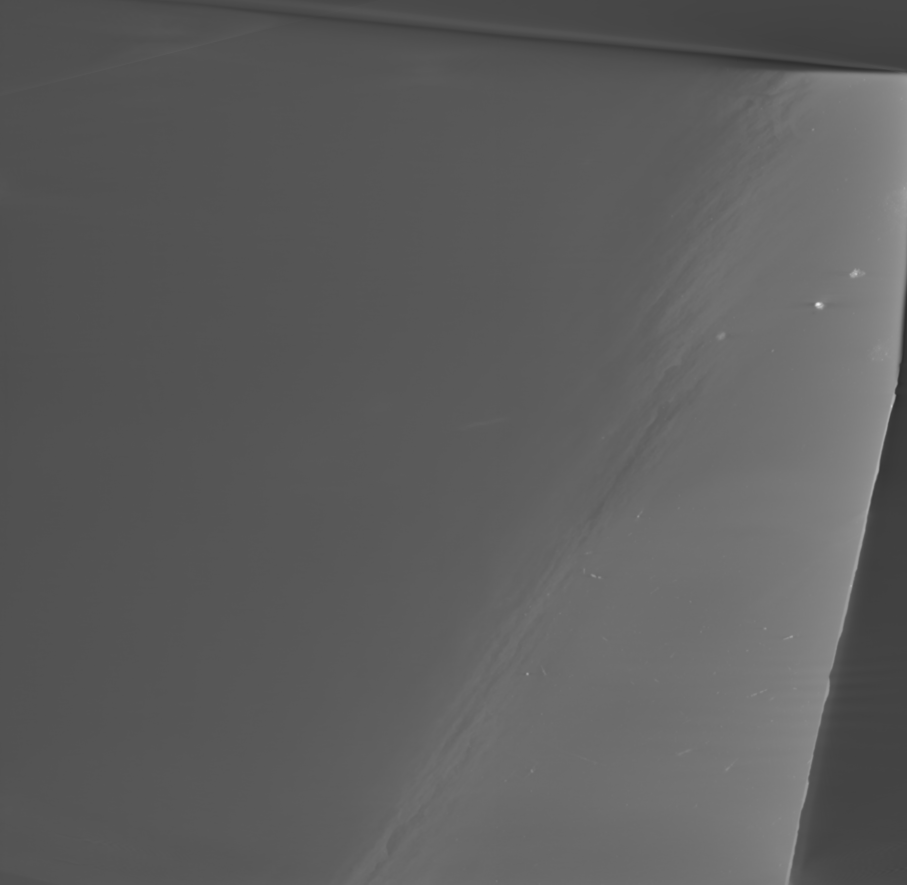

Supplement: Supplemental Information 2 — The used imagestack in the x-z-plane (bmp format) as it was obtained and used in SPIERS. [file peerj-05-3526-s002.zip › front_x-z-plane_018.bmp]

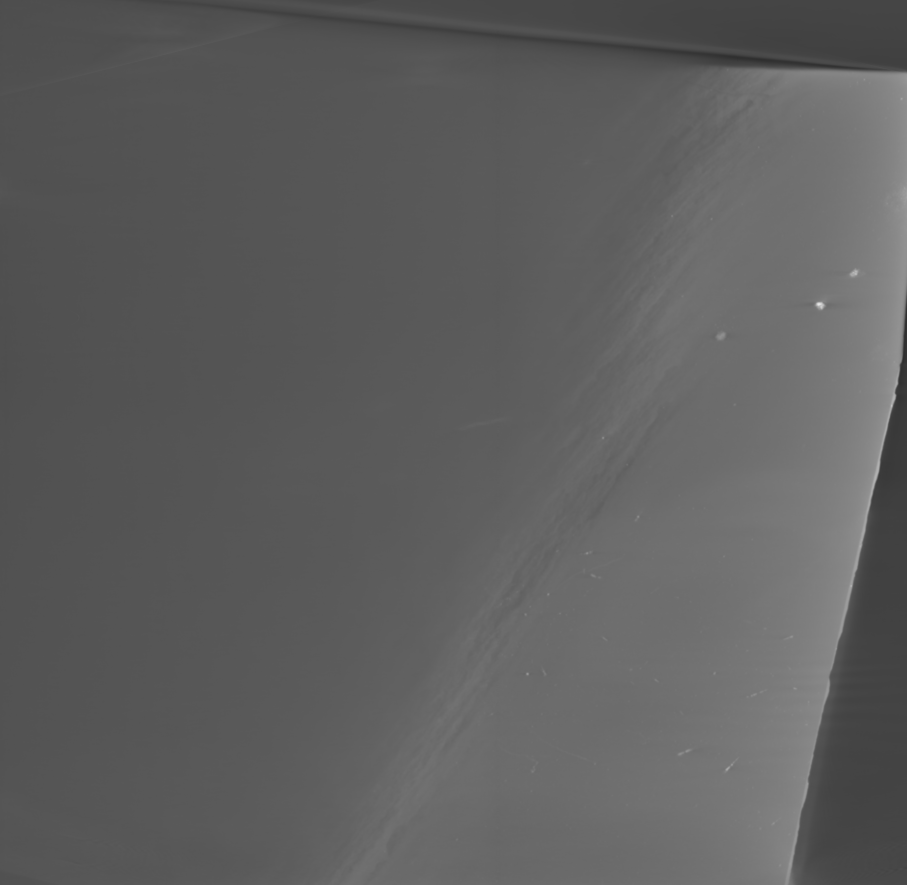

Supplement: Supplemental Information 2 — The used imagestack in the x-z-plane (bmp format) as it was obtained and used in SPIERS. [file peerj-05-3526-s002.zip › front_x-z-plane_019.bmp]

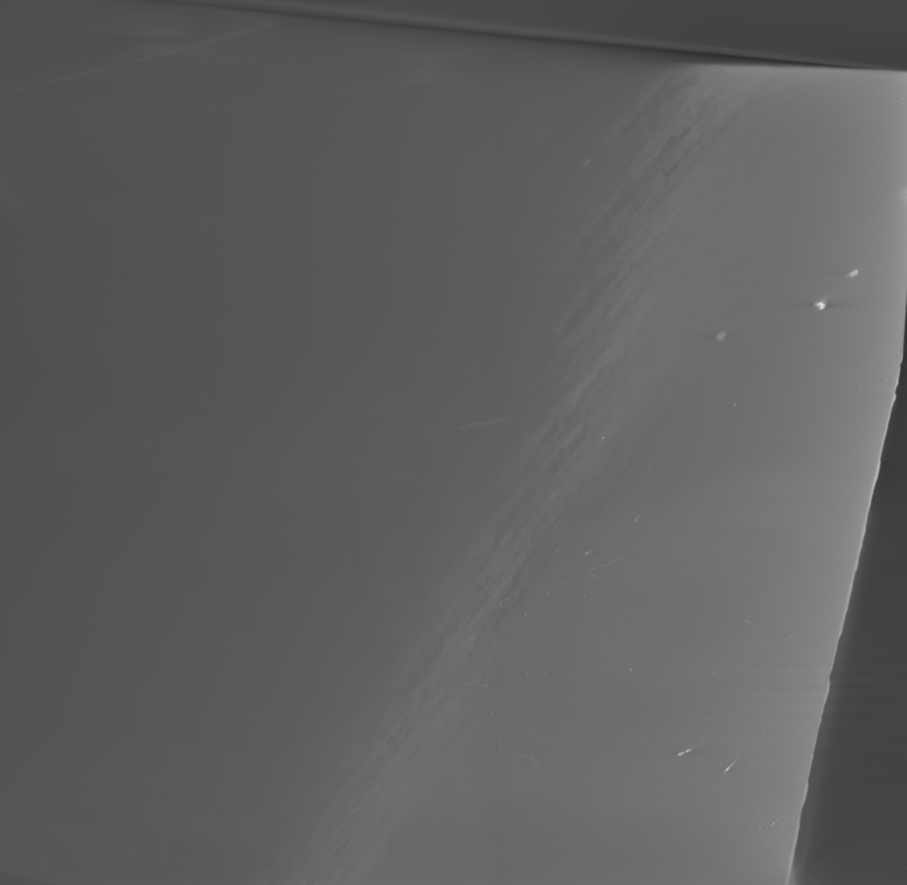

Supplement: Supplemental Information 2 — The used imagestack in the x-z-plane (bmp format) as it was obtained and used in SPIERS. [file peerj-05-3526-s002.zip › front_x-z-plane_020.bmp]

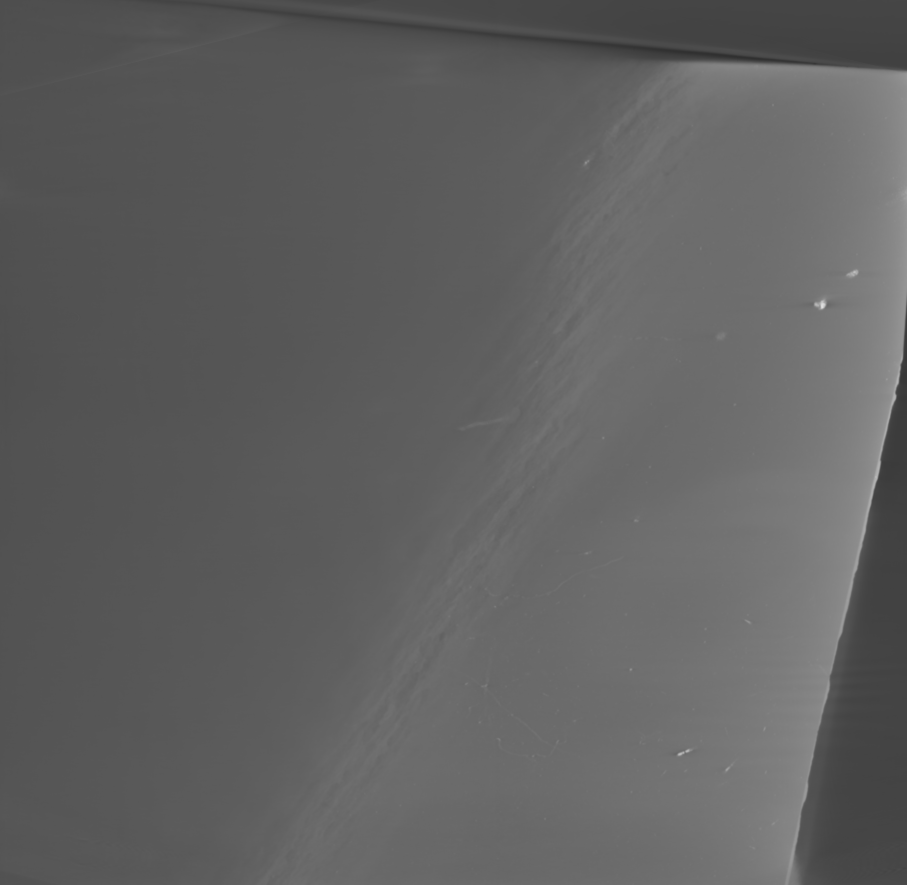

Supplement: Supplemental Information 2 — The used imagestack in the x-z-plane (bmp format) as it was obtained and used in SPIERS. [file peerj-05-3526-s002.zip › front_x-z-plane_021.bmp]

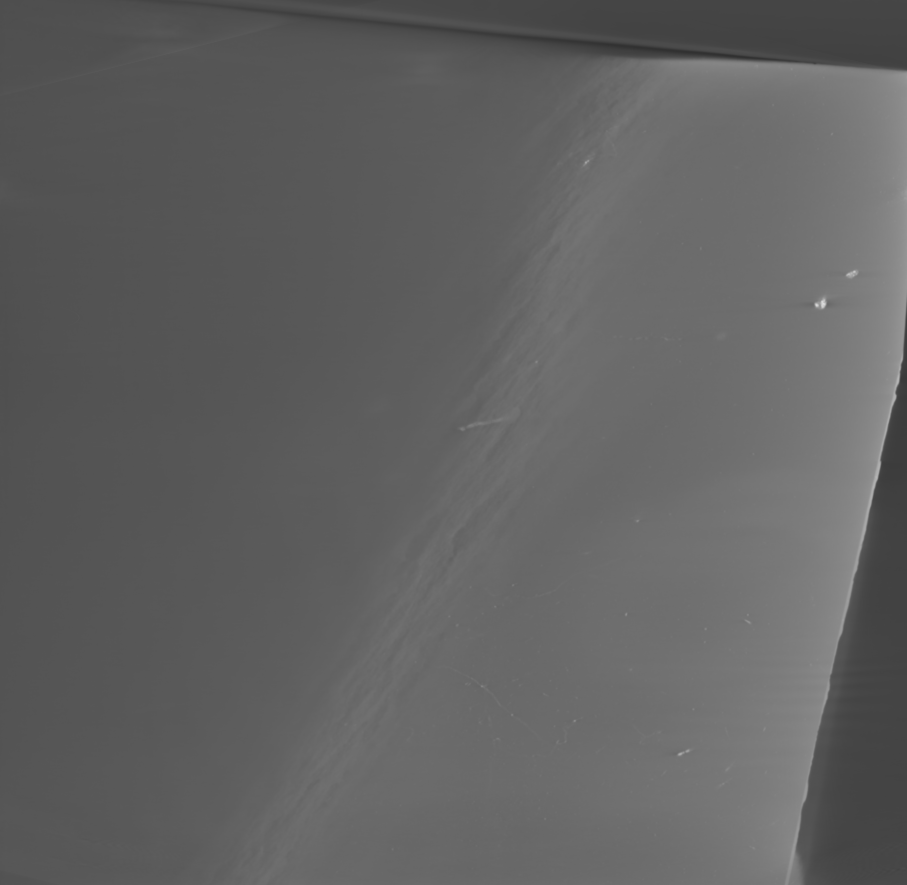

Supplement: Supplemental Information 2 — The used imagestack in the x-z-plane (bmp format) as it was obtained and used in SPIERS. [file peerj-05-3526-s002.zip › front_x-z-plane_022.bmp]

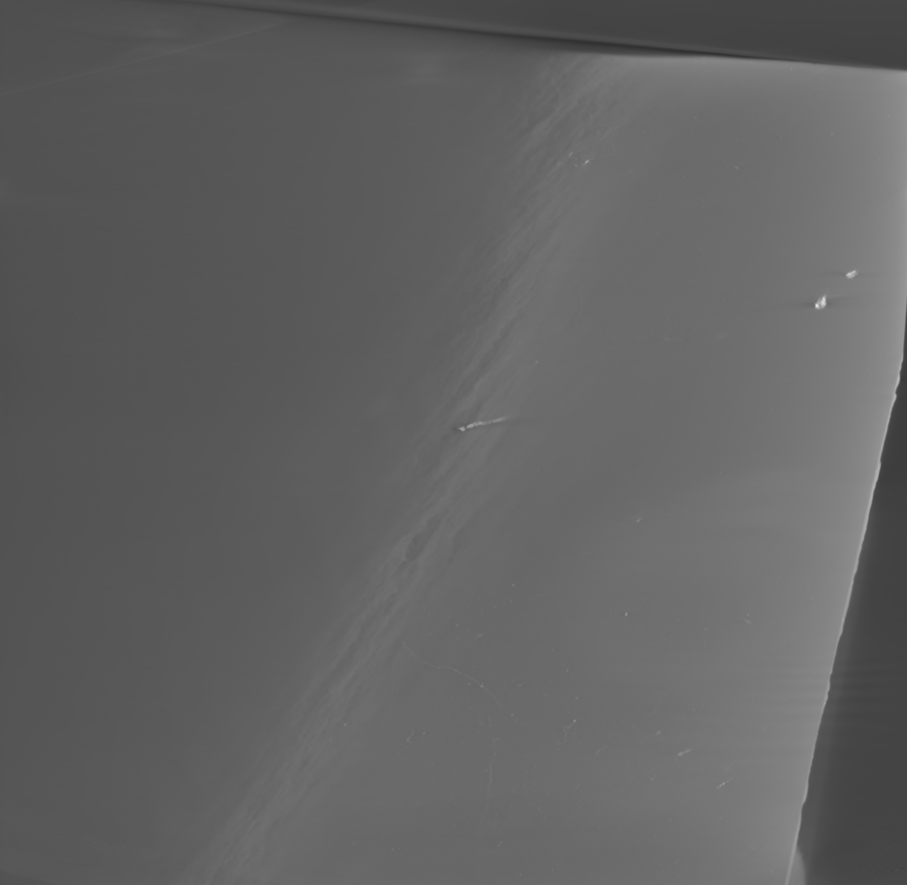

Supplement: Supplemental Information 2 — The used imagestack in the x-z-plane (bmp format) as it was obtained and used in SPIERS. [file peerj-05-3526-s002.zip › front_x-z-plane_023.bmp]

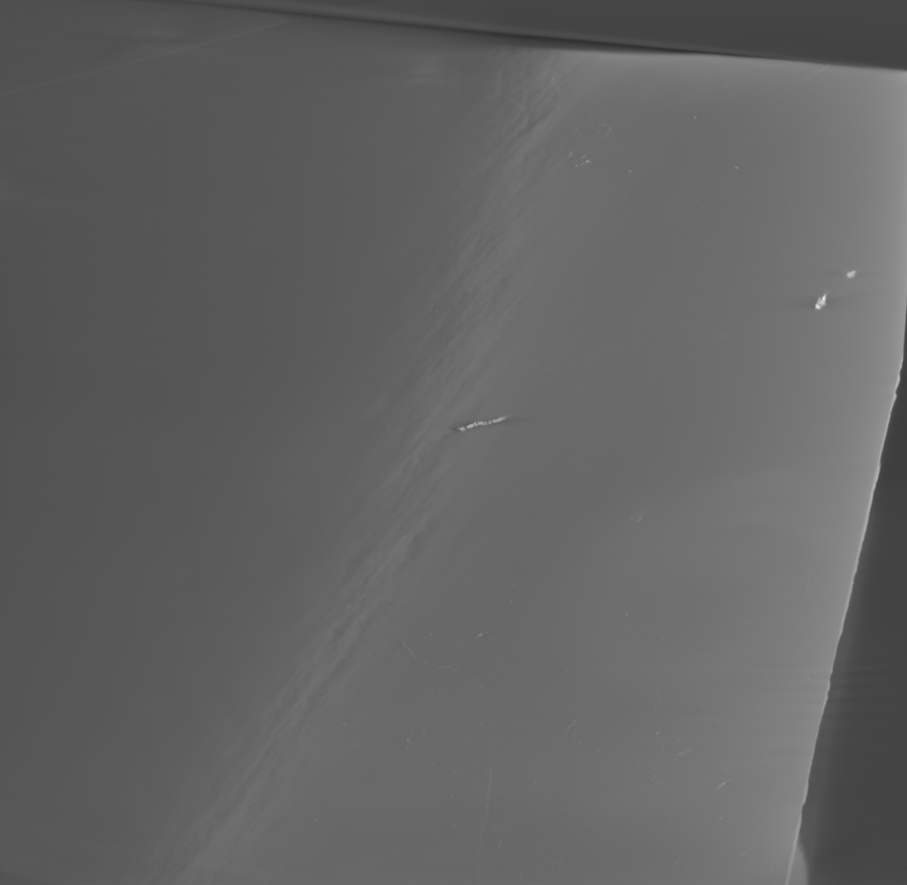

Supplement: Supplemental Information 2 — The used imagestack in the x-z-plane (bmp format) as it was obtained and used in SPIERS. [file peerj-05-3526-s002.zip › front_x-z-plane_024.bmp]

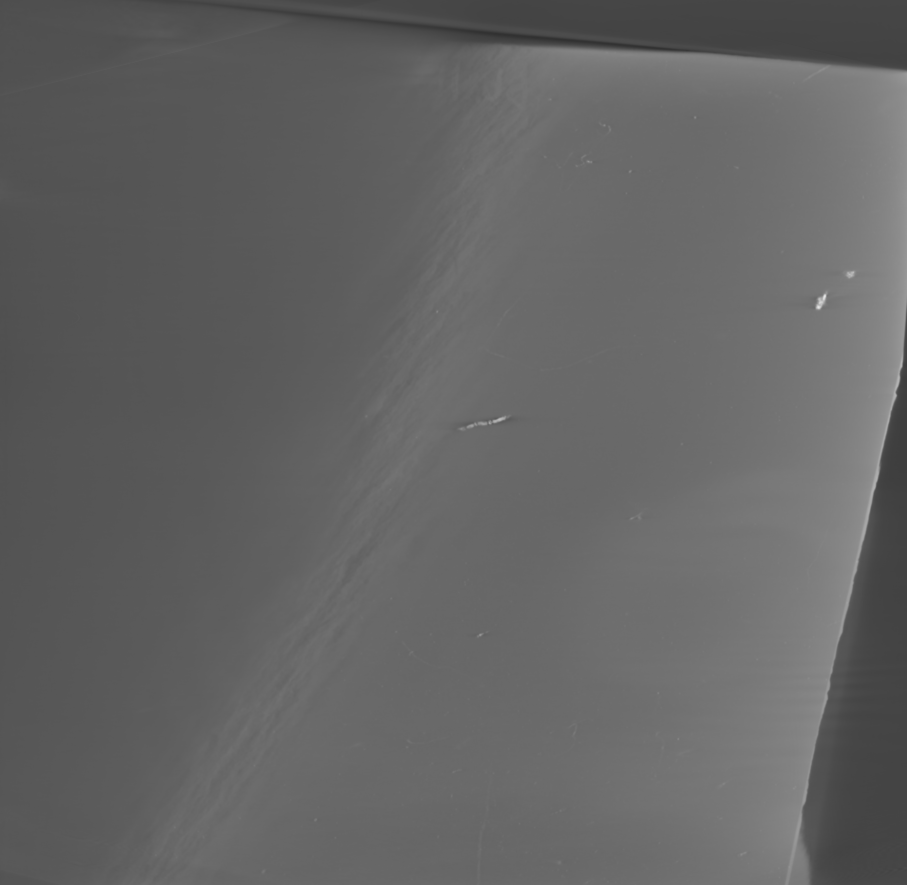

Supplement: Supplemental Information 2 — The used imagestack in the x-z-plane (bmp format) as it was obtained and used in SPIERS. [file peerj-05-3526-s002.zip › front_x-z-plane_025.bmp]

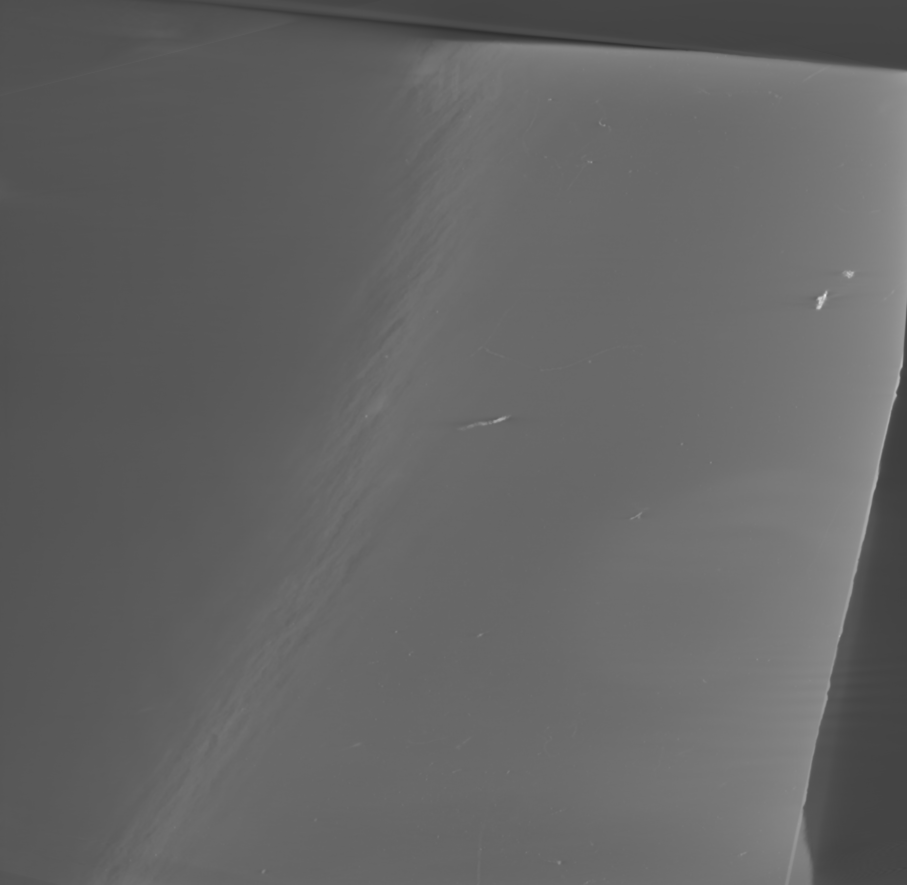

Supplement: Supplemental Information 2 — The used imagestack in the x-z-plane (bmp format) as it was obtained and used in SPIERS. [file peerj-05-3526-s002.zip › front_x-z-plane_026.bmp]

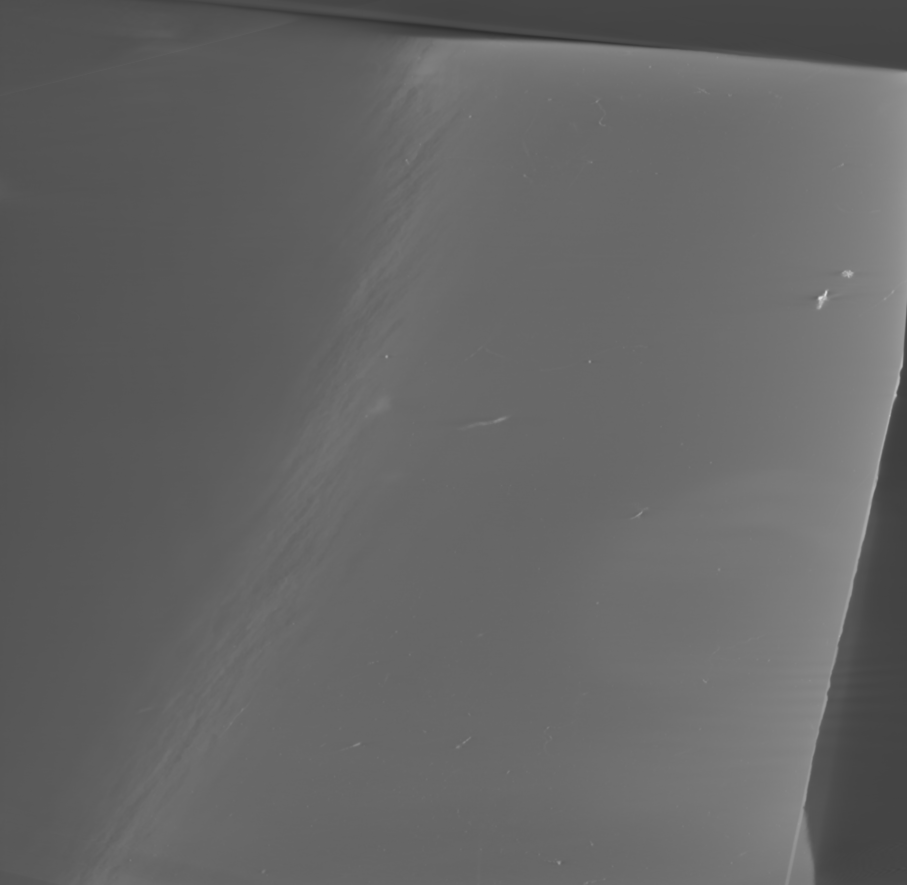

Supplement: Supplemental Information 2 — The used imagestack in the x-z-plane (bmp format) as it was obtained and used in SPIERS. [file peerj-05-3526-s002.zip › front_x-z-plane_027.bmp]

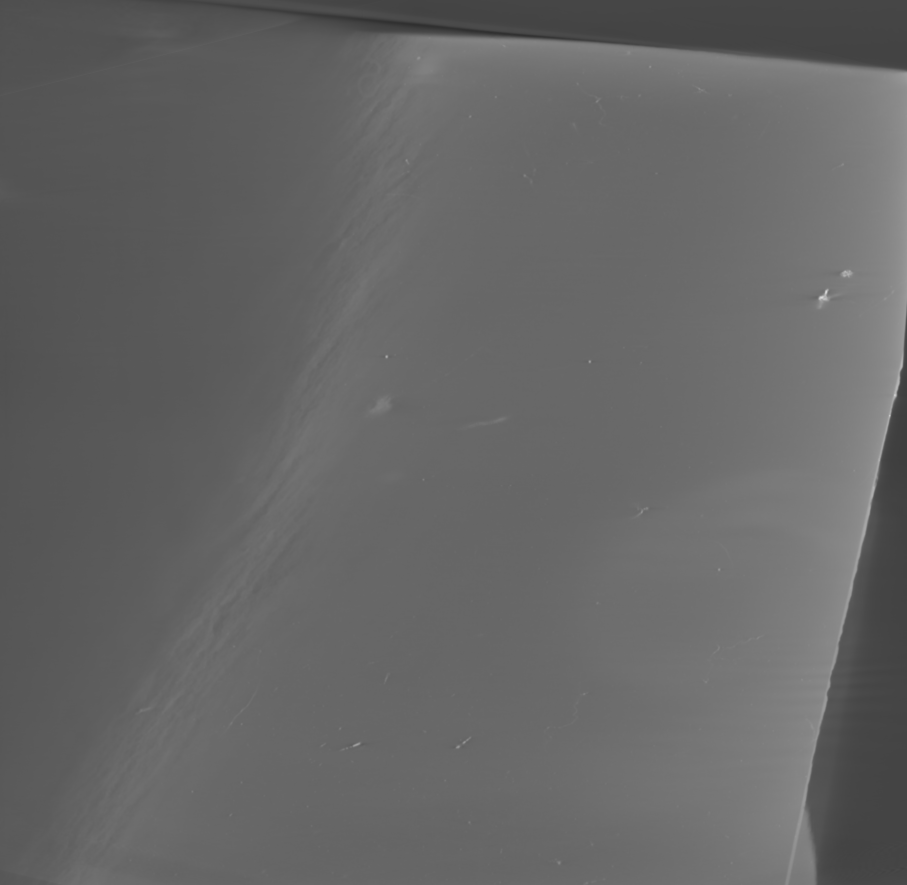

Supplement: Supplemental Information 2 — The used imagestack in the x-z-plane (bmp format) as it was obtained and used in SPIERS. [file peerj-05-3526-s002.zip › front_x-z-plane_028.bmp]

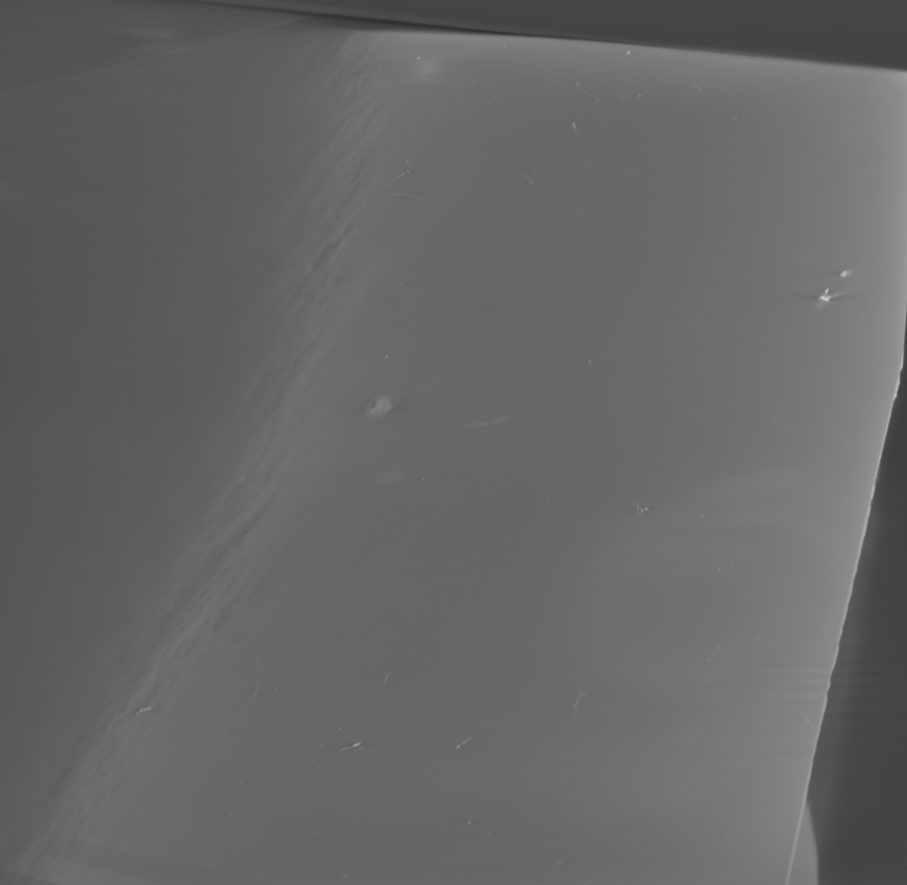

Supplement: Supplemental Information 2 — The used imagestack in the x-z-plane (bmp format) as it was obtained and used in SPIERS. [file peerj-05-3526-s002.zip › front_x-z-plane_029.bmp]

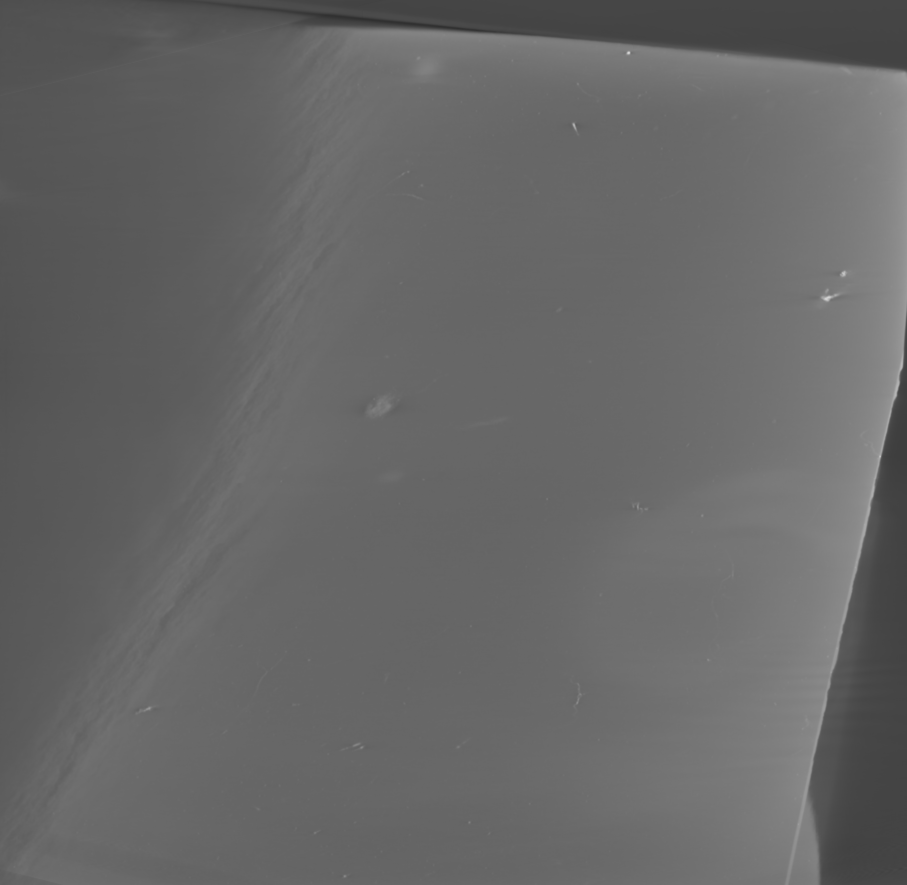

Supplement: Supplemental Information 2 — The used imagestack in the x-z-plane (bmp format) as it was obtained and used in SPIERS. [file peerj-05-3526-s002.zip › front_x-z-plane_030.bmp]

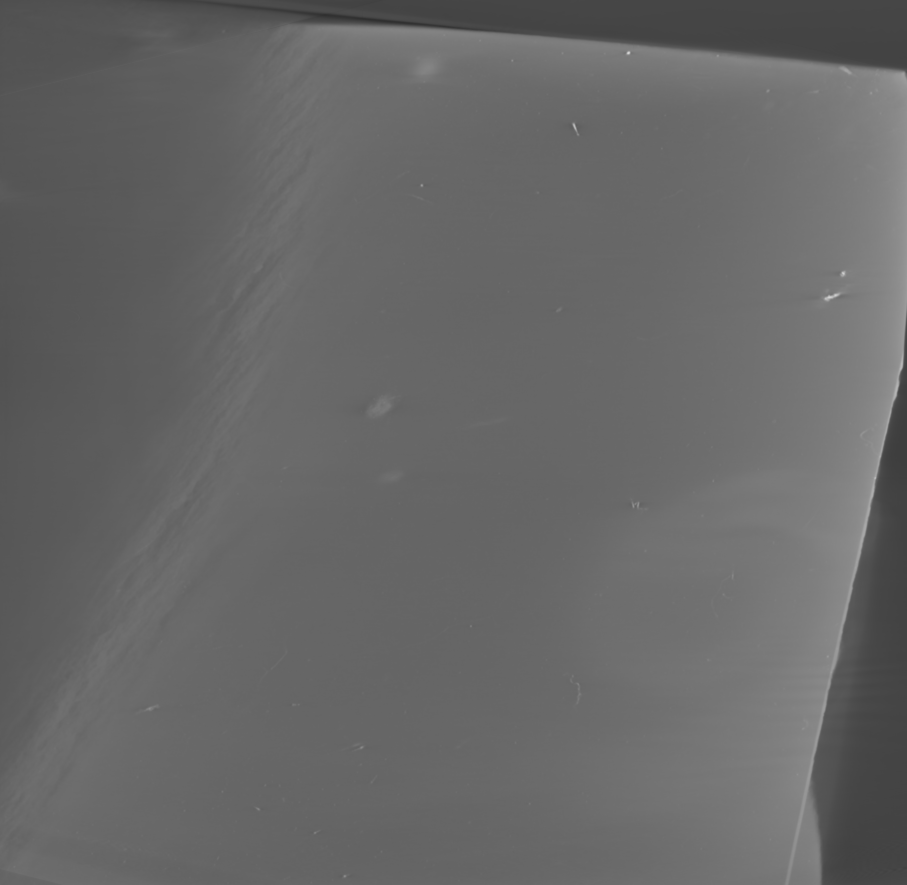

Supplement: Supplemental Information 2 — The used imagestack in the x-z-plane (bmp format) as it was obtained and used in SPIERS. [file peerj-05-3526-s002.zip › front_x-z-plane_031.bmp]

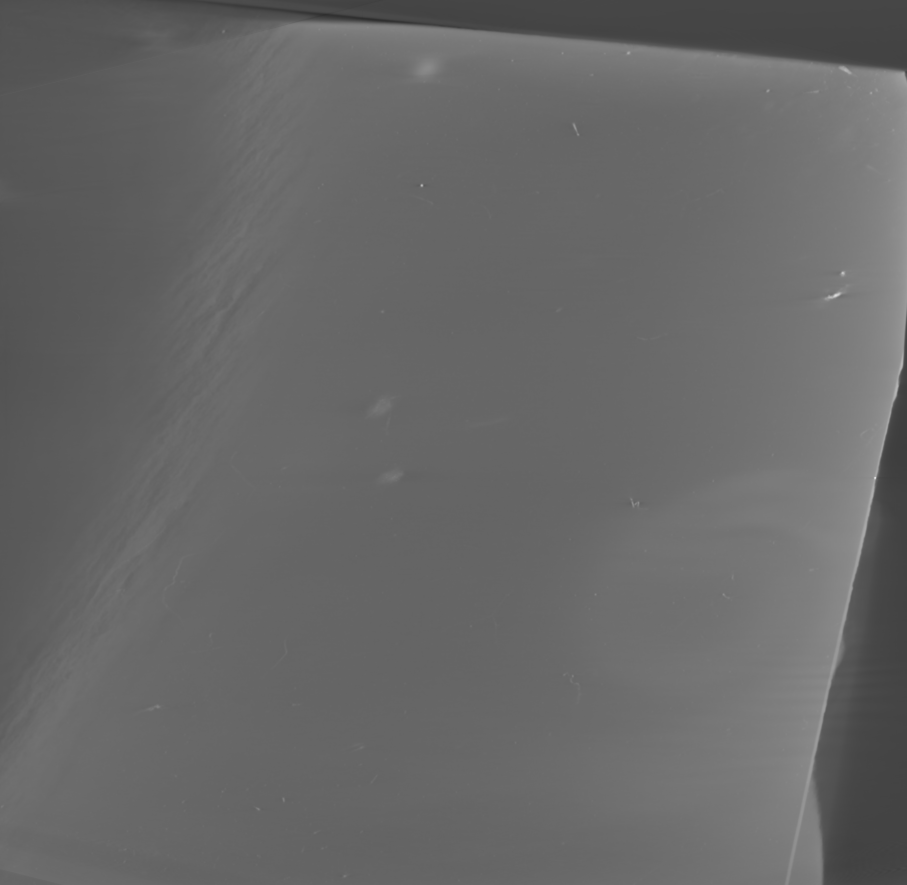

Supplement: Supplemental Information 2 — The used imagestack in the x-z-plane (bmp format) as it was obtained and used in SPIERS. [file peerj-05-3526-s002.zip › front_x-z-plane_032.bmp]

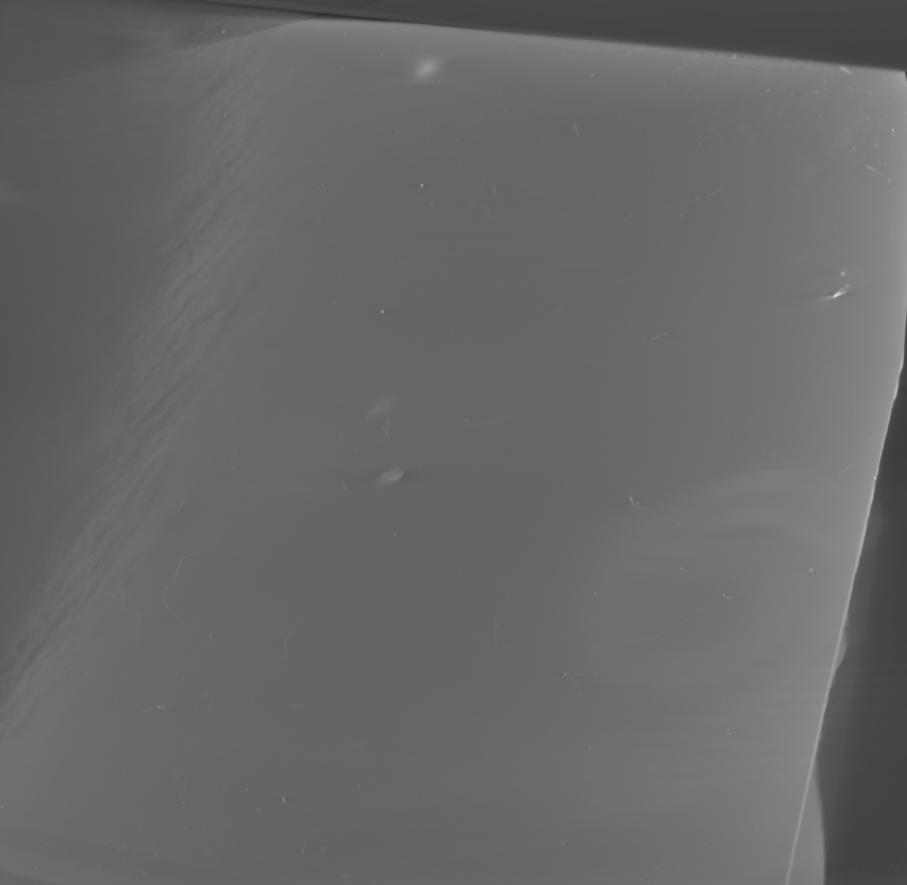

Supplement: Supplemental Information 2 — The used imagestack in the x-z-plane (bmp format) as it was obtained and used in SPIERS. [file peerj-05-3526-s002.zip › front_x-z-plane_033.bmp]

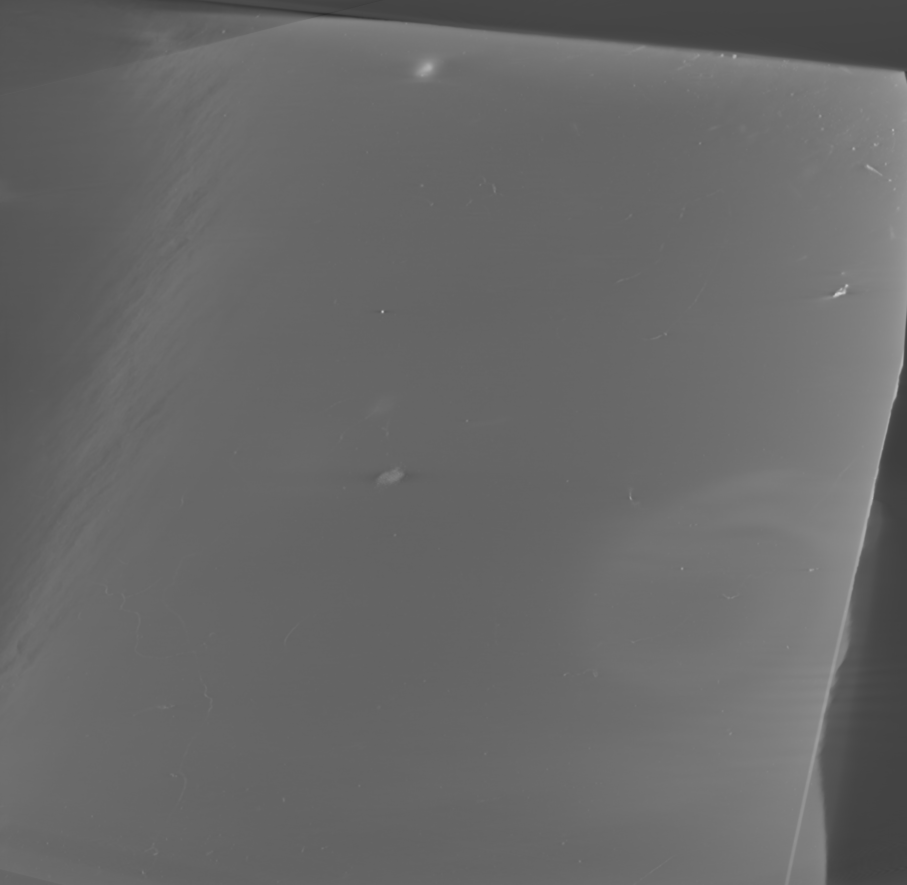

Supplement: Supplemental Information 2 — The used imagestack in the x-z-plane (bmp format) as it was obtained and used in SPIERS. [file peerj-05-3526-s002.zip › front_x-z-plane_034.bmp]

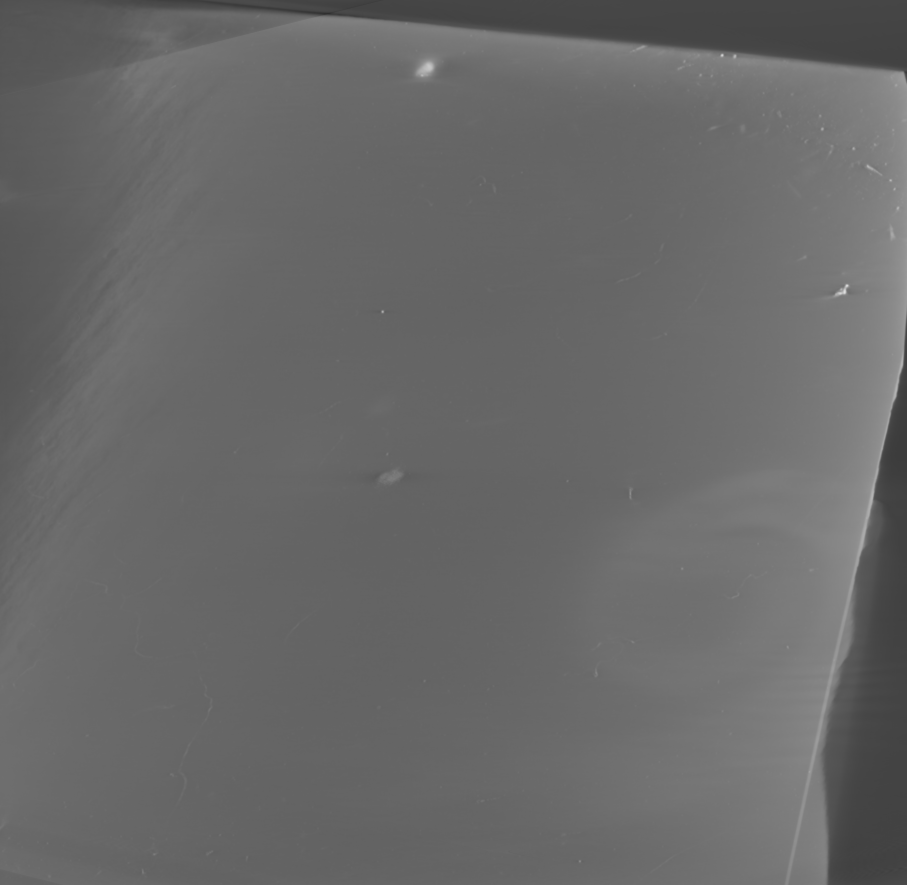

Supplement: Supplemental Information 2 — The used imagestack in the x-z-plane (bmp format) as it was obtained and used in SPIERS. [file peerj-05-3526-s002.zip › front_x-z-plane_035.bmp]

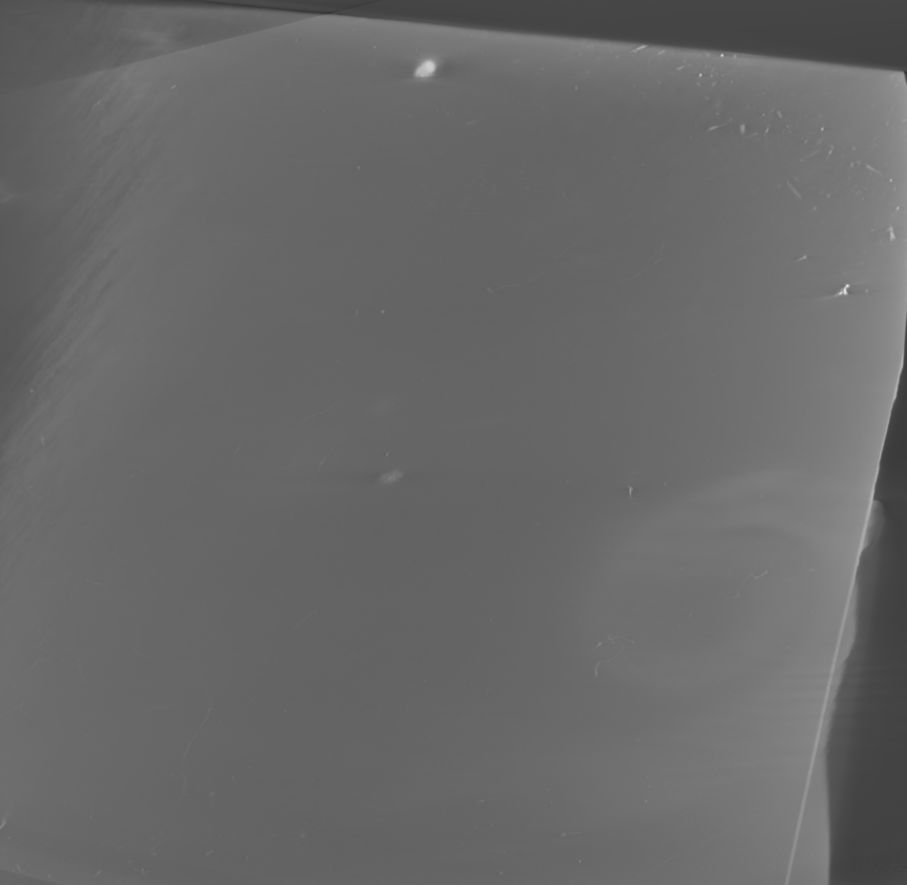

Supplement: Supplemental Information 2 — The used imagestack in the x-z-plane (bmp format) as it was obtained and used in SPIERS. [file peerj-05-3526-s002.zip › front_x-z-plane_036.bmp]

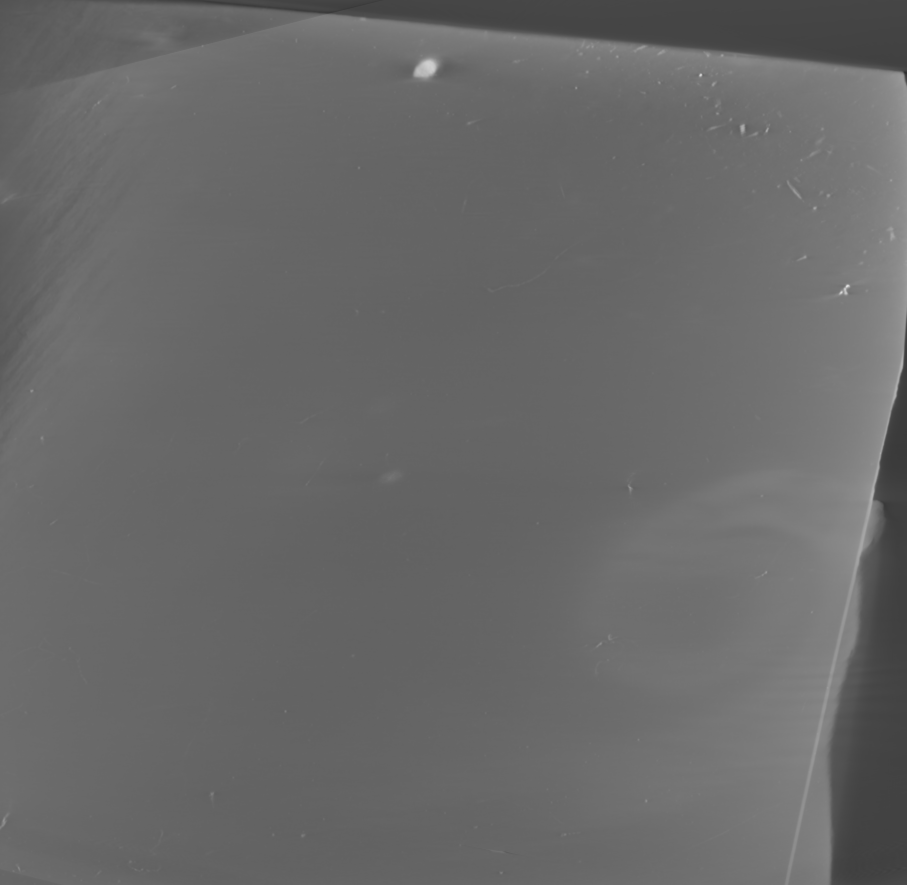

Supplement: Supplemental Information 2 — The used imagestack in the x-z-plane (bmp format) as it was obtained and used in SPIERS. [file peerj-05-3526-s002.zip › front_x-z-plane_037.bmp]

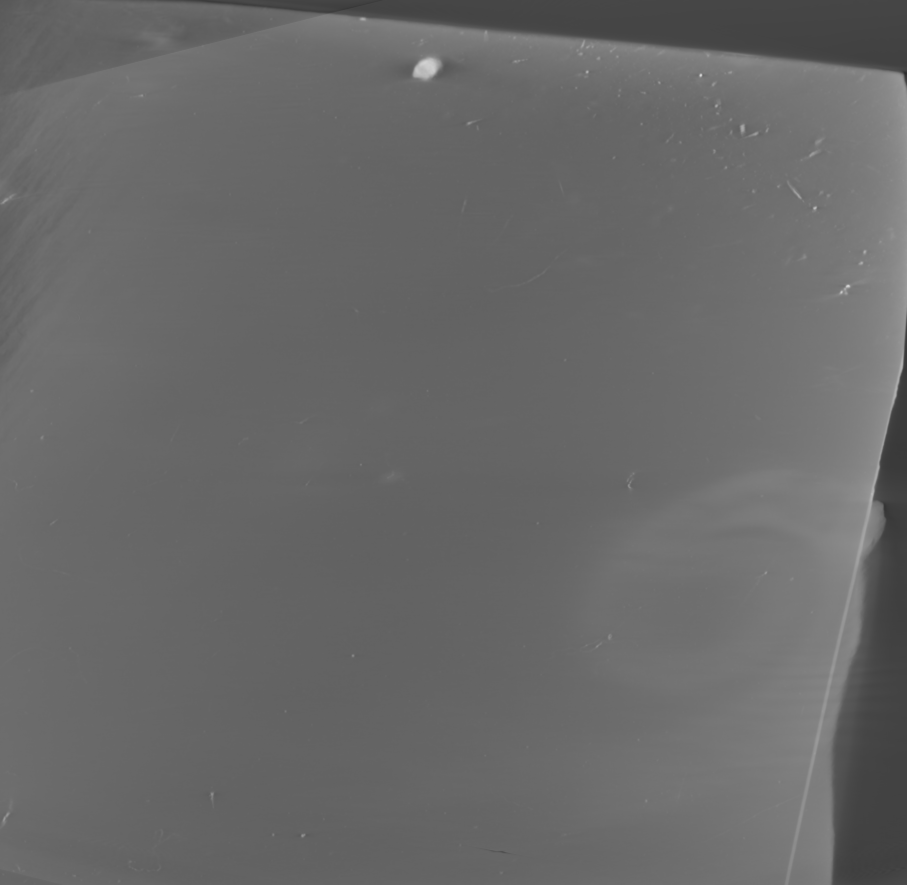

Supplement: Supplemental Information 2 — The used imagestack in the x-z-plane (bmp format) as it was obtained and used in SPIERS. [file peerj-05-3526-s002.zip › front_x-z-plane_038.bmp]

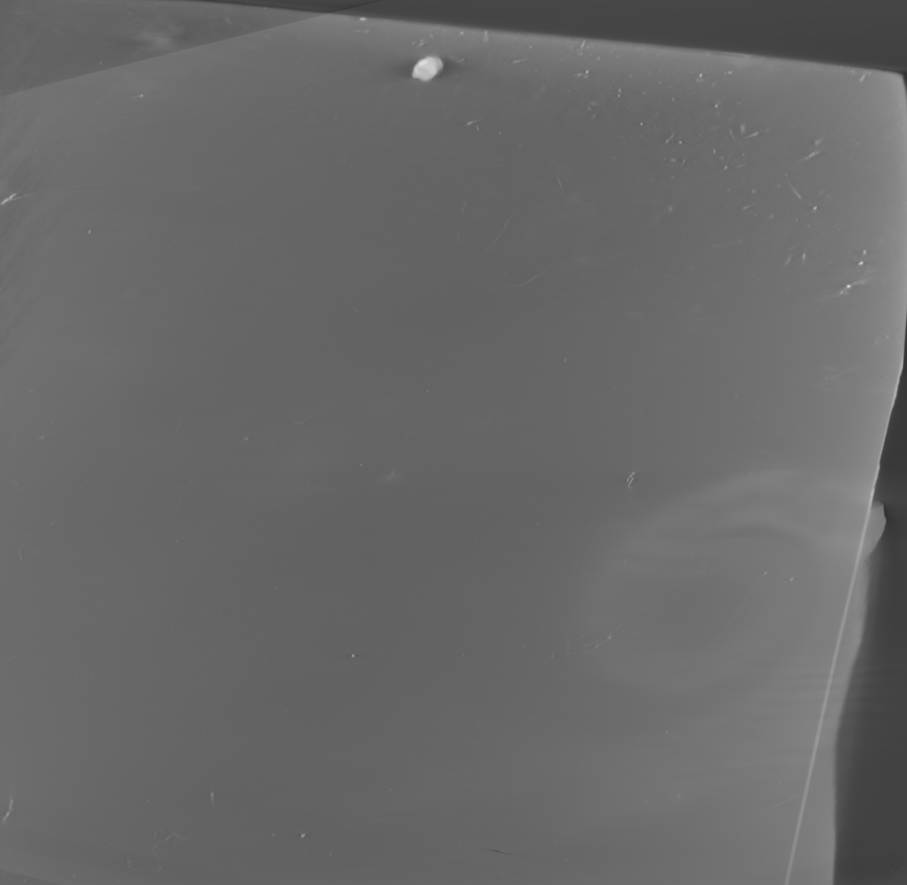

Supplement: Supplemental Information 2 — The used imagestack in the x-z-plane (bmp format) as it was obtained and used in SPIERS. [file peerj-05-3526-s002.zip › front_x-z-plane_039.bmp]

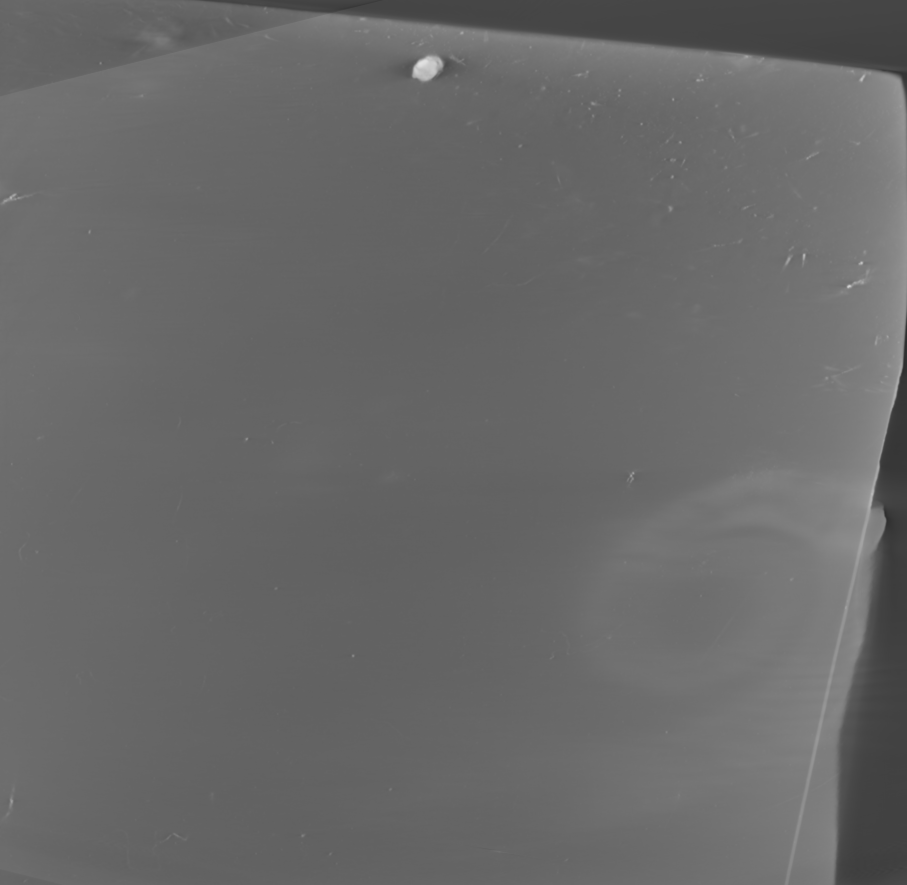

Supplement: Supplemental Information 2 — The used imagestack in the x-z-plane (bmp format) as it was obtained and used in SPIERS. [file peerj-05-3526-s002.zip › front_x-z-plane_040.bmp]

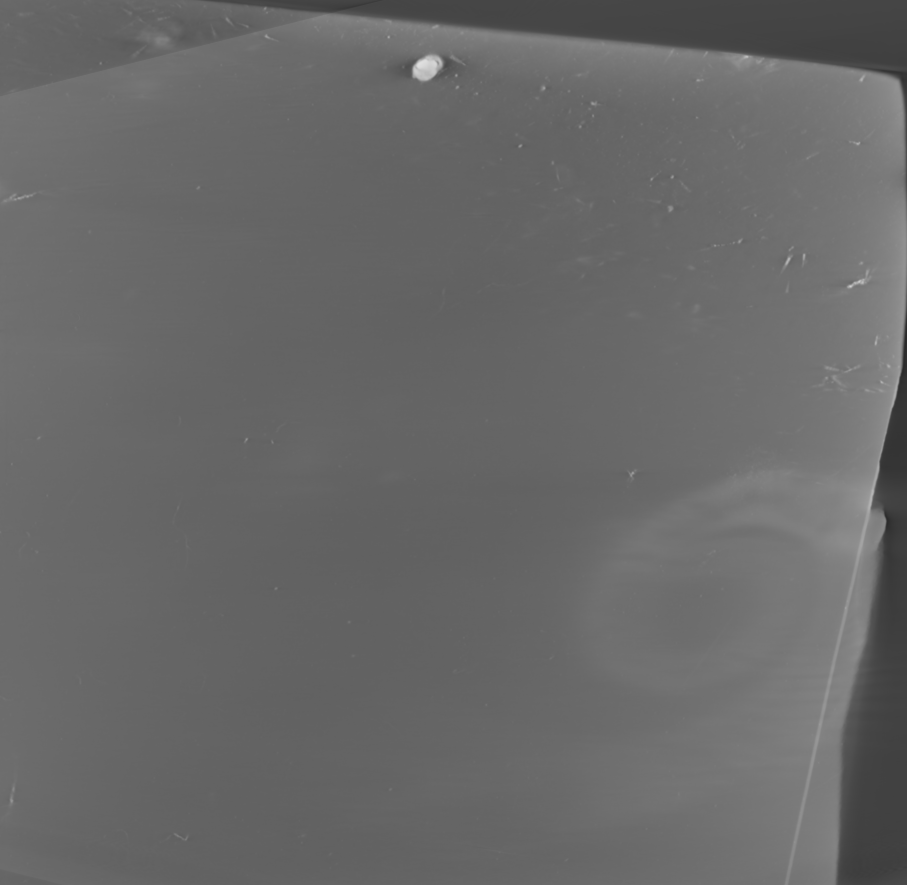

Supplement: Supplemental Information 2 — The used imagestack in the x-z-plane (bmp format) as it was obtained and used in SPIERS. [file peerj-05-3526-s002.zip › front_x-z-plane_041.bmp]

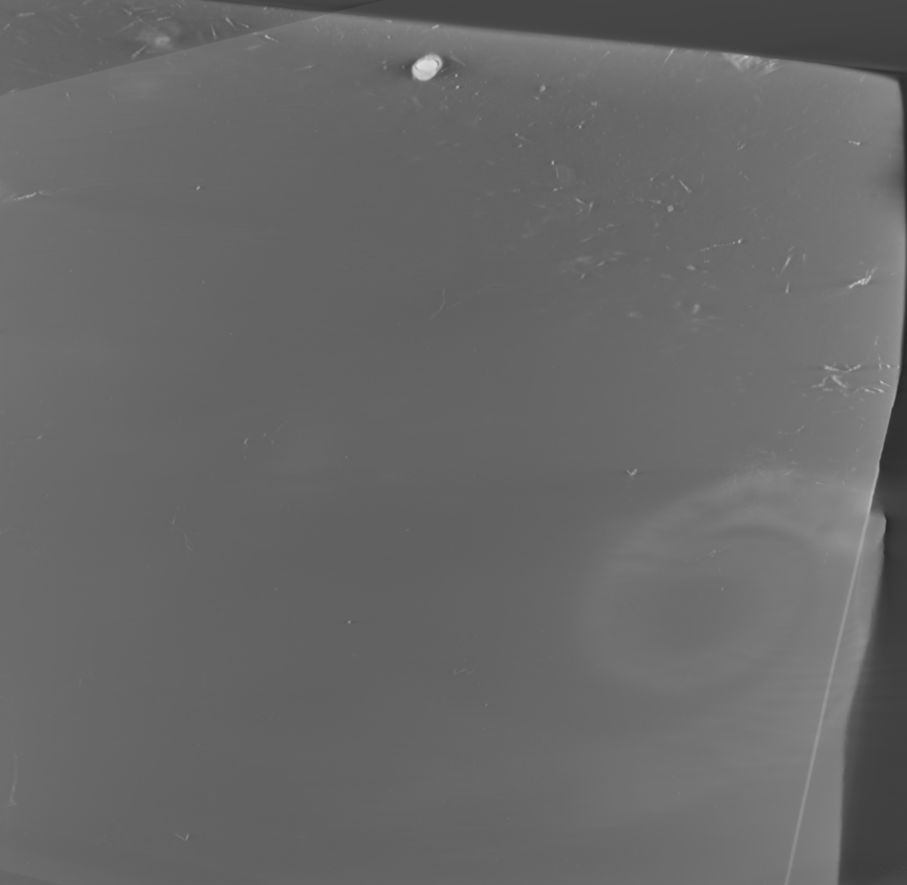

Supplement: Supplemental Information 2 — The used imagestack in the x-z-plane (bmp format) as it was obtained and used in SPIERS. [file peerj-05-3526-s002.zip › front_x-z-plane_042.bmp]

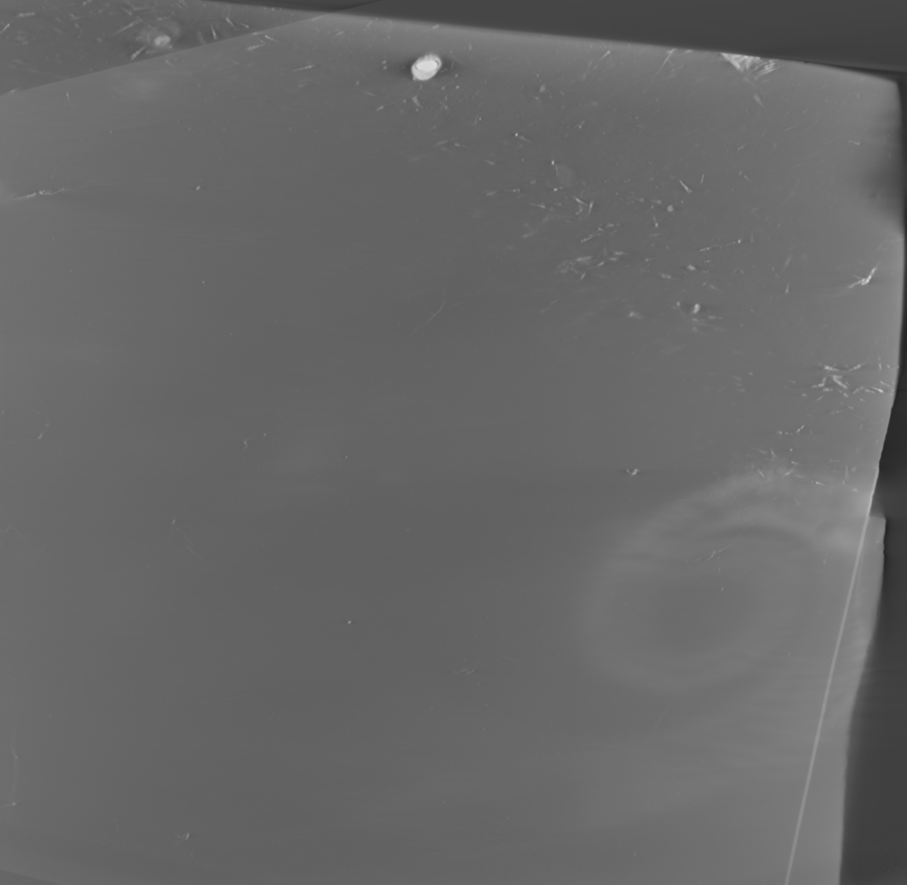

Supplement: Supplemental Information 2 — The used imagestack in the x-z-plane (bmp format) as it was obtained and used in SPIERS. [file peerj-05-3526-s002.zip › front_x-z-plane_043.bmp]

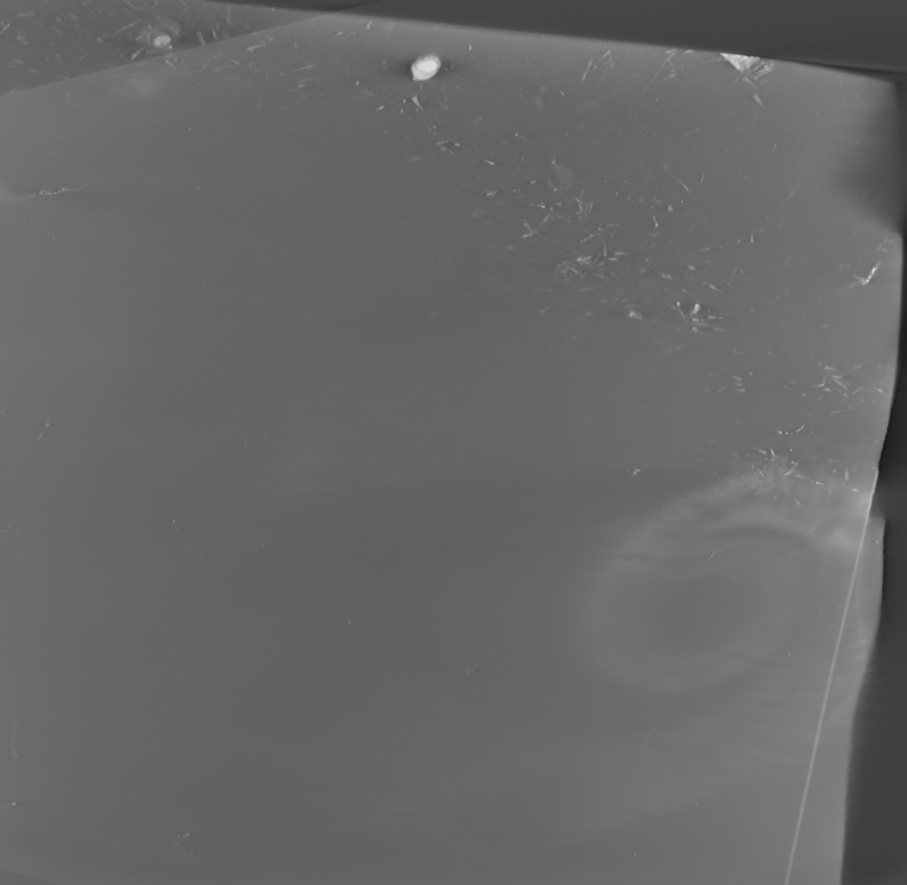

Supplement: Supplemental Information 2 — The used imagestack in the x-z-plane (bmp format) as it was obtained and used in SPIERS. [file peerj-05-3526-s002.zip › front_x-z-plane_044.bmp]

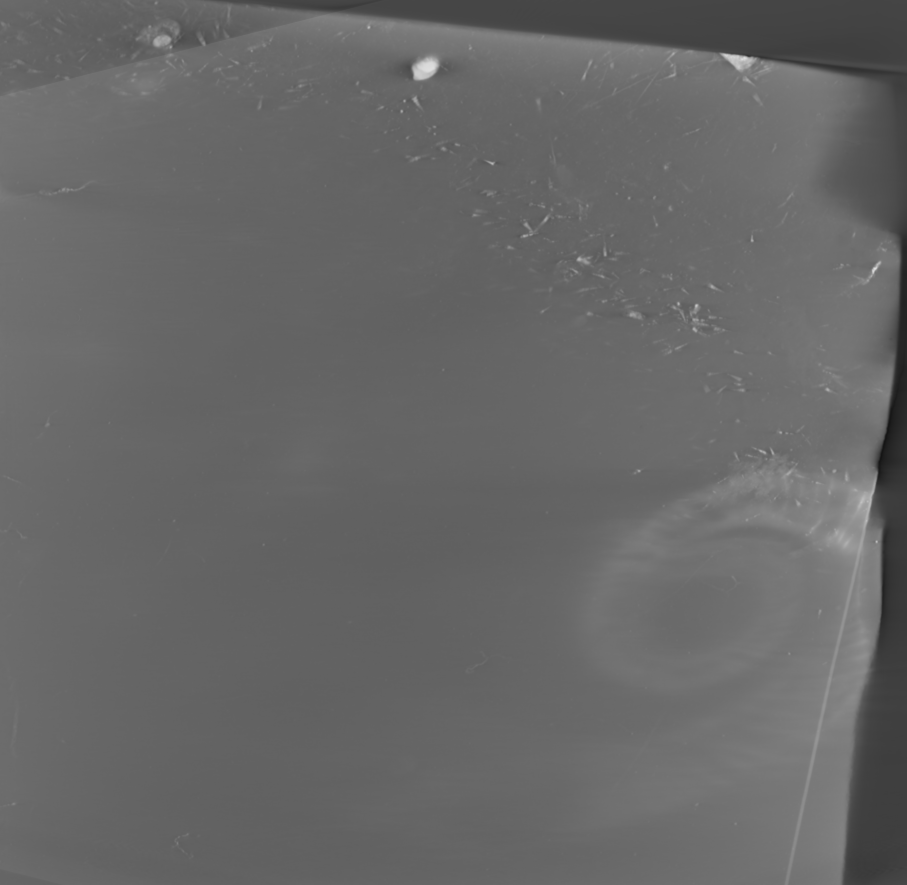

Supplement: Supplemental Information 2 — The used imagestack in the x-z-plane (bmp format) as it was obtained and used in SPIERS. [file peerj-05-3526-s002.zip › front_x-z-plane_045.bmp]

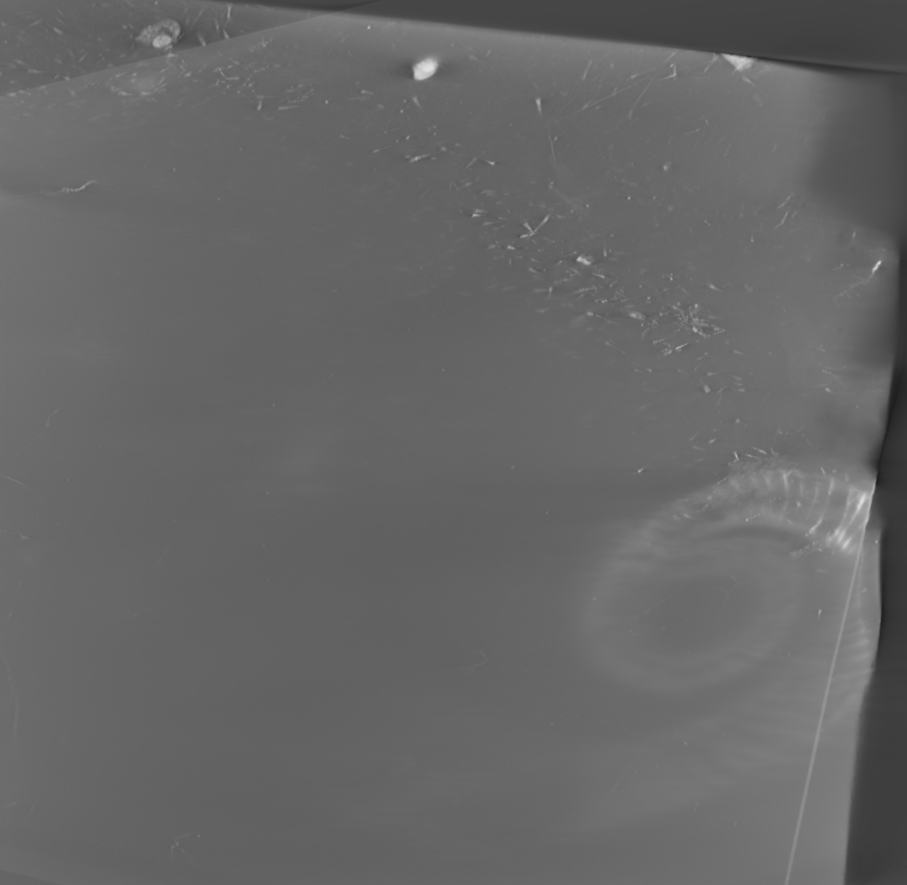

Supplement: Supplemental Information 2 — The used imagestack in the x-z-plane (bmp format) as it was obtained and used in SPIERS. [file peerj-05-3526-s002.zip › front_x-z-plane_046.bmp]

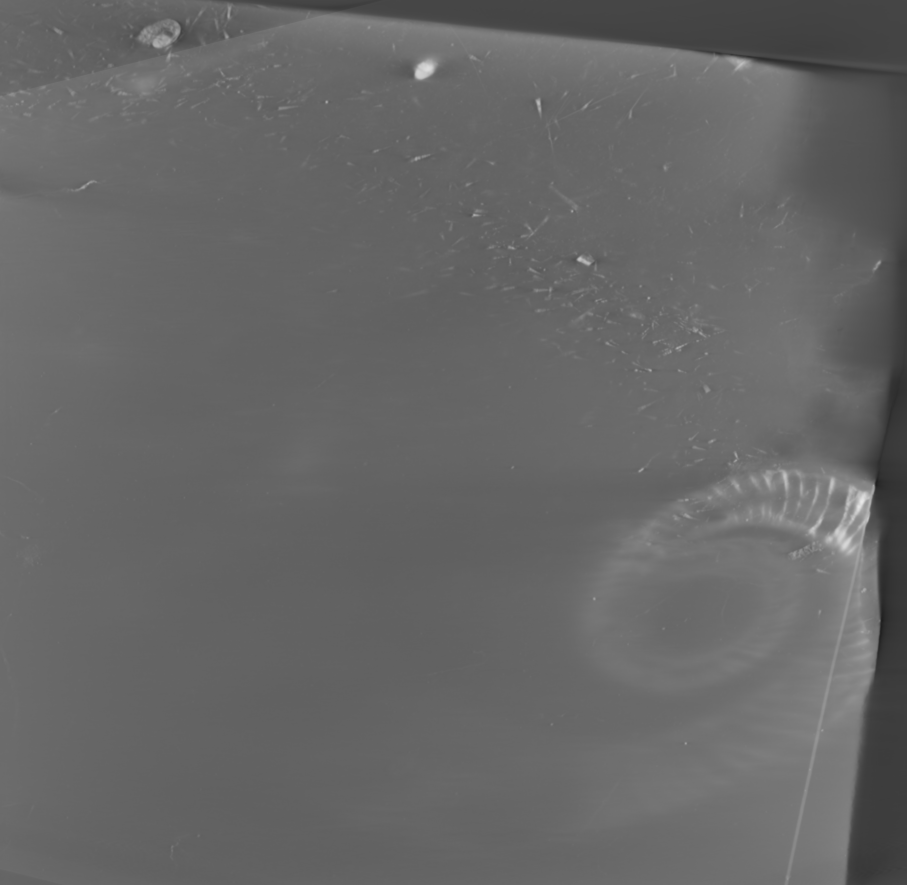

Supplement: Supplemental Information 2 — The used imagestack in the x-z-plane (bmp format) as it was obtained and used in SPIERS. [file peerj-05-3526-s002.zip › front_x-z-plane_047.bmp]

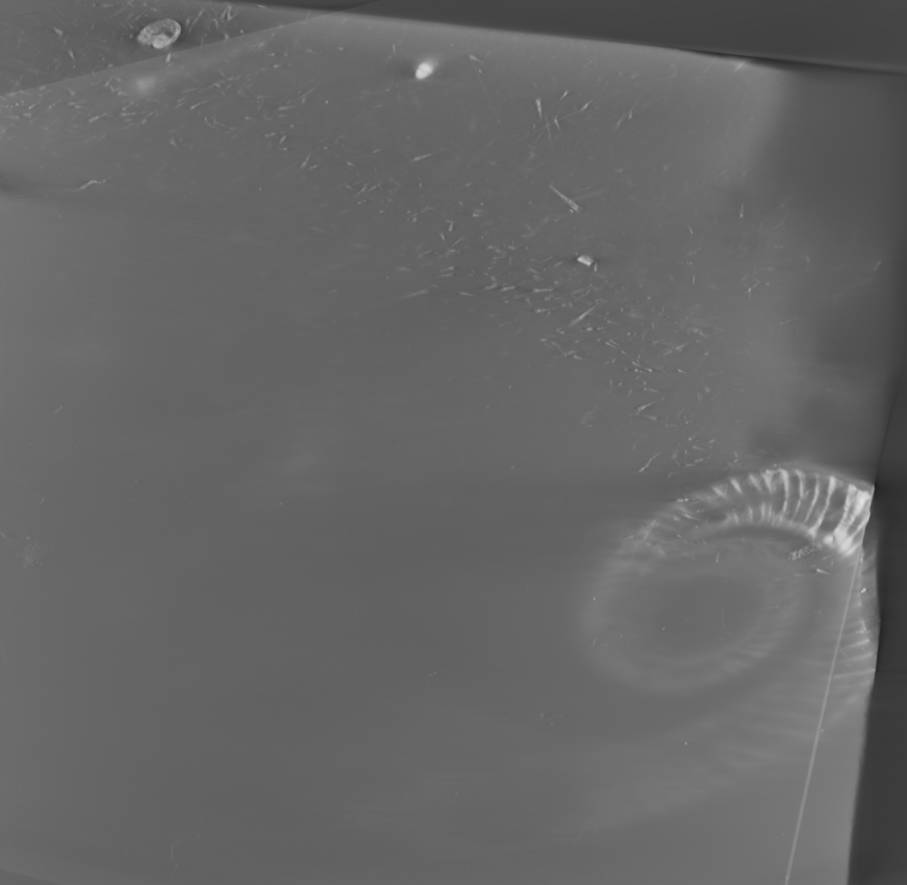

Supplement: Supplemental Information 2 — The used imagestack in the x-z-plane (bmp format) as it was obtained and used in SPIERS. [file peerj-05-3526-s002.zip › front_x-z-plane_048.bmp]

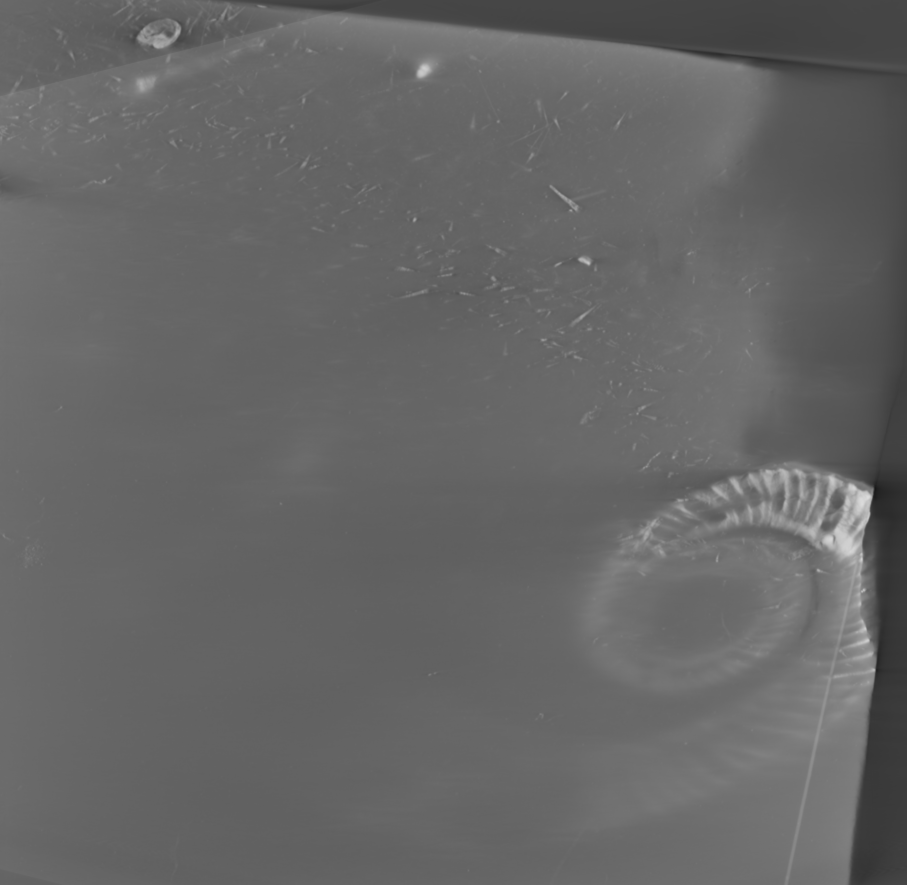

Supplement: Supplemental Information 2 — The used imagestack in the x-z-plane (bmp format) as it was obtained and used in SPIERS. [file peerj-05-3526-s002.zip › front_x-z-plane_049.bmp]

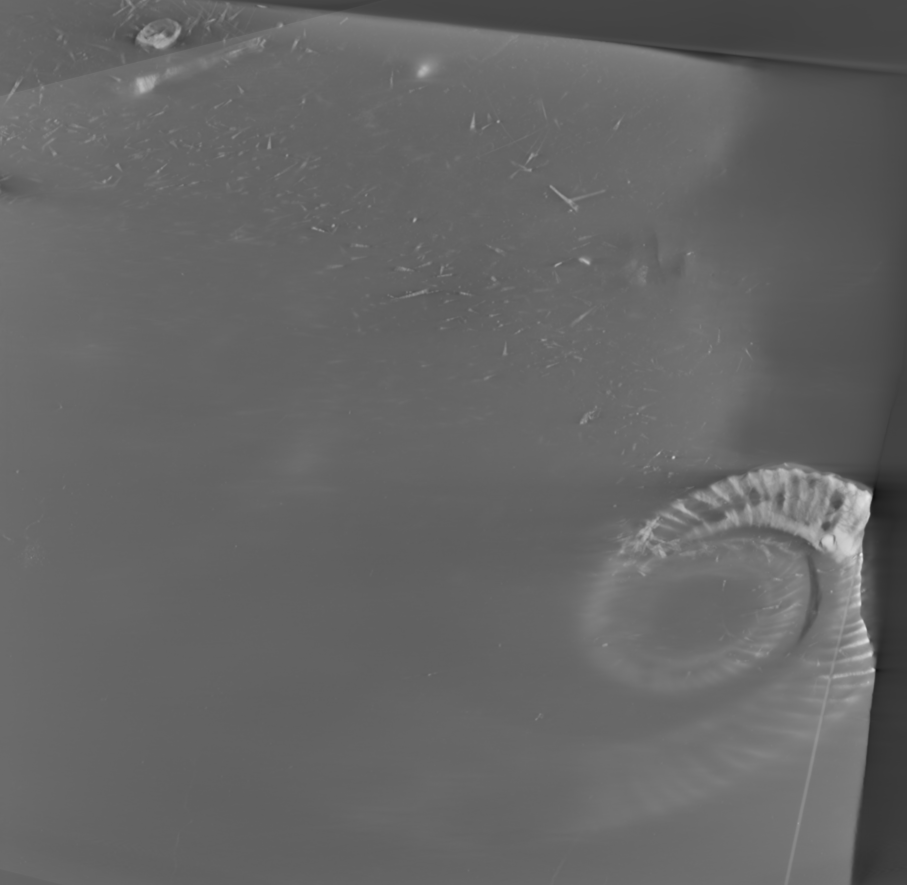

Supplement: Supplemental Information 2 — The used imagestack in the x-z-plane (bmp format) as it was obtained and used in SPIERS. [file peerj-05-3526-s002.zip › front_x-z-plane_050.bmp]

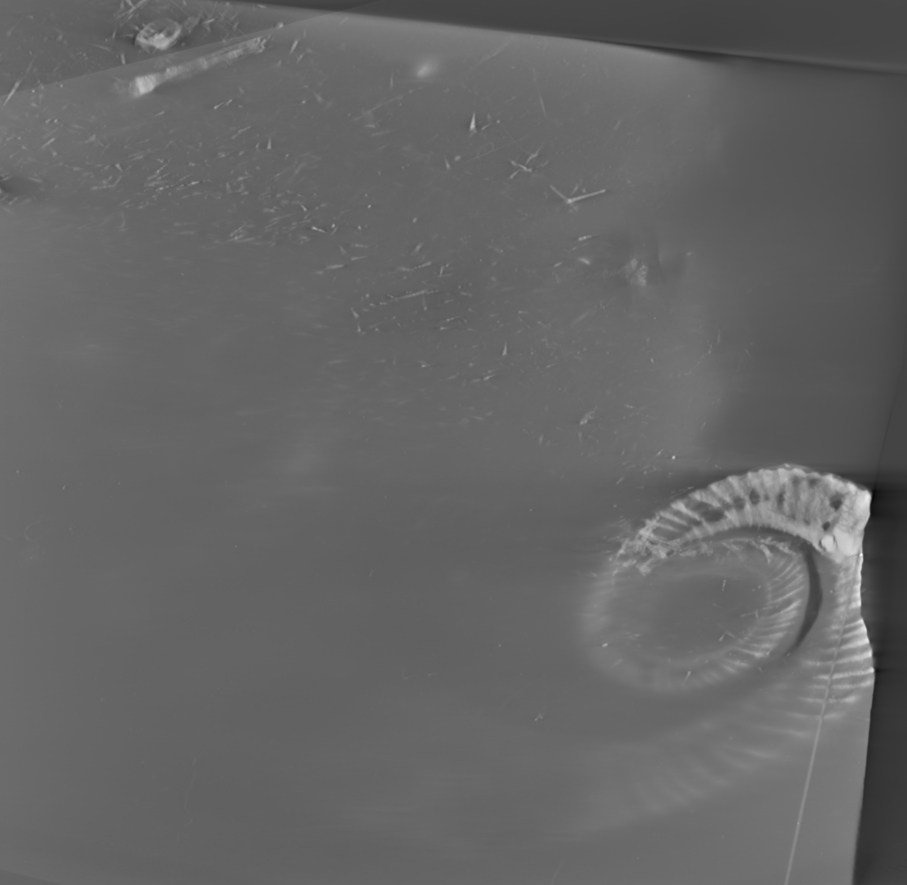

Supplement: Supplemental Information 2 — The used imagestack in the x-z-plane (bmp format) as it was obtained and used in SPIERS. [file peerj-05-3526-s002.zip › front_x-z-plane_051.bmp]

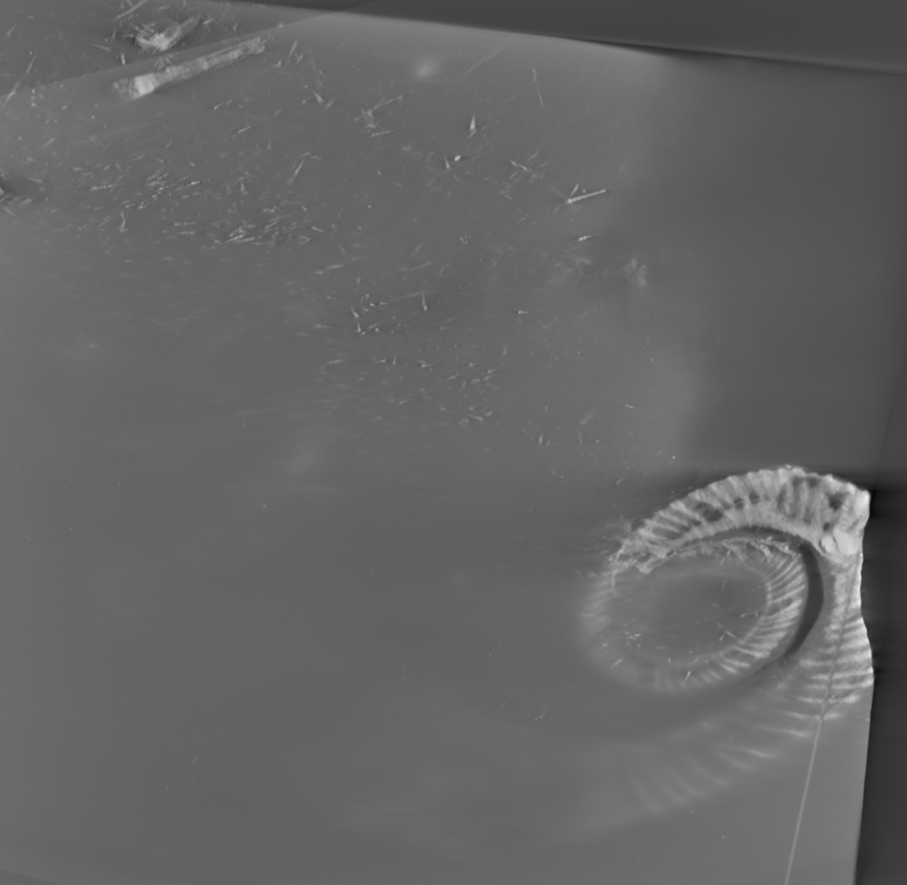

Supplement: Supplemental Information 2 — The used imagestack in the x-z-plane (bmp format) as it was obtained and used in SPIERS. [file peerj-05-3526-s002.zip › front_x-z-plane_052.bmp]

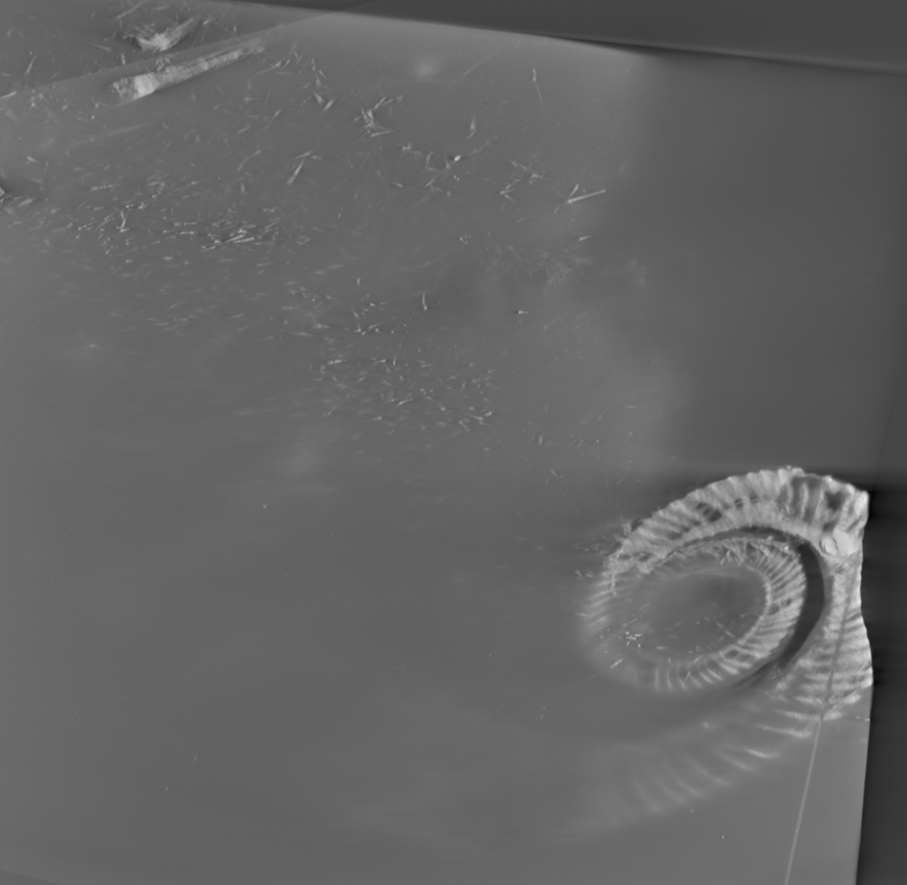

Supplement: Supplemental Information 2 — The used imagestack in the x-z-plane (bmp format) as it was obtained and used in SPIERS. [file peerj-05-3526-s002.zip › front_x-z-plane_053.bmp]

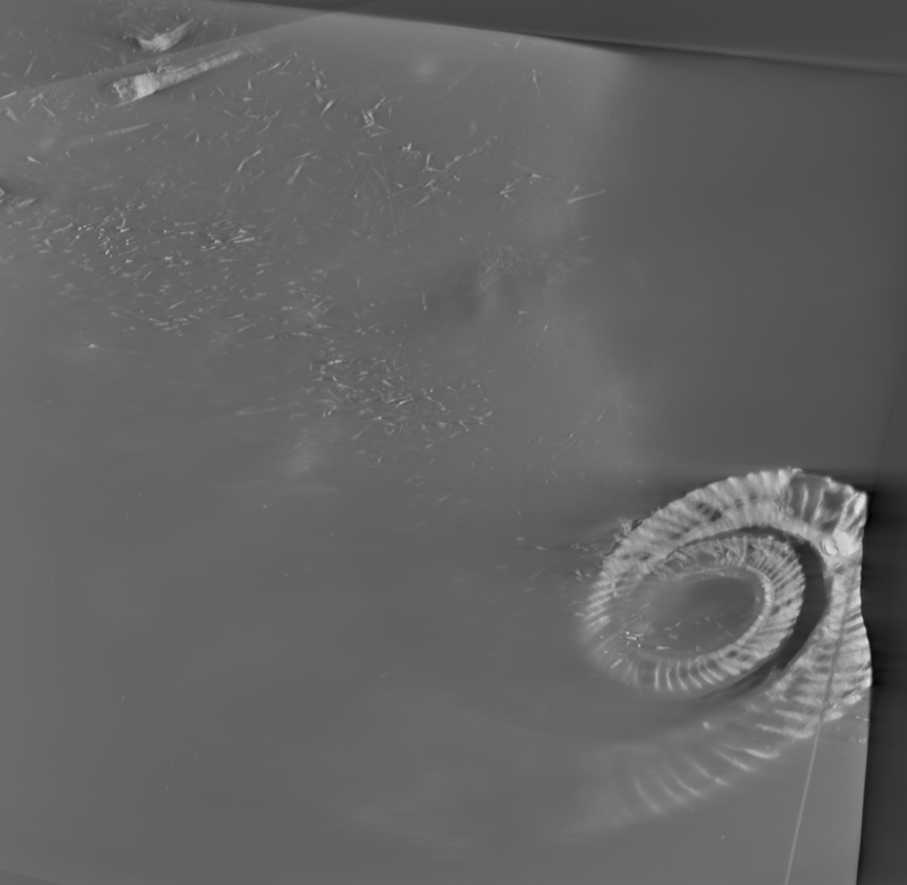

Supplement: Supplemental Information 2 — The used imagestack in the x-z-plane (bmp format) as it was obtained and used in SPIERS. [file peerj-05-3526-s002.zip › front_x-z-plane_054.bmp]

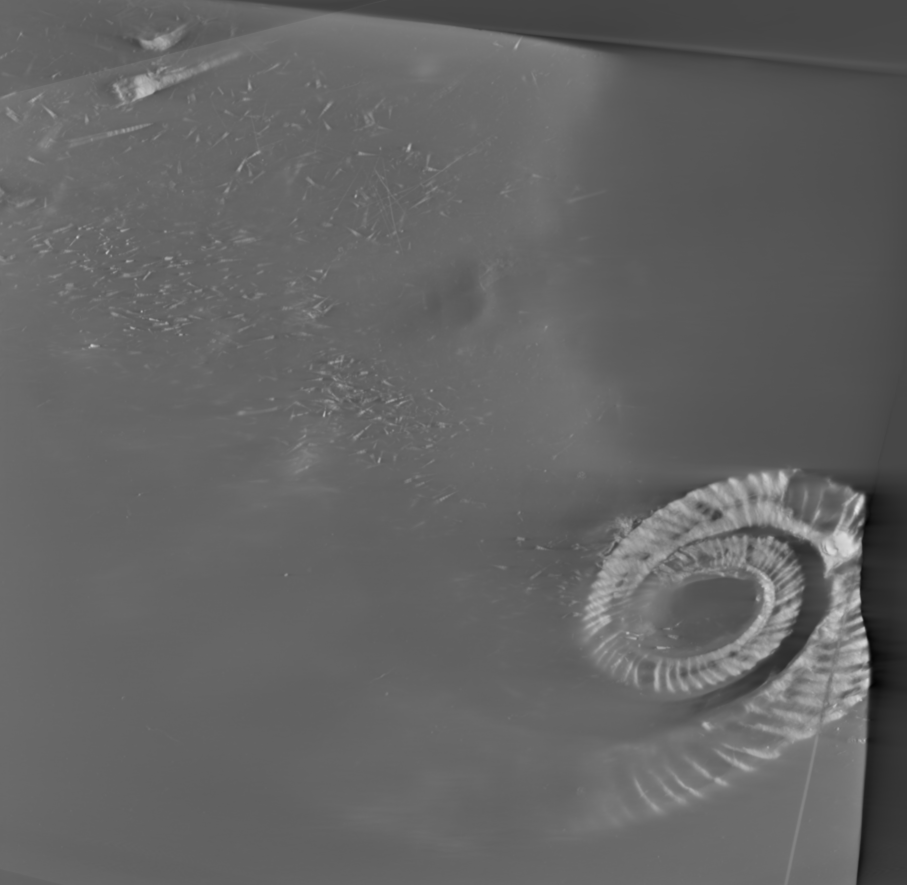

Supplement: Supplemental Information 2 — The used imagestack in the x-z-plane (bmp format) as it was obtained and used in SPIERS. [file peerj-05-3526-s002.zip › front_x-z-plane_055.bmp]

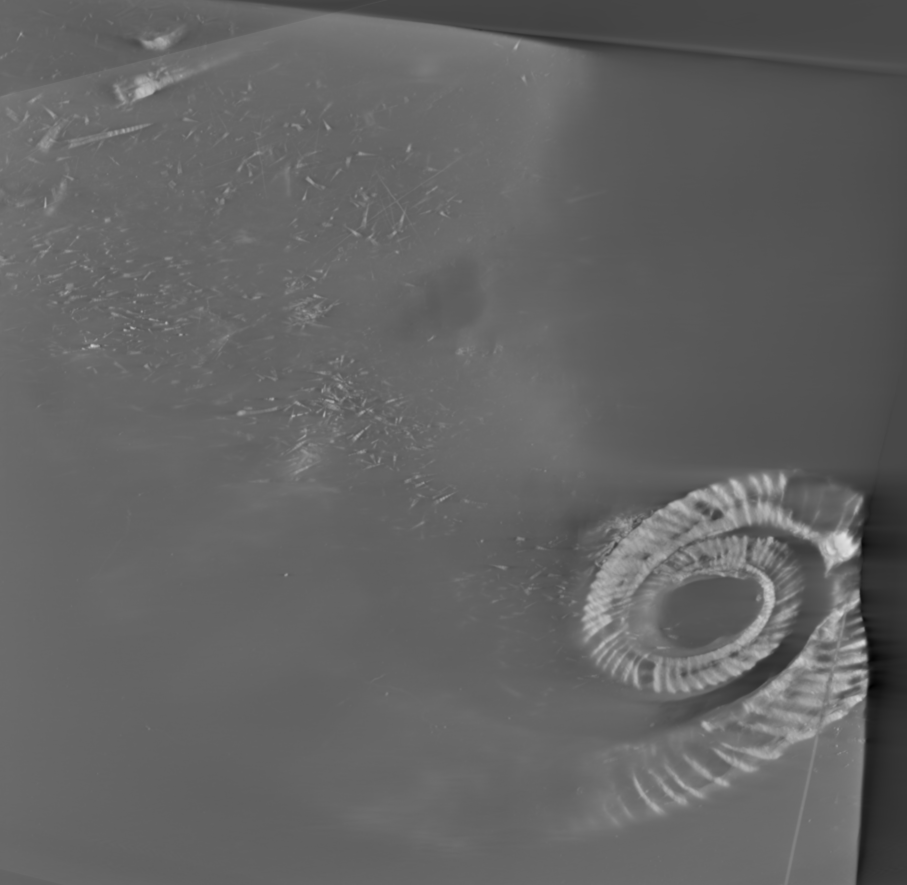

Supplement: Supplemental Information 2 — The used imagestack in the x-z-plane (bmp format) as it was obtained and used in SPIERS. [file peerj-05-3526-s002.zip › front_x-z-plane_056.bmp]

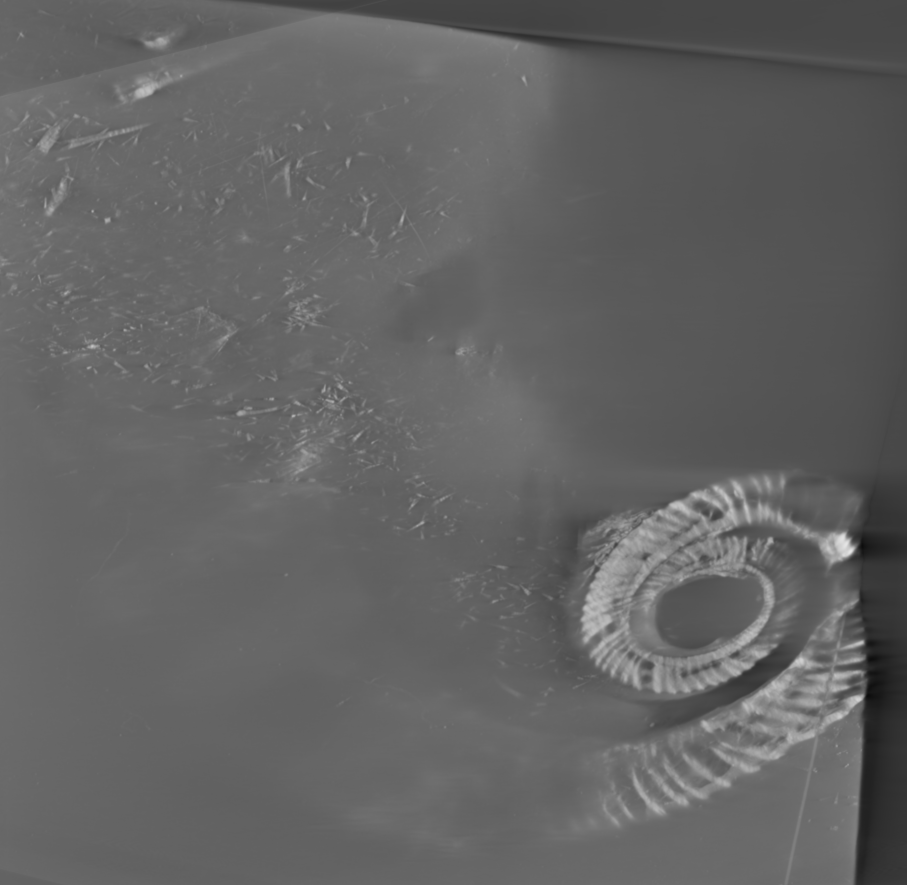

Supplement: Supplemental Information 2 — The used imagestack in the x-z-plane (bmp format) as it was obtained and used in SPIERS. [file peerj-05-3526-s002.zip › front_x-z-plane_057.bmp]

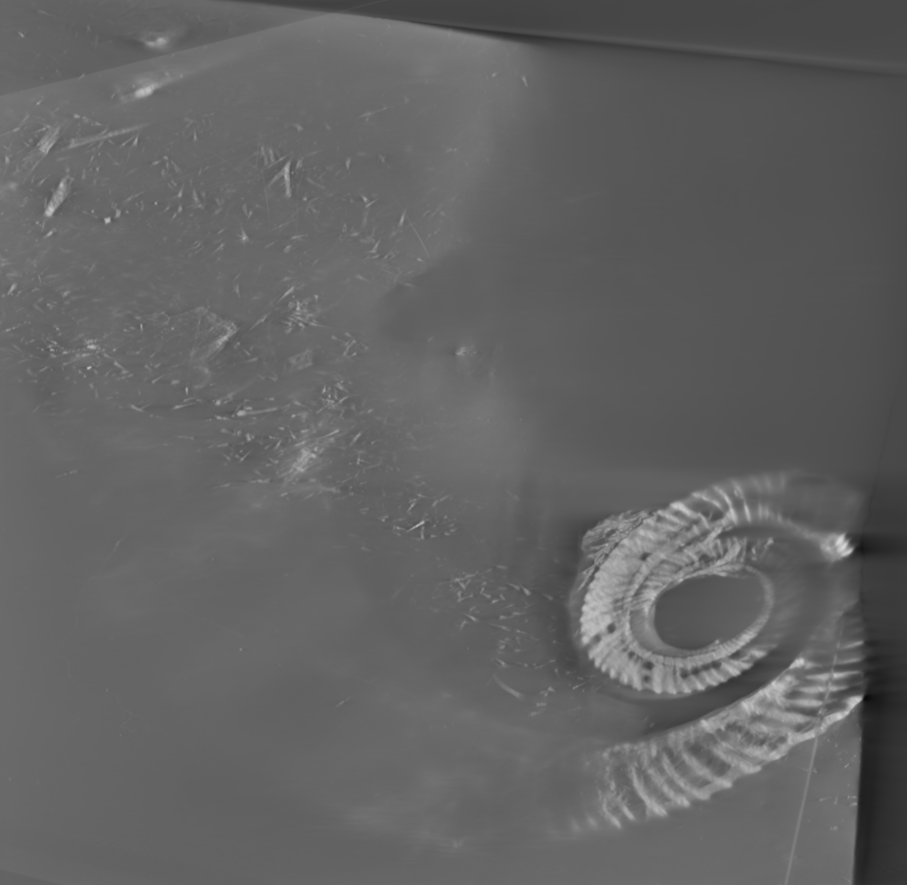

Supplement: Supplemental Information 2 — The used imagestack in the x-z-plane (bmp format) as it was obtained and used in SPIERS. [file peerj-05-3526-s002.zip › front_x-z-plane_058.bmp]

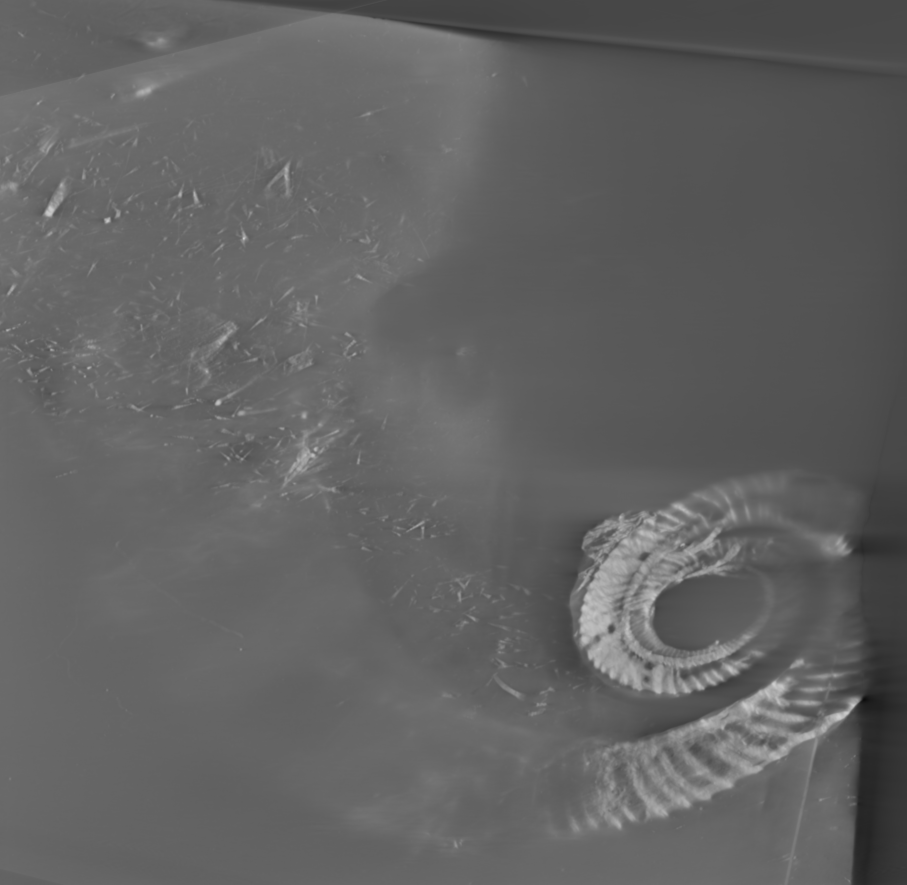

Supplement: Supplemental Information 2 — The used imagestack in the x-z-plane (bmp format) as it was obtained and used in SPIERS. [file peerj-05-3526-s002.zip › front_x-z-plane_059.bmp]

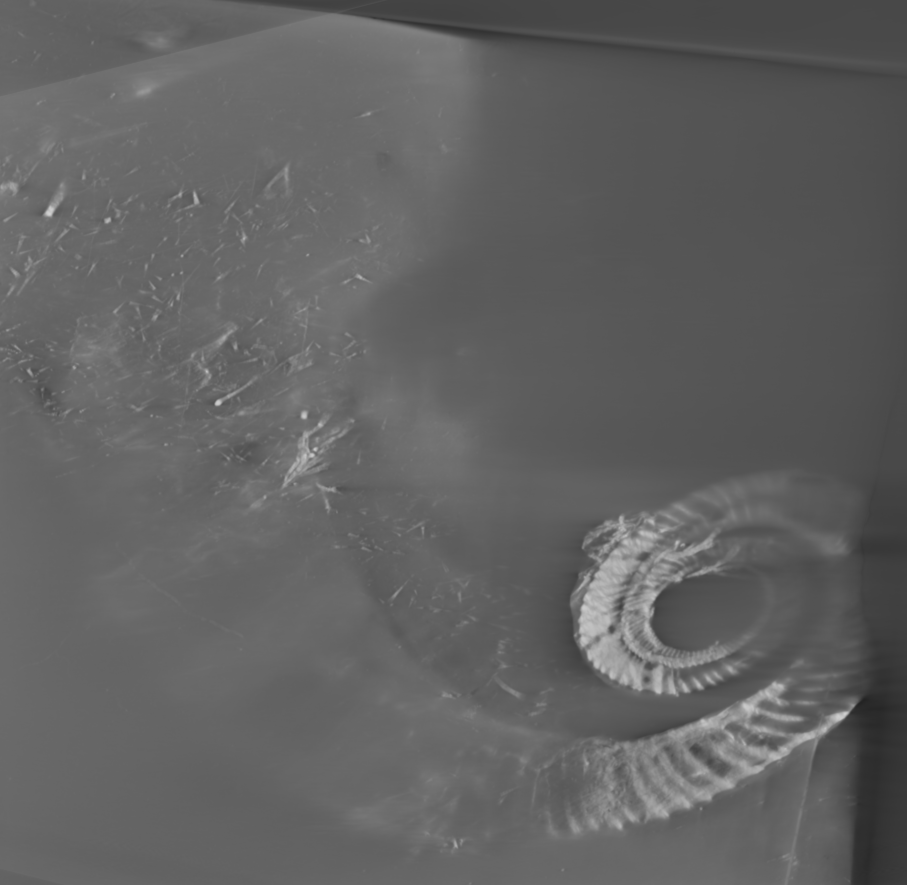

Supplement: Supplemental Information 2 — The used imagestack in the x-z-plane (bmp format) as it was obtained and used in SPIERS. [file peerj-05-3526-s002.zip › front_x-z-plane_060.bmp]

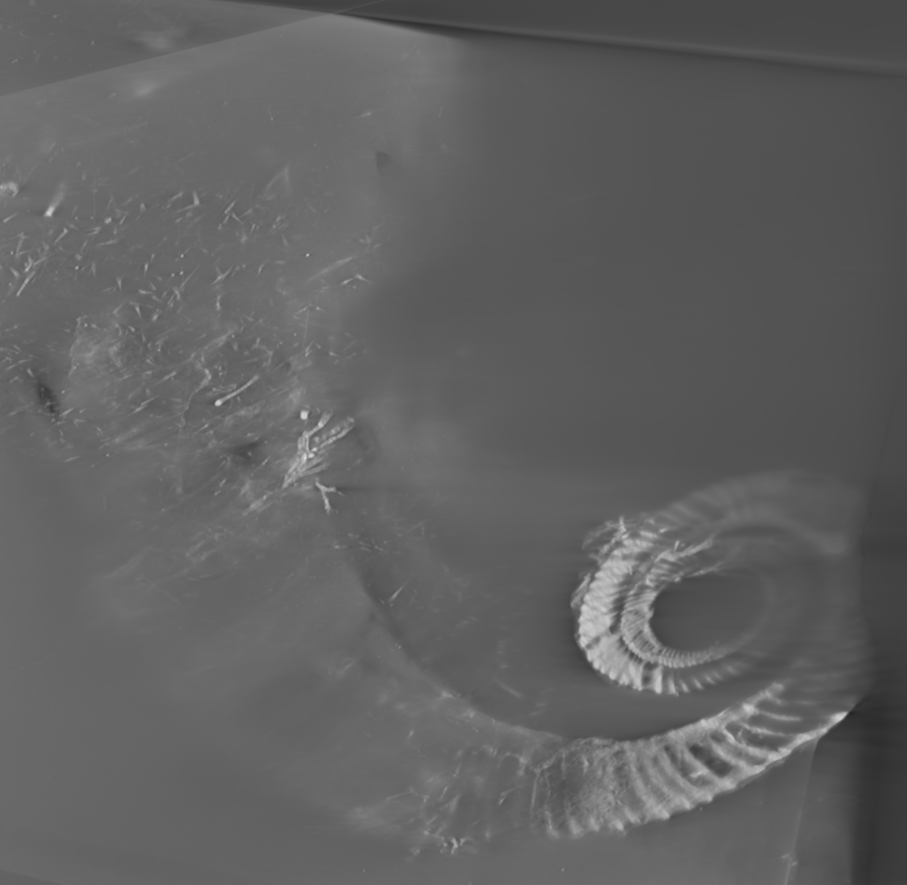

Supplement: Supplemental Information 2 — The used imagestack in the x-z-plane (bmp format) as it was obtained and used in SPIERS. [file peerj-05-3526-s002.zip › front_x-z-plane_061.bmp]

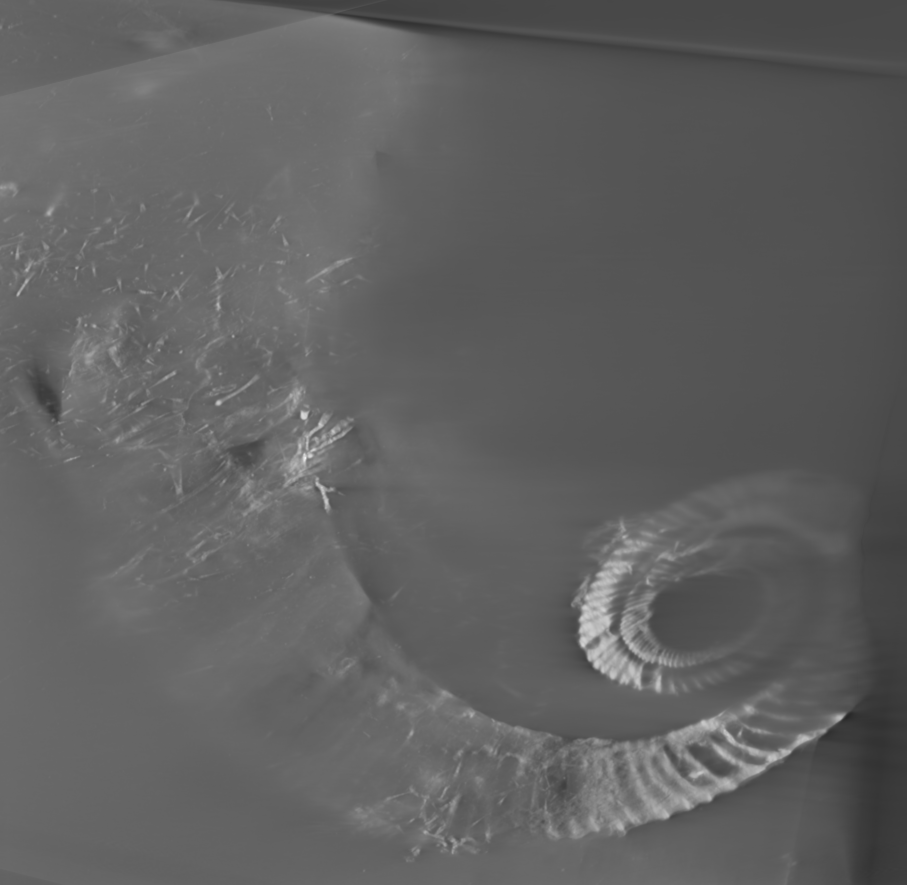

Supplement: Supplemental Information 2 — The used imagestack in the x-z-plane (bmp format) as it was obtained and used in SPIERS. [file peerj-05-3526-s002.zip › front_x-z-plane_062.bmp]

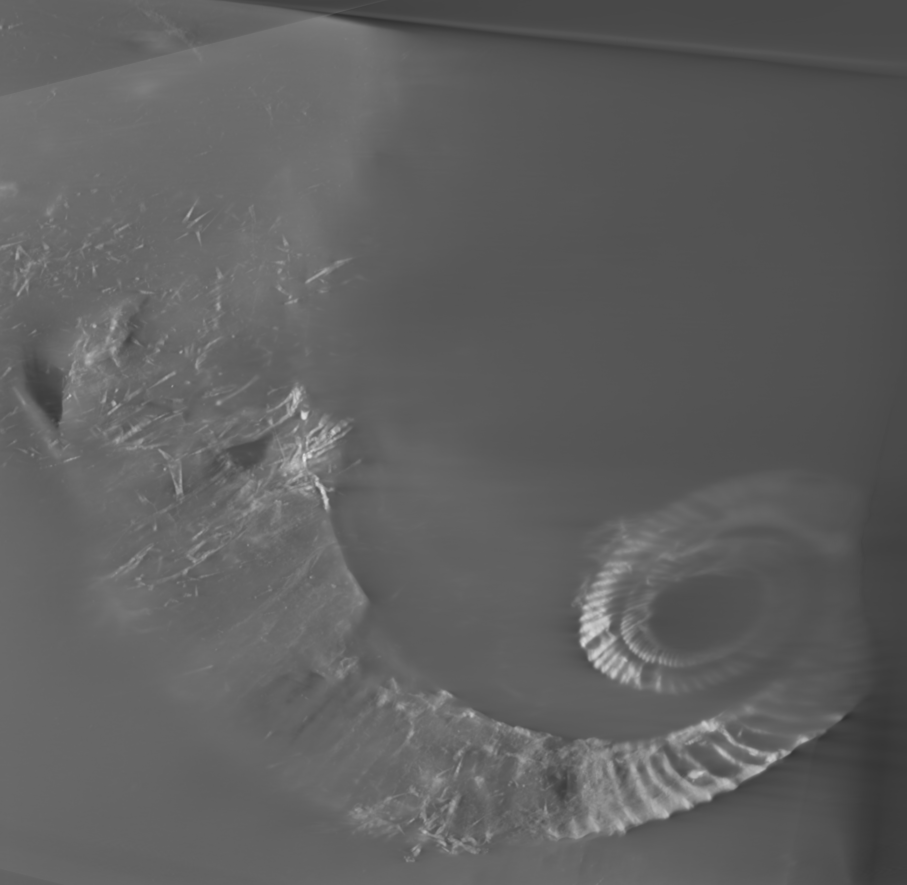

Supplement: Supplemental Information 2 — The used imagestack in the x-z-plane (bmp format) as it was obtained and used in SPIERS. [file peerj-05-3526-s002.zip › front_x-z-plane_063.bmp]

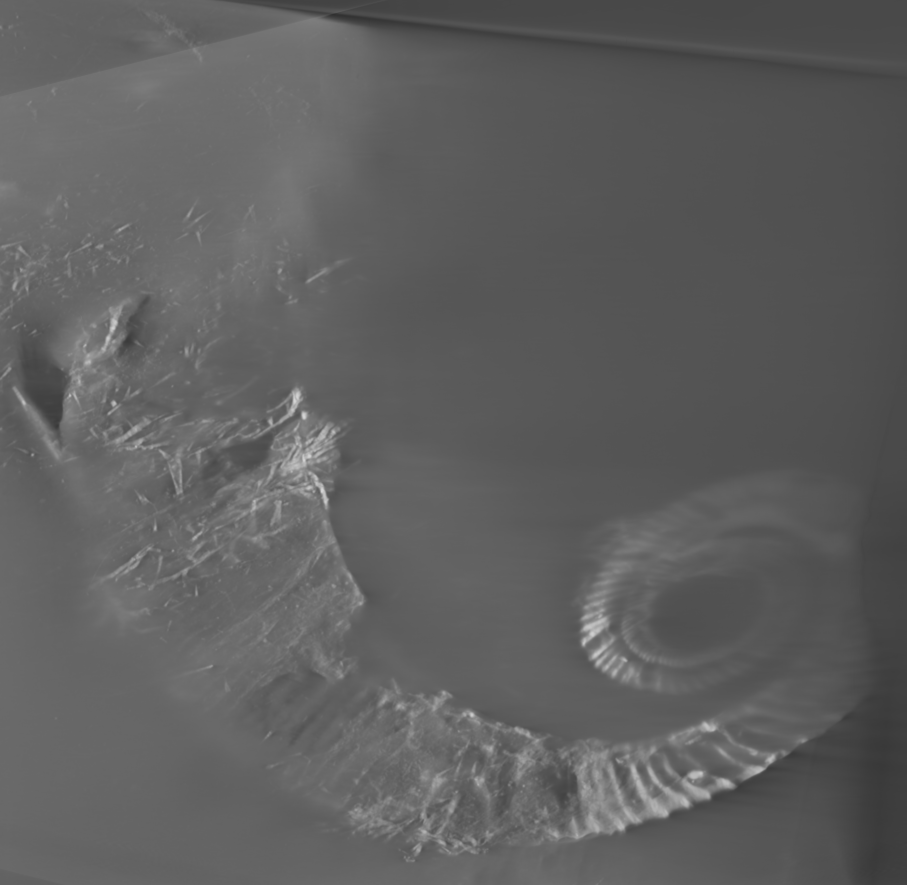

Supplement: Supplemental Information 2 — The used imagestack in the x-z-plane (bmp format) as it was obtained and used in SPIERS. [file peerj-05-3526-s002.zip › front_x-z-plane_064.bmp]

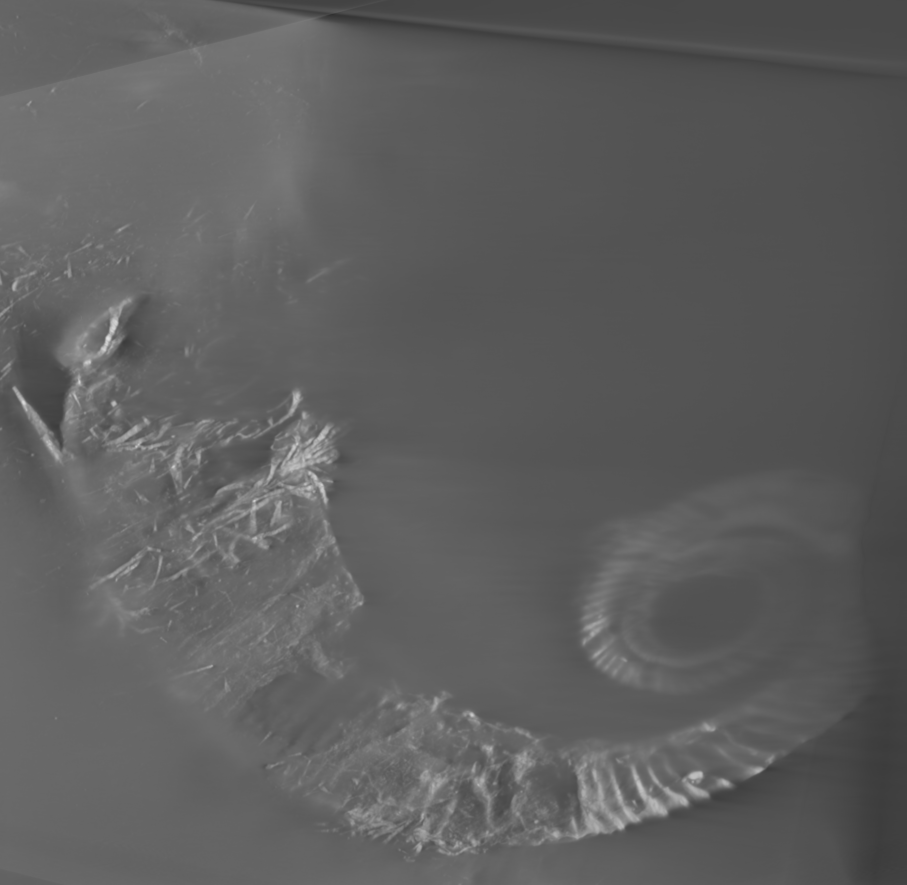

Supplement: Supplemental Information 2 — The used imagestack in the x-z-plane (bmp format) as it was obtained and used in SPIERS. [file peerj-05-3526-s002.zip › front_x-z-plane_065.bmp]

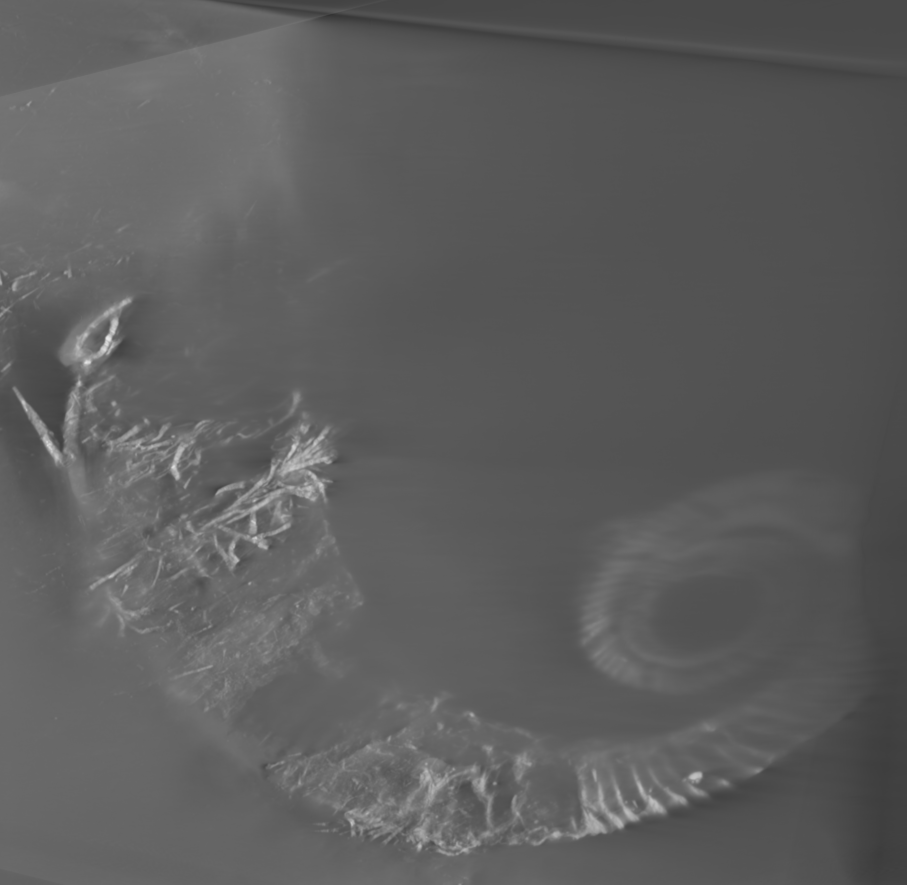

Supplement: Supplemental Information 2 — The used imagestack in the x-z-plane (bmp format) as it was obtained and used in SPIERS. [file peerj-05-3526-s002.zip › front_x-z-plane_066.bmp]

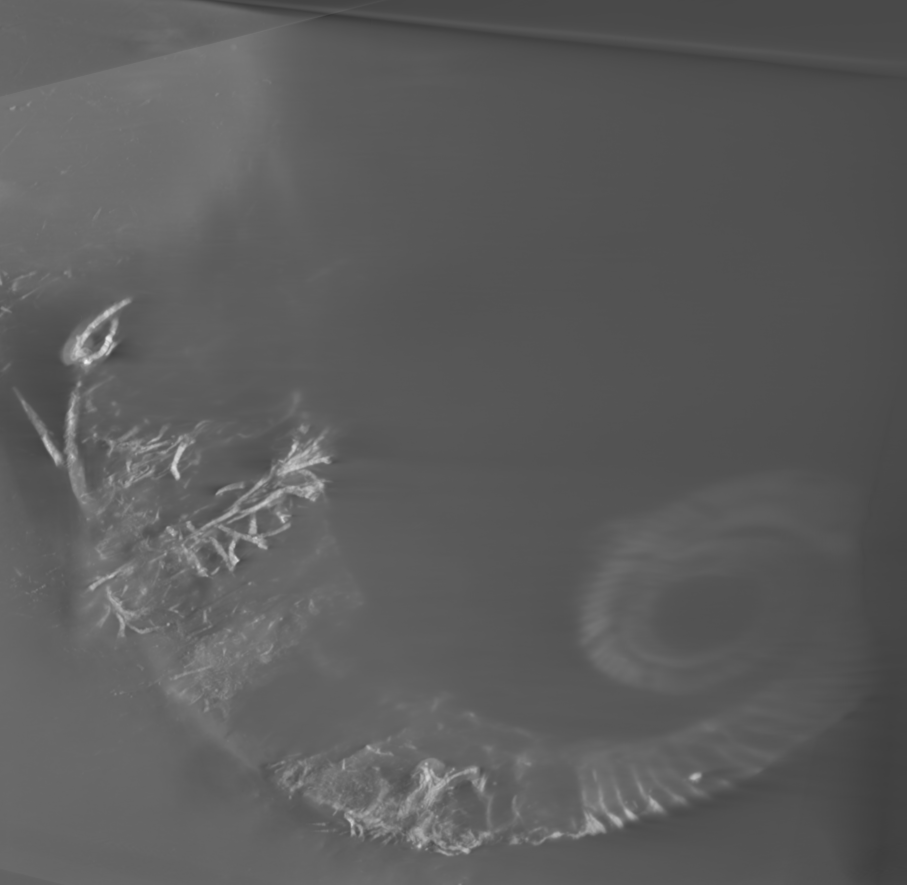

Supplement: Supplemental Information 2 — The used imagestack in the x-z-plane (bmp format) as it was obtained and used in SPIERS. [file peerj-05-3526-s002.zip › front_x-z-plane_067.bmp]

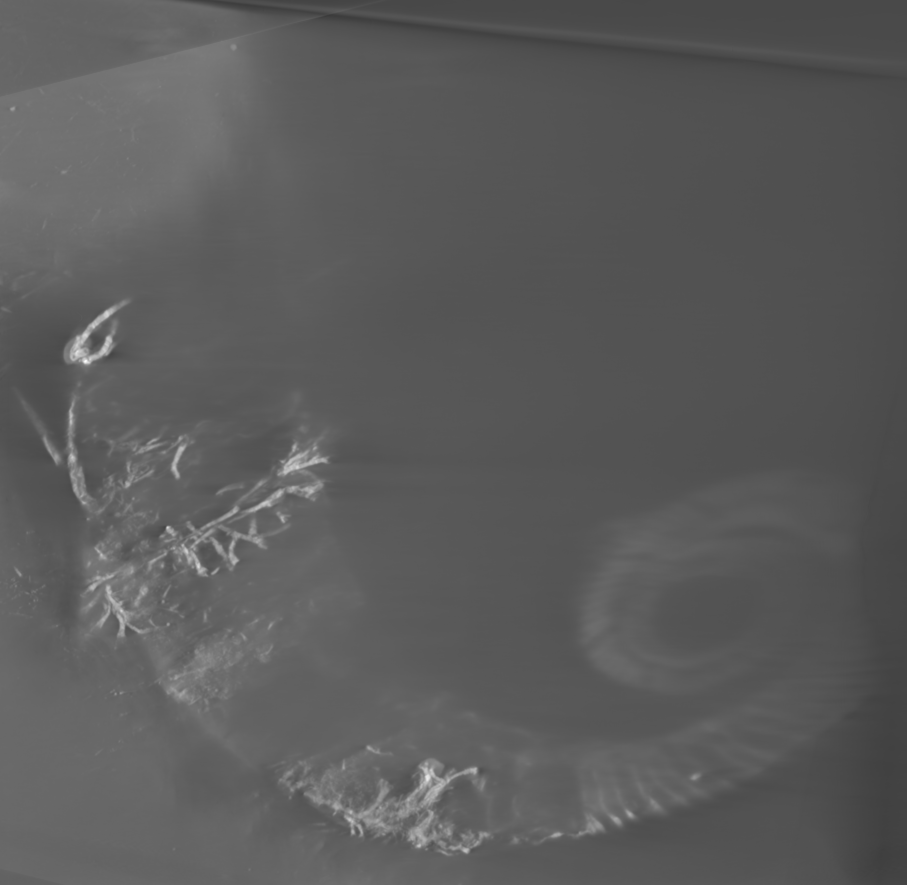

Supplement: Supplemental Information 2 — The used imagestack in the x-z-plane (bmp format) as it was obtained and used in SPIERS. [file peerj-05-3526-s002.zip › front_x-z-plane_068.bmp]

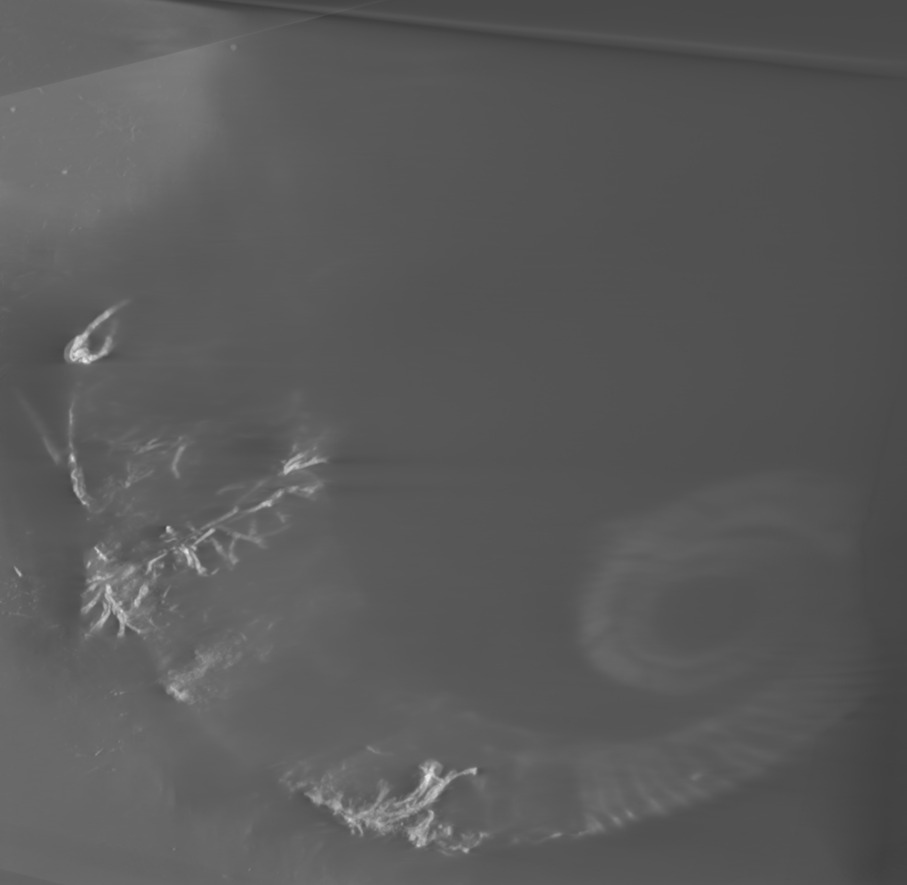

Supplement: Supplemental Information 2 — The used imagestack in the x-z-plane (bmp format) as it was obtained and used in SPIERS. [file peerj-05-3526-s002.zip › front_x-z-plane_069.bmp]

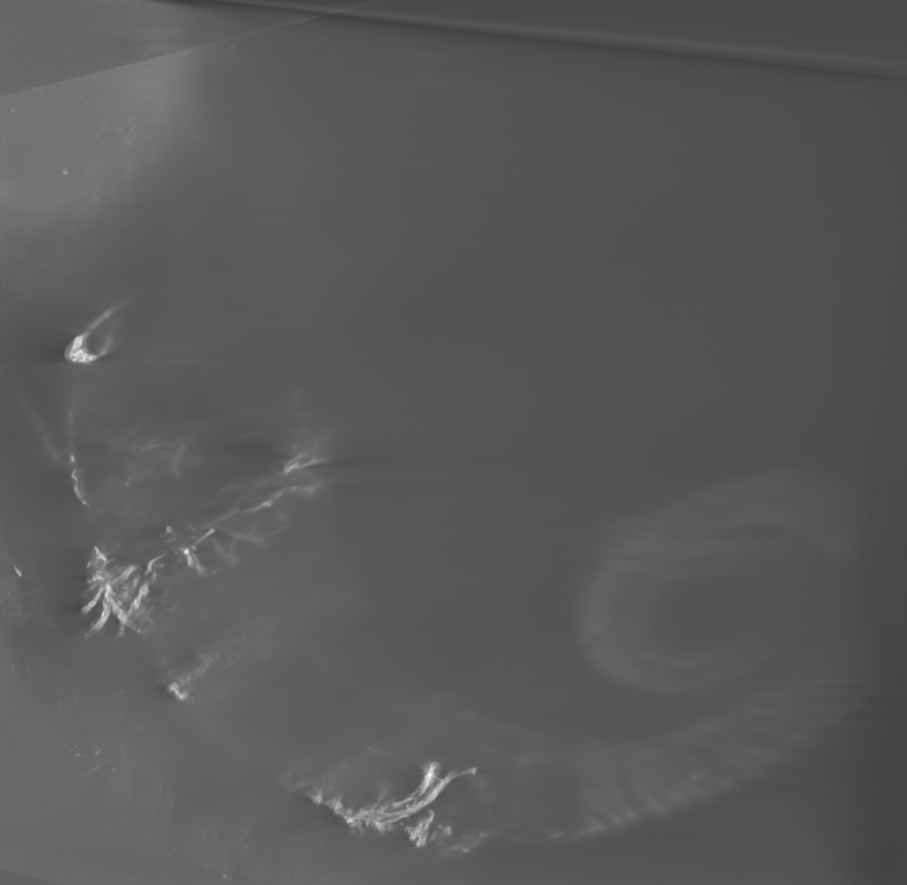

Supplement: Supplemental Information 2 — The used imagestack in the x-z-plane (bmp format) as it was obtained and used in SPIERS. [file peerj-05-3526-s002.zip › front_x-z-plane_070.bmp]

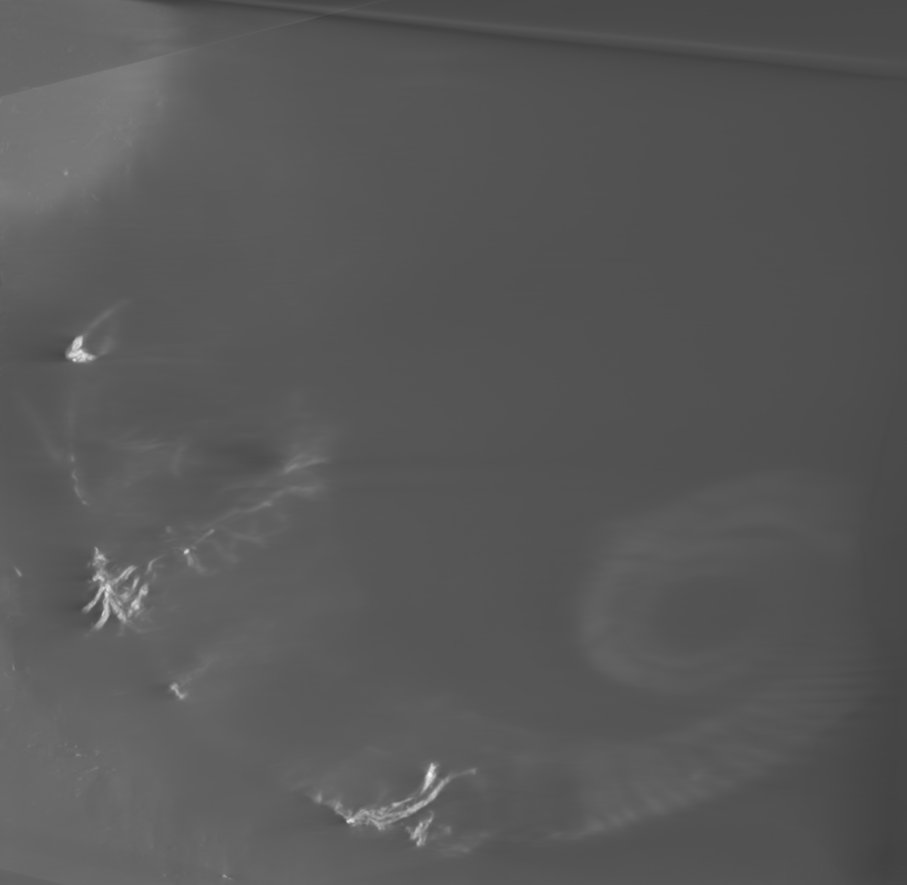

Supplement: Supplemental Information 2 — The used imagestack in the x-z-plane (bmp format) as it was obtained and used in SPIERS. [file peerj-05-3526-s002.zip › front_x-z-plane_071.bmp]

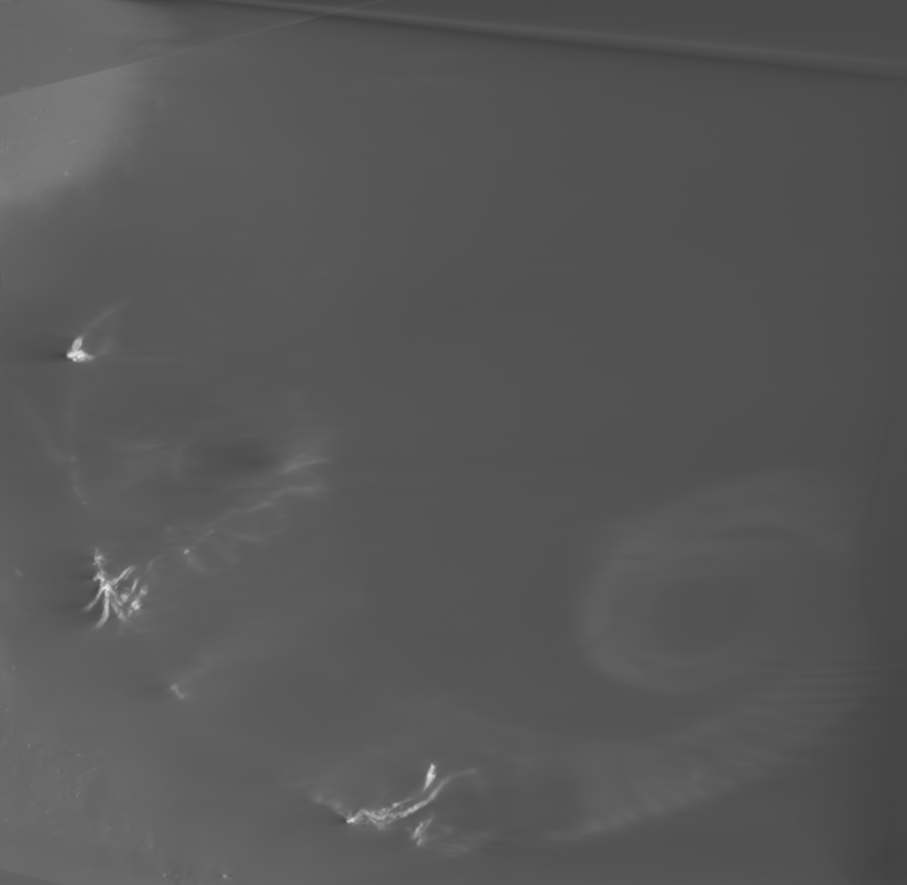

Supplement: Supplemental Information 2 — The used imagestack in the x-z-plane (bmp format) as it was obtained and used in SPIERS. [file peerj-05-3526-s002.zip › front_x-z-plane_072.bmp]

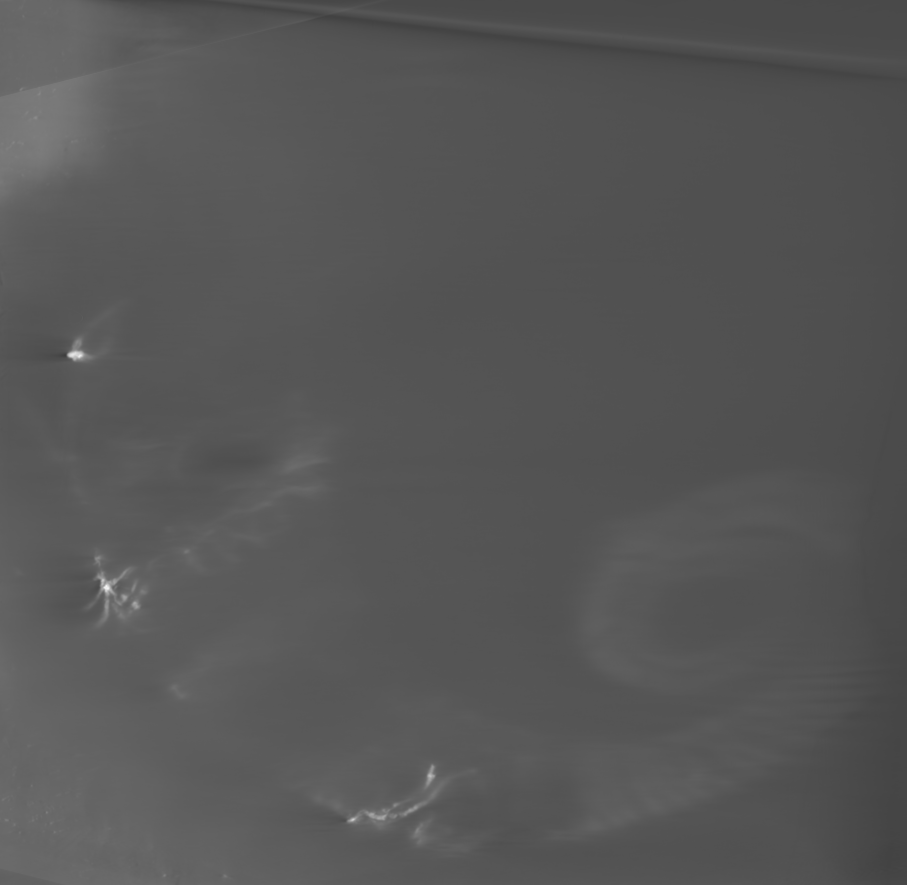

Supplement: Supplemental Information 2 — The used imagestack in the x-z-plane (bmp format) as it was obtained and used in SPIERS. [file peerj-05-3526-s002.zip › front_x-z-plane_073.bmp]

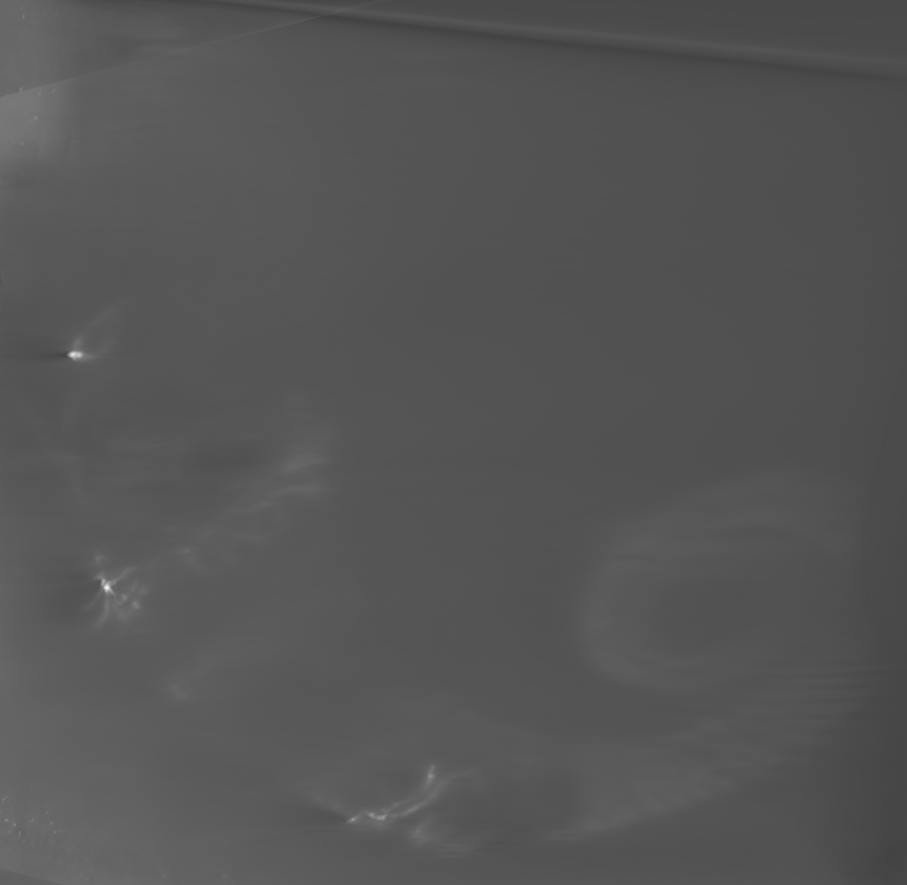

Supplement: Supplemental Information 2 — The used imagestack in the x-z-plane (bmp format) as it was obtained and used in SPIERS. [file peerj-05-3526-s002.zip › front_x-z-plane_074.bmp]

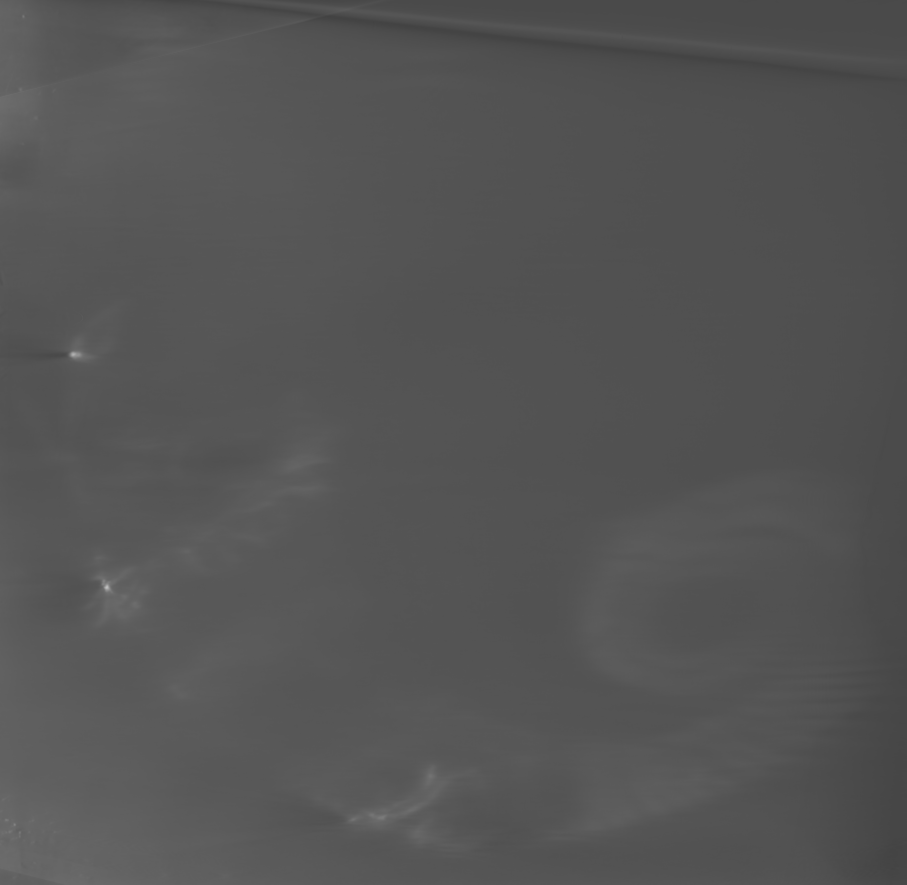

Supplement: Supplemental Information 2 — The used imagestack in the x-z-plane (bmp format) as it was obtained and used in SPIERS. [file peerj-05-3526-s002.zip › front_x-z-plane_075.bmp]

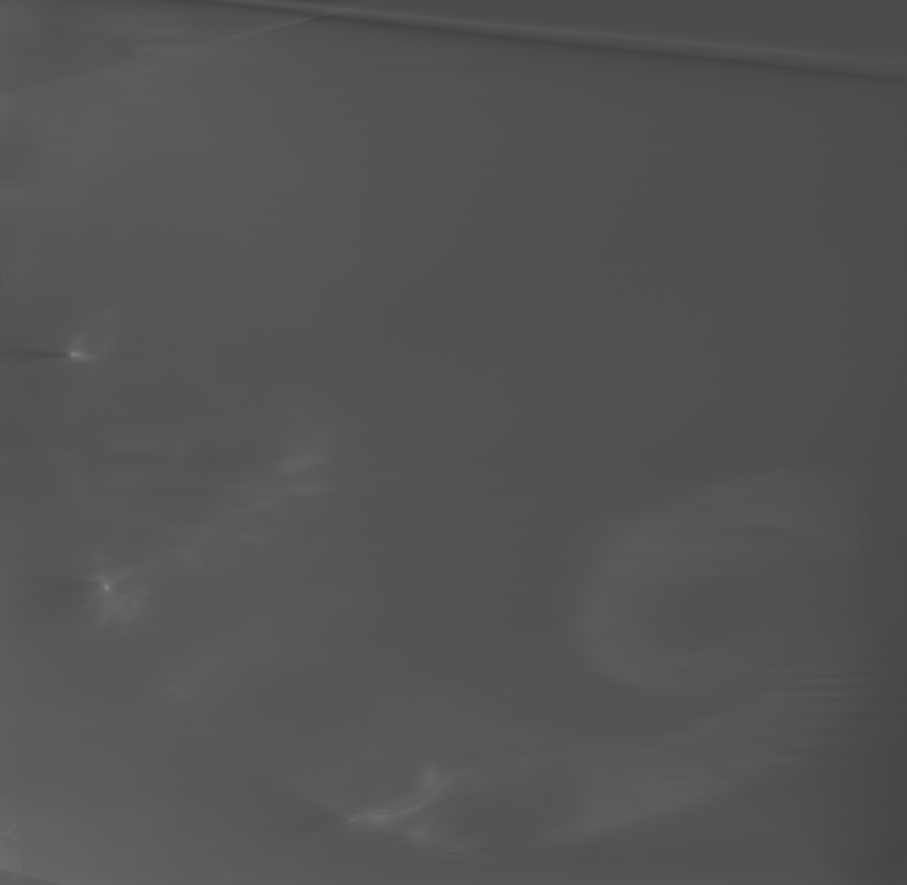

Supplement: Supplemental Information 2 — The used imagestack in the x-z-plane (bmp format) as it was obtained and used in SPIERS. [file peerj-05-3526-s002.zip › front_x-z-plane_076.bmp]

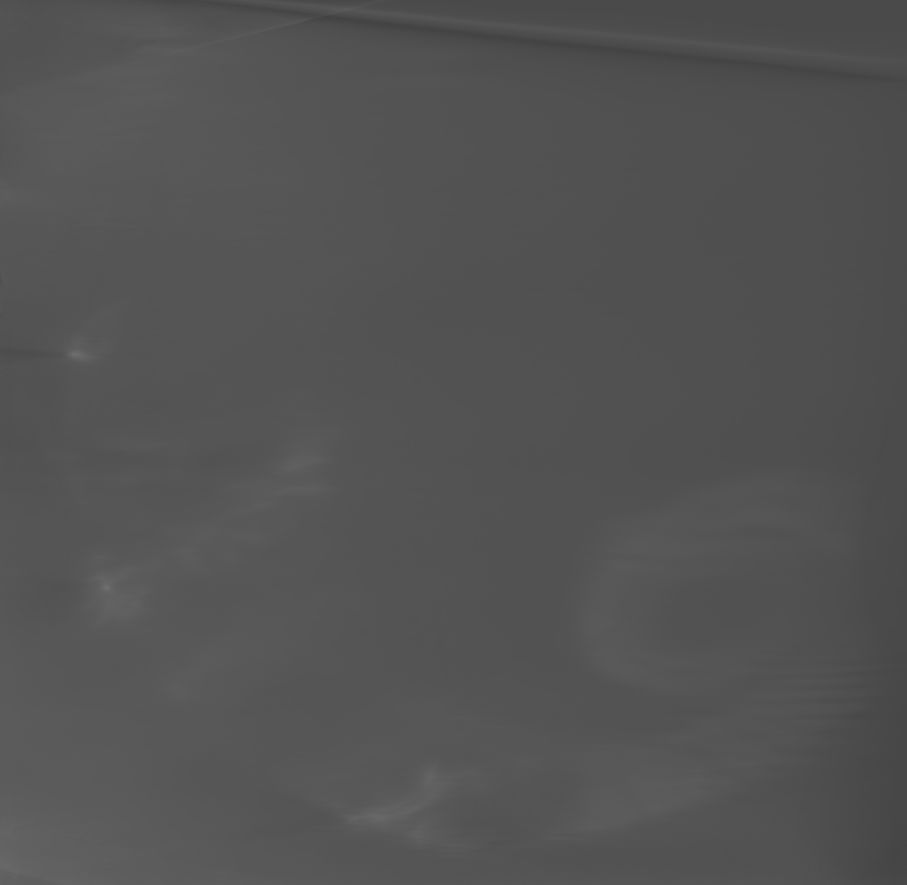

Supplement: Supplemental Information 2 — The used imagestack in the x-z-plane (bmp format) as it was obtained and used in SPIERS. [file peerj-05-3526-s002.zip › front_x-z-plane_077.bmp]

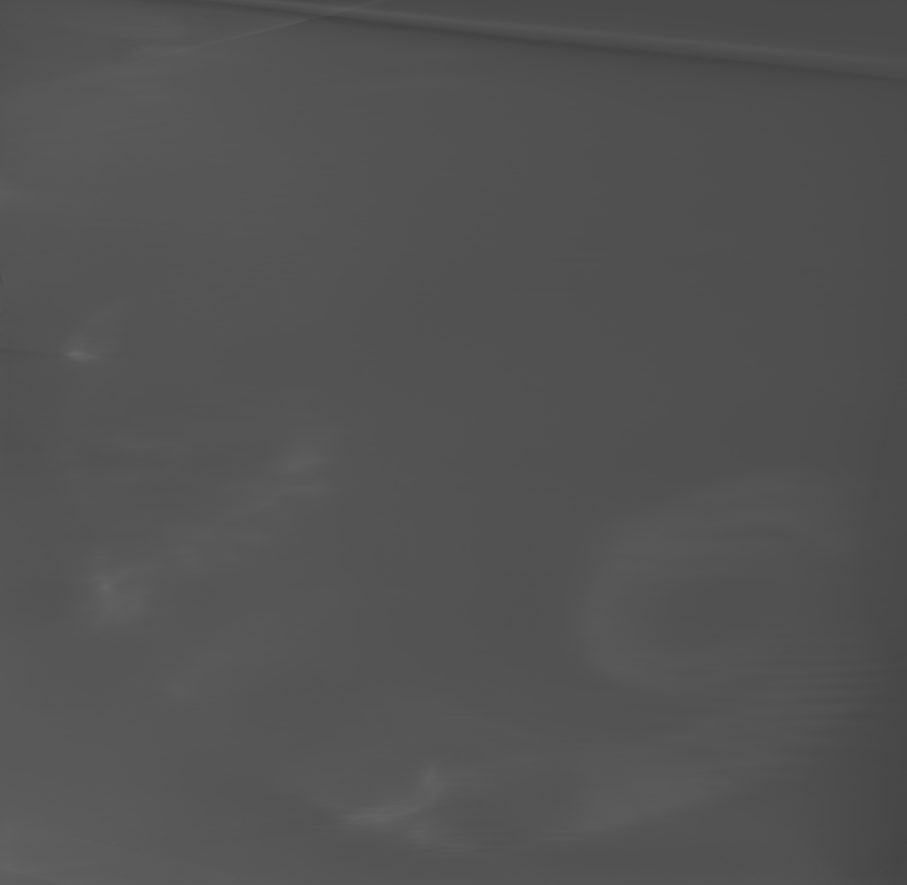

Supplement: Supplemental Information 2 — The used imagestack in the x-z-plane (bmp format) as it was obtained and used in SPIERS. [file peerj-05-3526-s002.zip › front_x-z-plane_078.bmp]

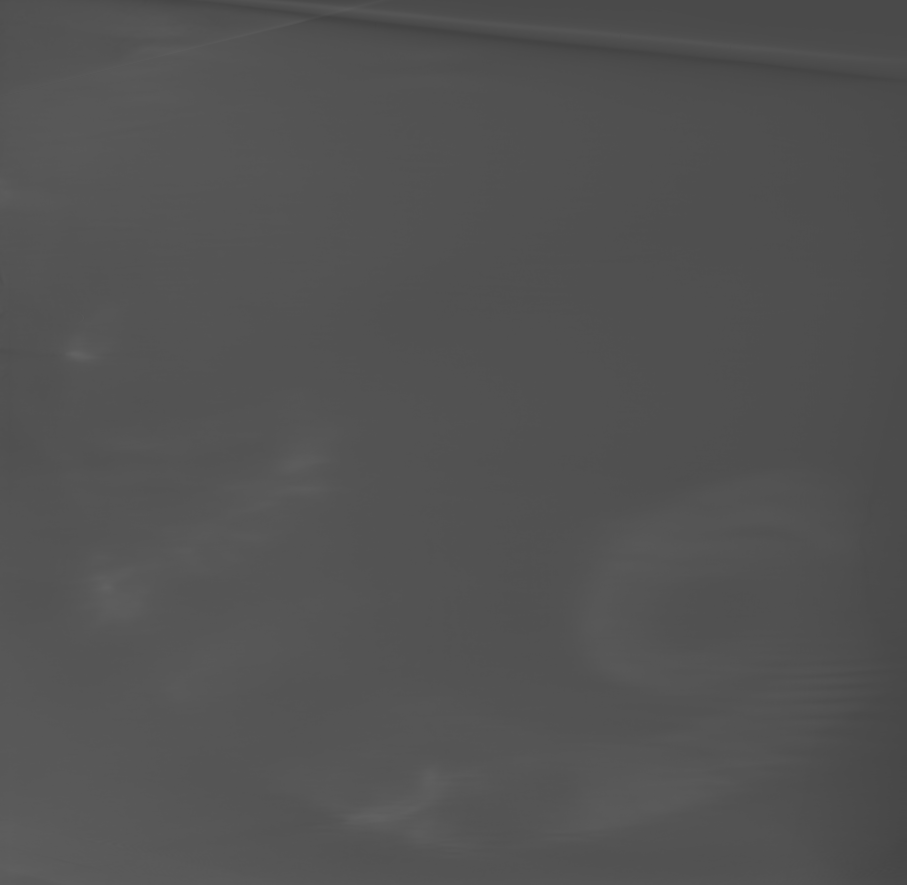

Supplement: Supplemental Information 2 — The used imagestack in the x-z-plane (bmp format) as it was obtained and used in SPIERS. [file peerj-05-3526-s002.zip › front_x-z-plane_079.bmp]

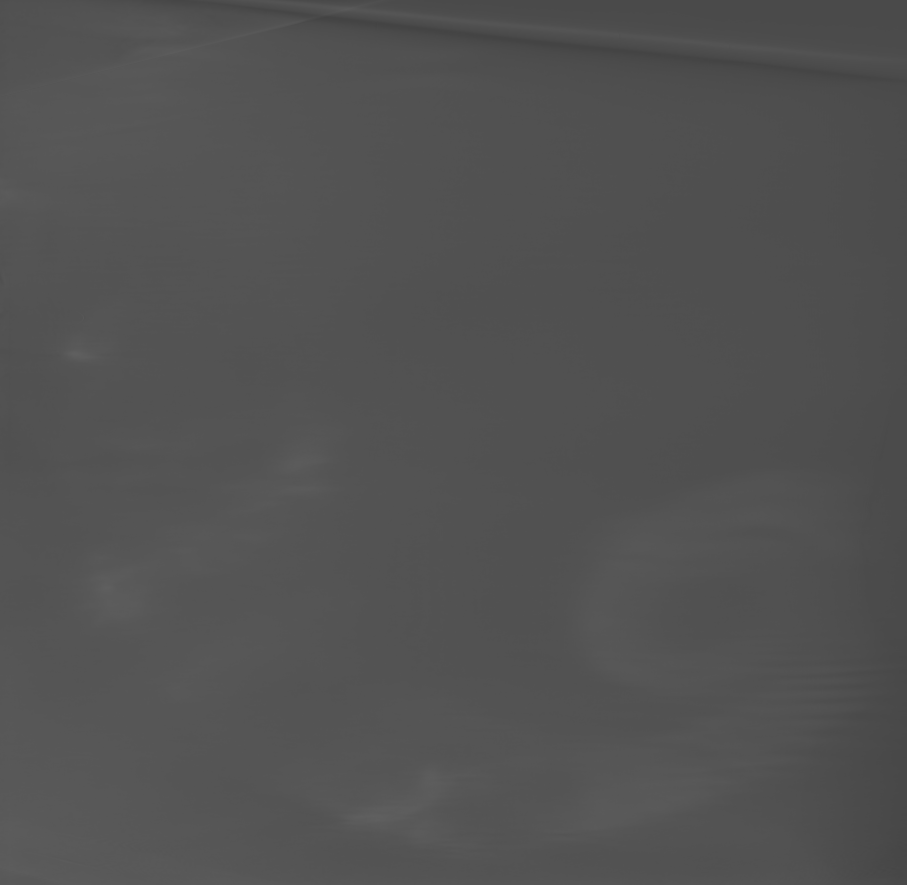

Supplement: Supplemental Information 2 — The used imagestack in the x-z-plane (bmp format) as it was obtained and used in SPIERS. [file peerj-05-3526-s002.zip › front_x-z-plane_080.bmp]

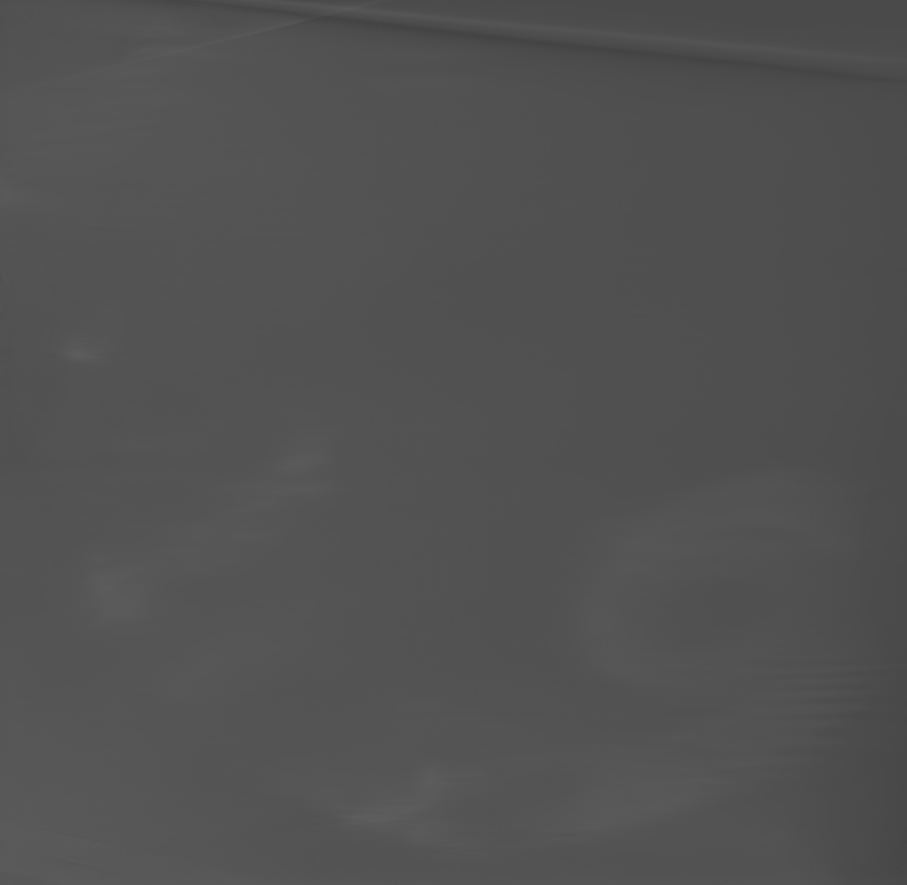

Supplement: Supplemental Information 2 — The used imagestack in the x-z-plane (bmp format) as it was obtained and used in SPIERS. [file peerj-05-3526-s002.zip › front_x-z-plane_081.bmp]

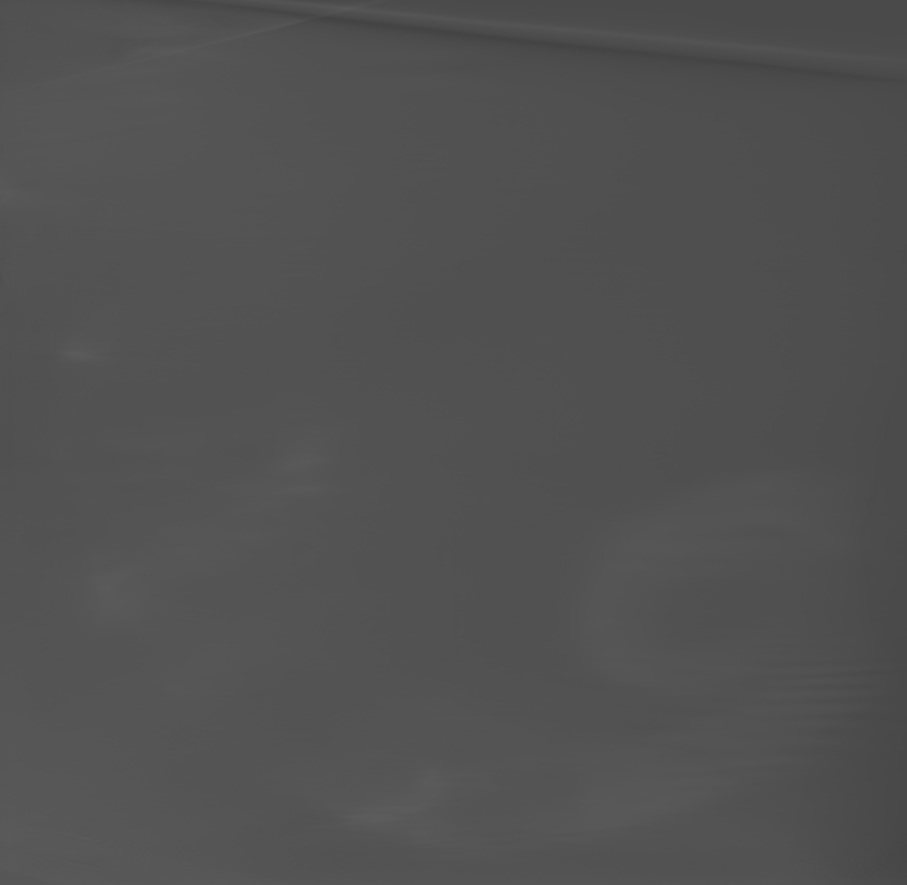

Supplement: Supplemental Information 2 — The used imagestack in the x-z-plane (bmp format) as it was obtained and used in SPIERS. [file peerj-05-3526-s002.zip › front_x-z-plane_082.bmp]

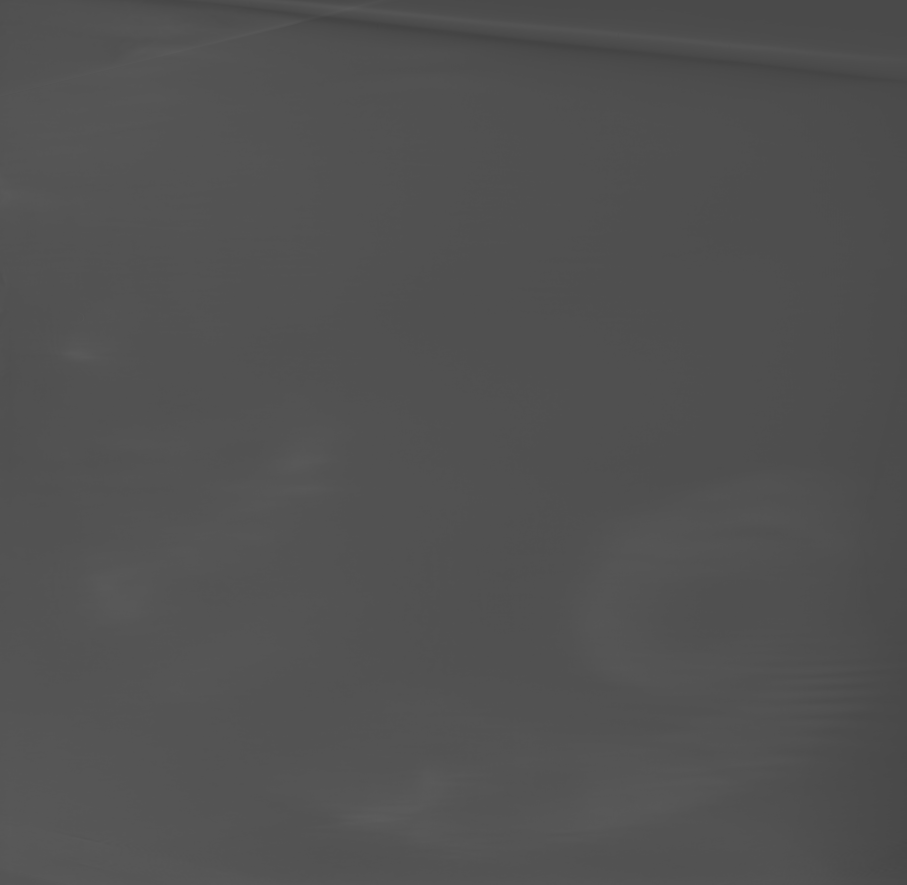

Supplement: Supplemental Information 2 — The used imagestack in the x-z-plane (bmp format) as it was obtained and used in SPIERS. [file peerj-05-3526-s002.zip › front_x-z-plane_083.bmp]

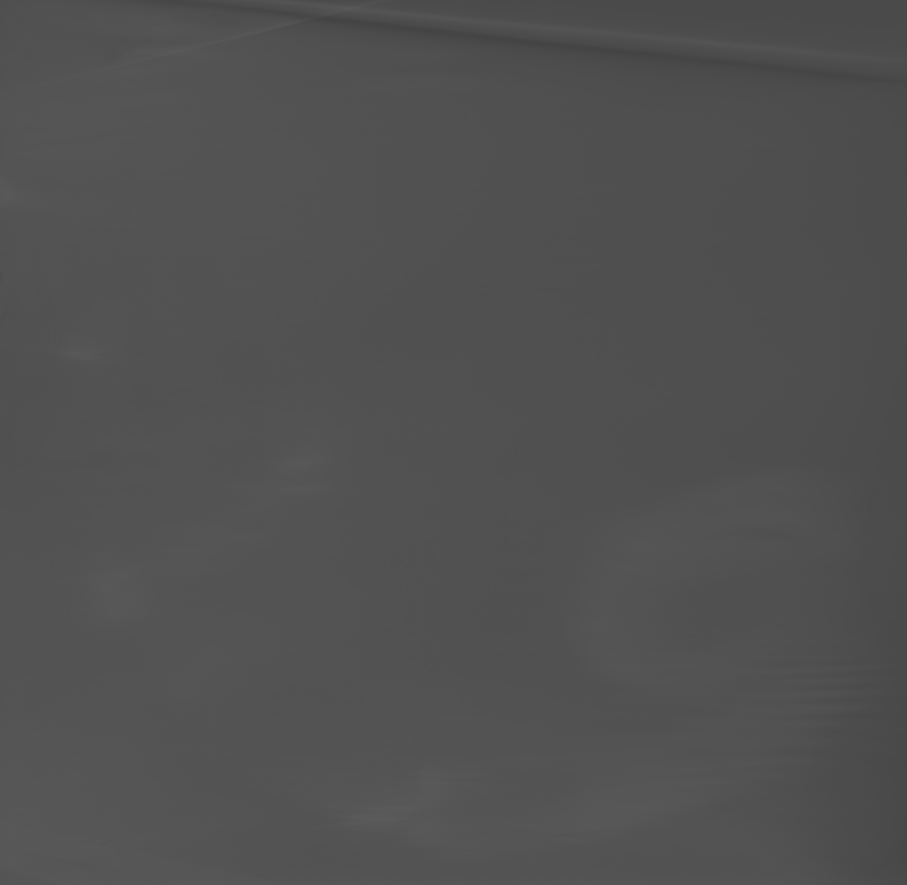

Supplement: Supplemental Information 2 — The used imagestack in the x-z-plane (bmp format) as it was obtained and used in SPIERS. [file peerj-05-3526-s002.zip › front_x-z-plane_084.bmp]

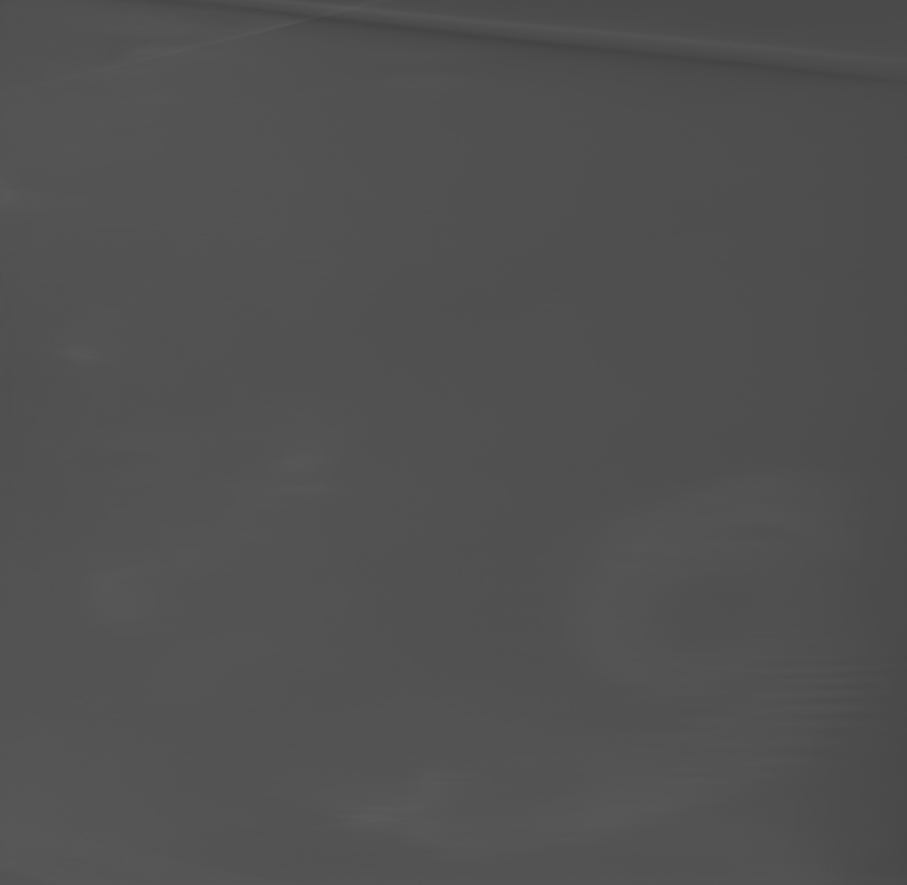

Supplement: Supplemental Information 2 — The used imagestack in the x-z-plane (bmp format) as it was obtained and used in SPIERS. [file peerj-05-3526-s002.zip › front_x-z-plane_085.bmp]

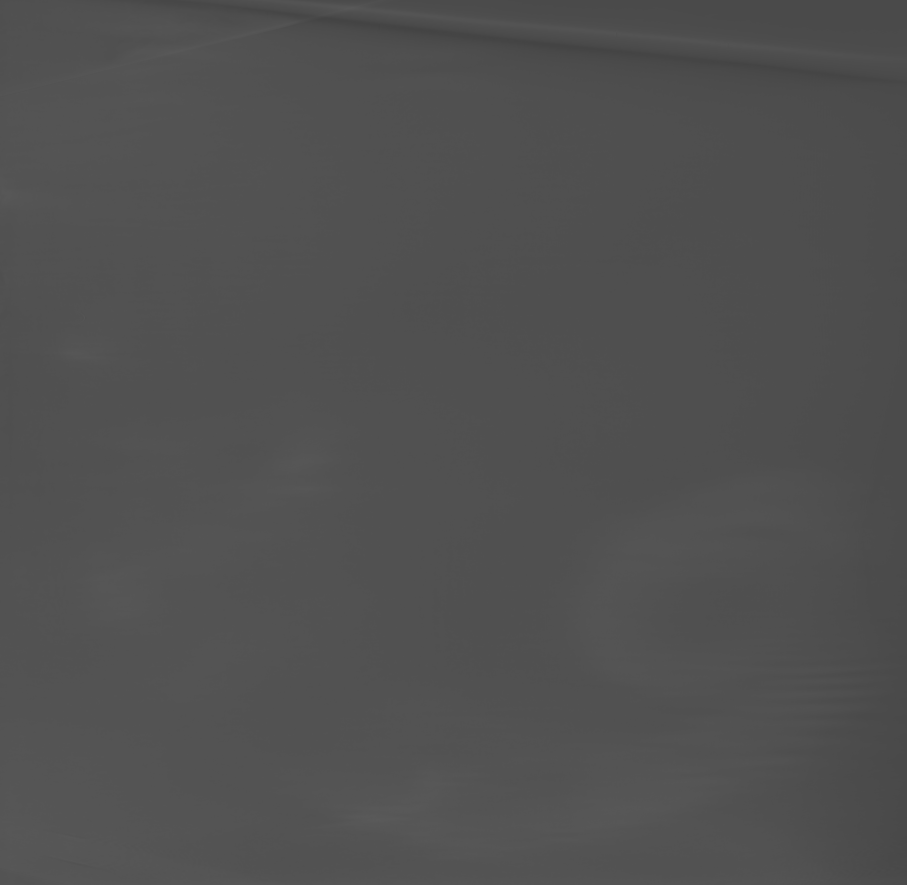

Supplement: Supplemental Information 2 — The used imagestack in the x-z-plane (bmp format) as it was obtained and used in SPIERS. [file peerj-05-3526-s002.zip › front_x-z-plane_086.bmp]

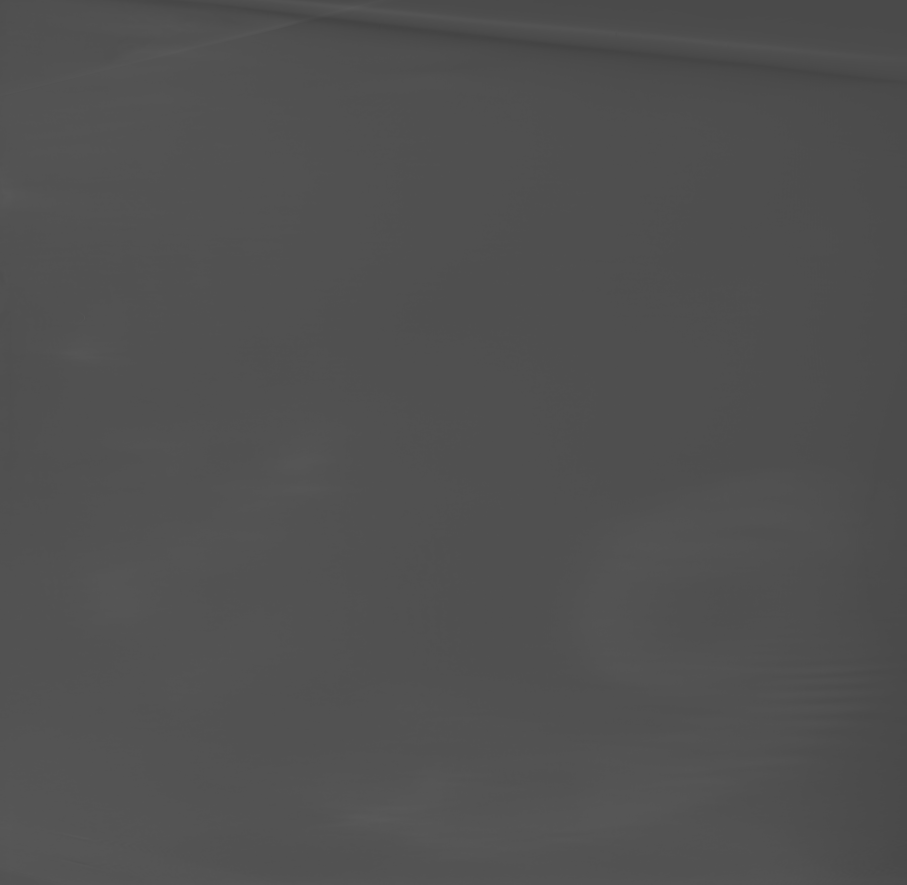

Supplement: Supplemental Information 2 — The used imagestack in the x-z-plane (bmp format) as it was obtained and used in SPIERS. [file peerj-05-3526-s002.zip › front_x-z-plane_087.bmp]

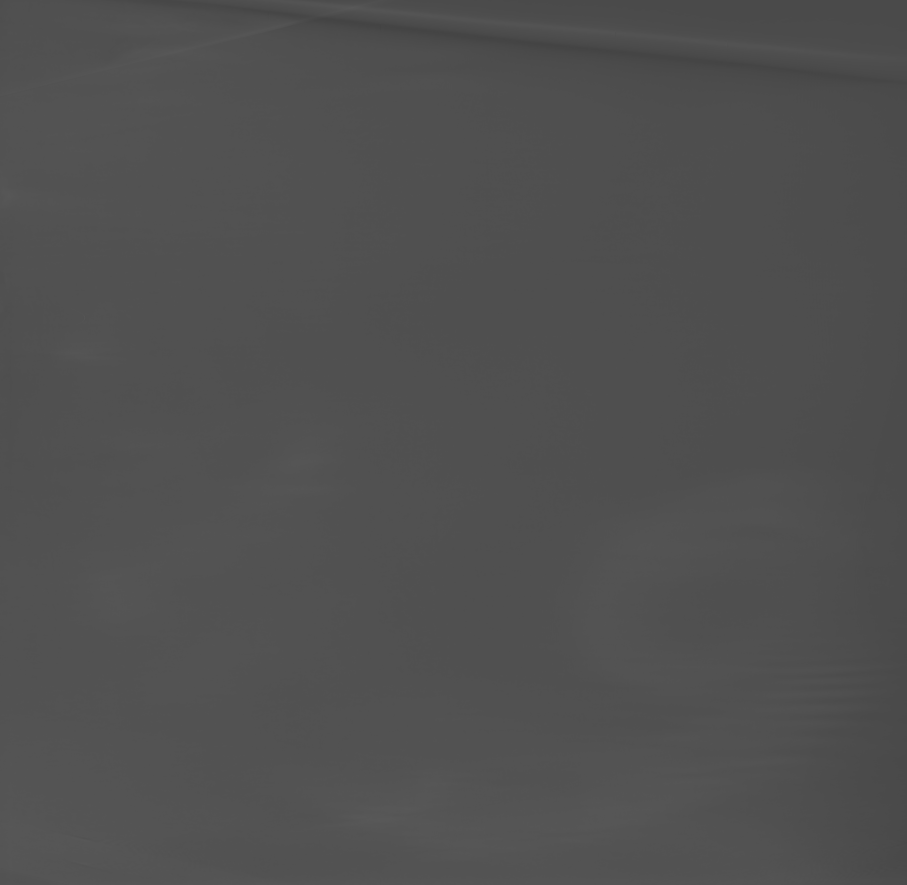

Supplement: Supplemental Information 2 — The used imagestack in the x-z-plane (bmp format) as it was obtained and used in SPIERS. [file peerj-05-3526-s002.zip › front_x-z-plane_088.bmp]

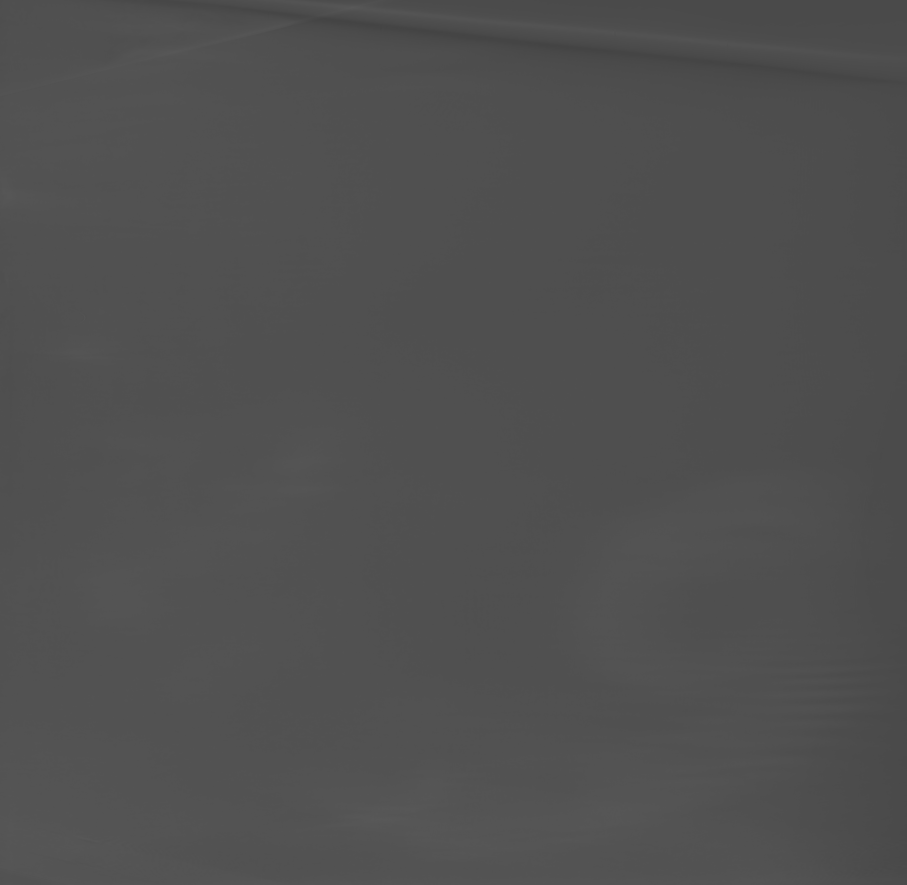

Supplement: Supplemental Information 2 — The used imagestack in the x-z-plane (bmp format) as it was obtained and used in SPIERS. [file peerj-05-3526-s002.zip › front_x-z-plane_089.bmp]

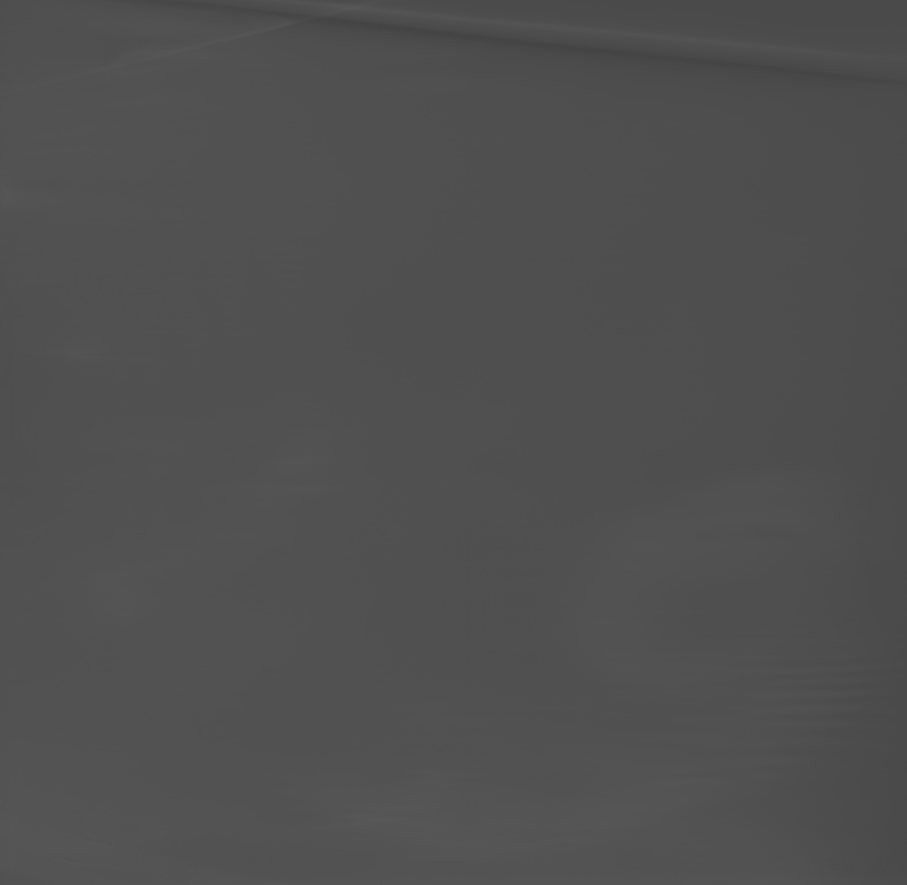

Supplement: Supplemental Information 2 — The used imagestack in the x-z-plane (bmp format) as it was obtained and used in SPIERS. [file peerj-05-3526-s002.zip › front_x-z-plane_090.bmp]

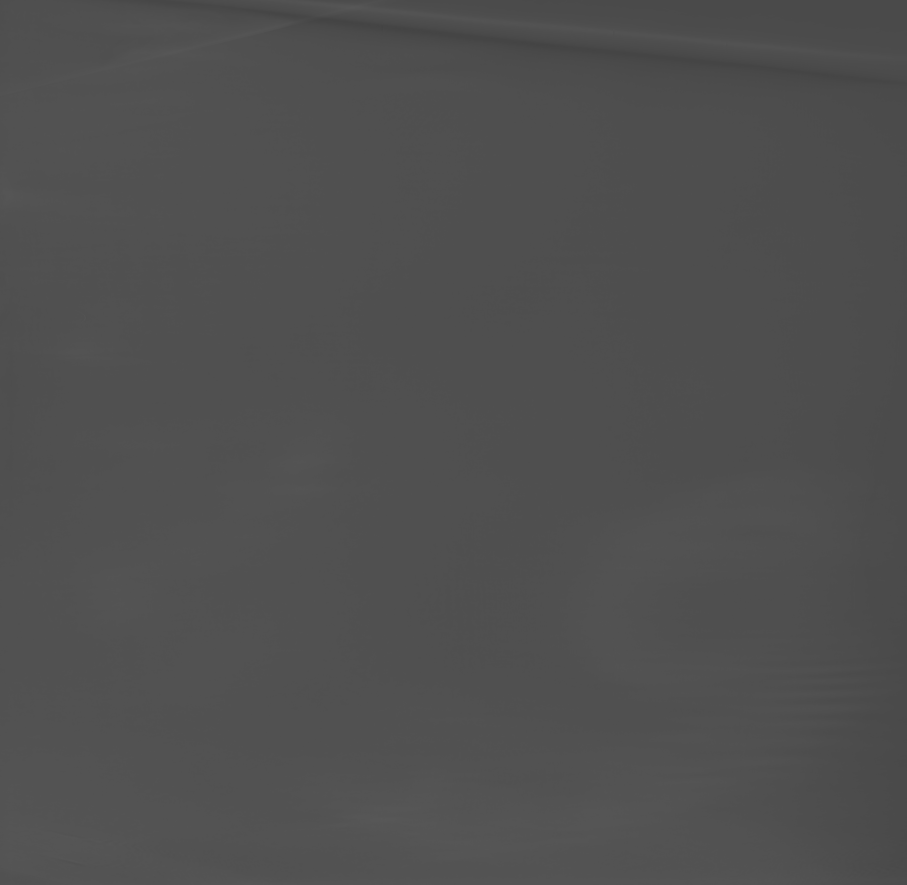

Supplement: Supplemental Information 2 — The used imagestack in the x-z-plane (bmp format) as it was obtained and used in SPIERS. [file peerj-05-3526-s002.zip › front_x-z-plane_091.bmp]

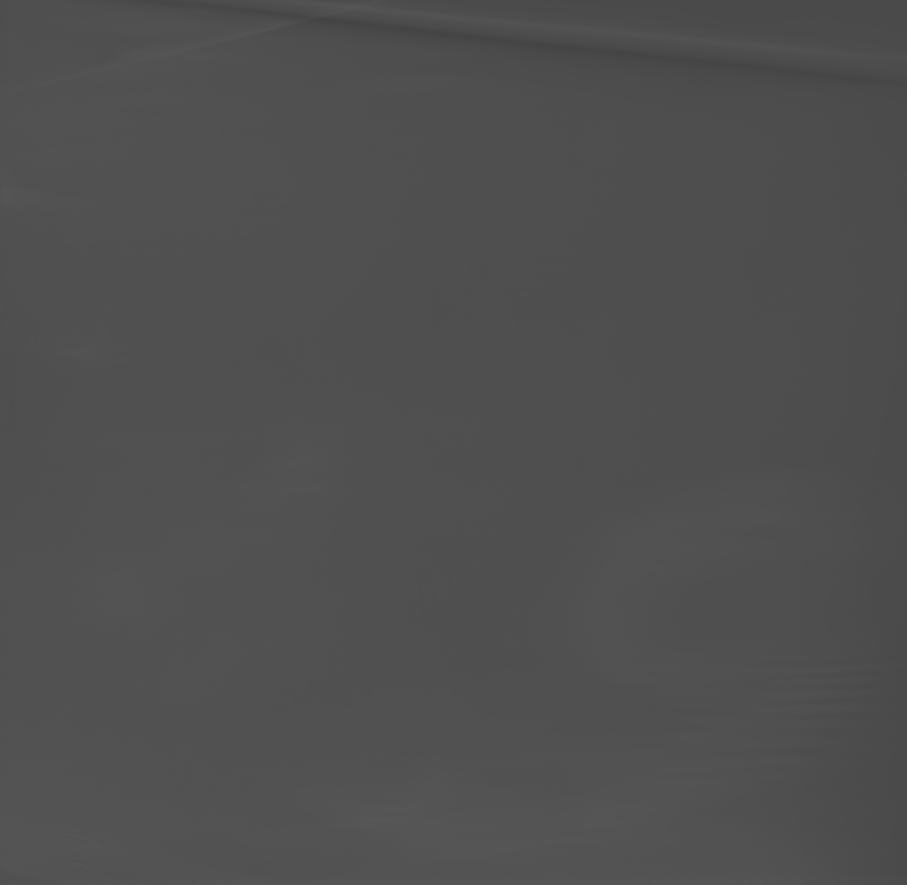

Supplement: Supplemental Information 2 — The used imagestack in the x-z-plane (bmp format) as it was obtained and used in SPIERS. [file peerj-05-3526-s002.zip › front_x-z-plane_092.bmp]

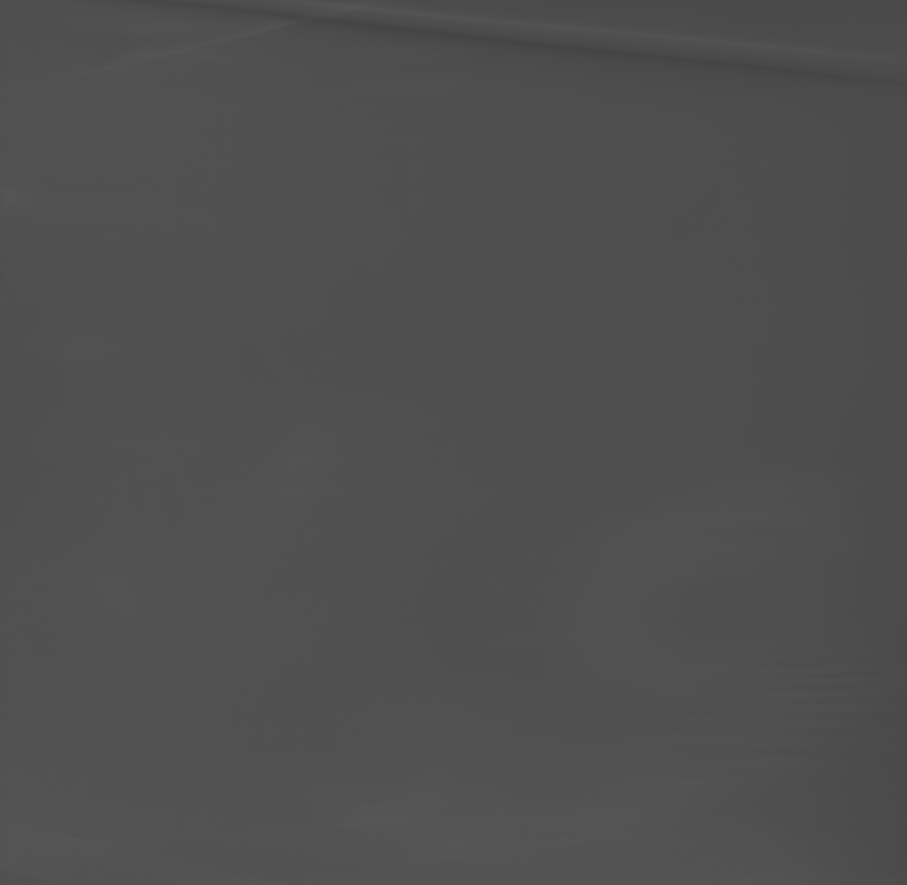

Supplement: Supplemental Information 2 — The used imagestack in the x-z-plane (bmp format) as it was obtained and used in SPIERS. [file peerj-05-3526-s002.zip › front_x-z-plane_093.bmp]

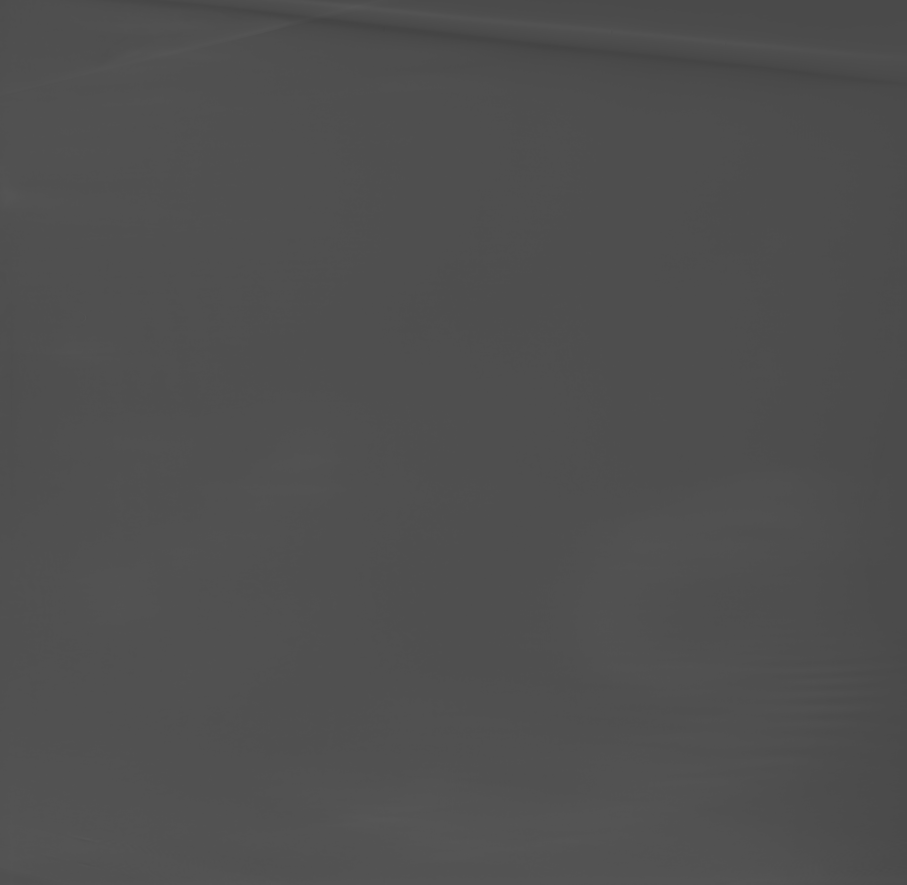

Supplement: Supplemental Information 2 — The used imagestack in the x-z-plane (bmp format) as it was obtained and used in SPIERS. [file peerj-05-3526-s002.zip › front_x-z-plane_094.bmp]

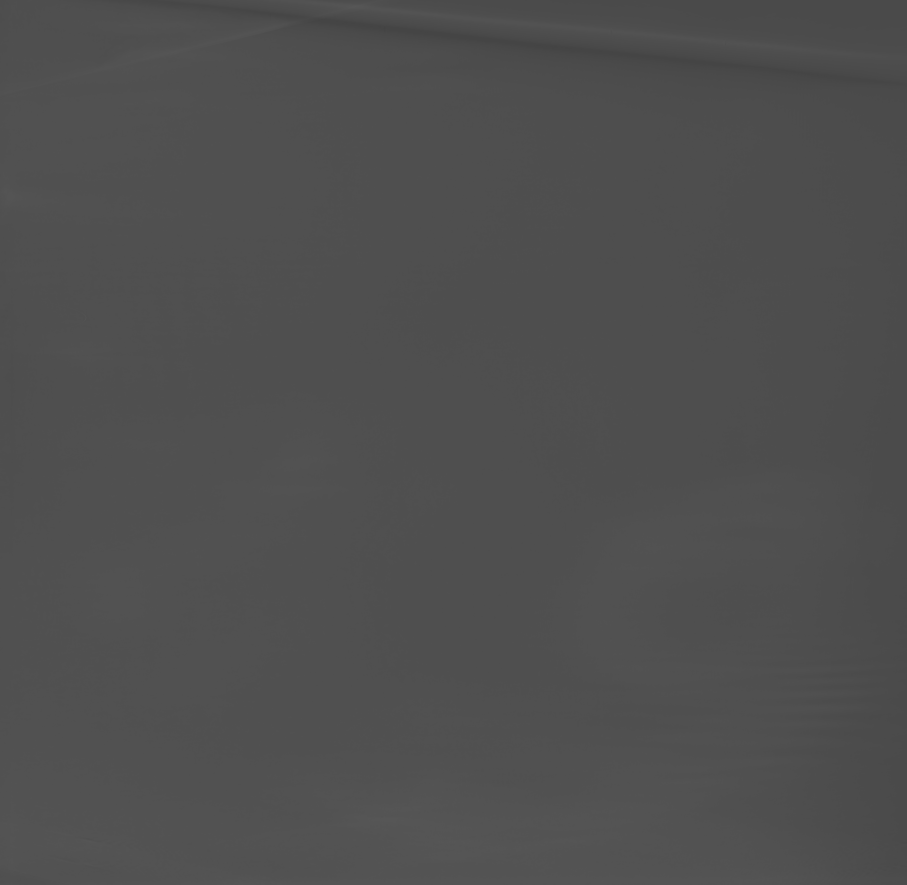

Supplement: Supplemental Information 2 — The used imagestack in the x-z-plane (bmp format) as it was obtained and used in SPIERS. [file peerj-05-3526-s002.zip › front_x-z-plane_095.bmp]

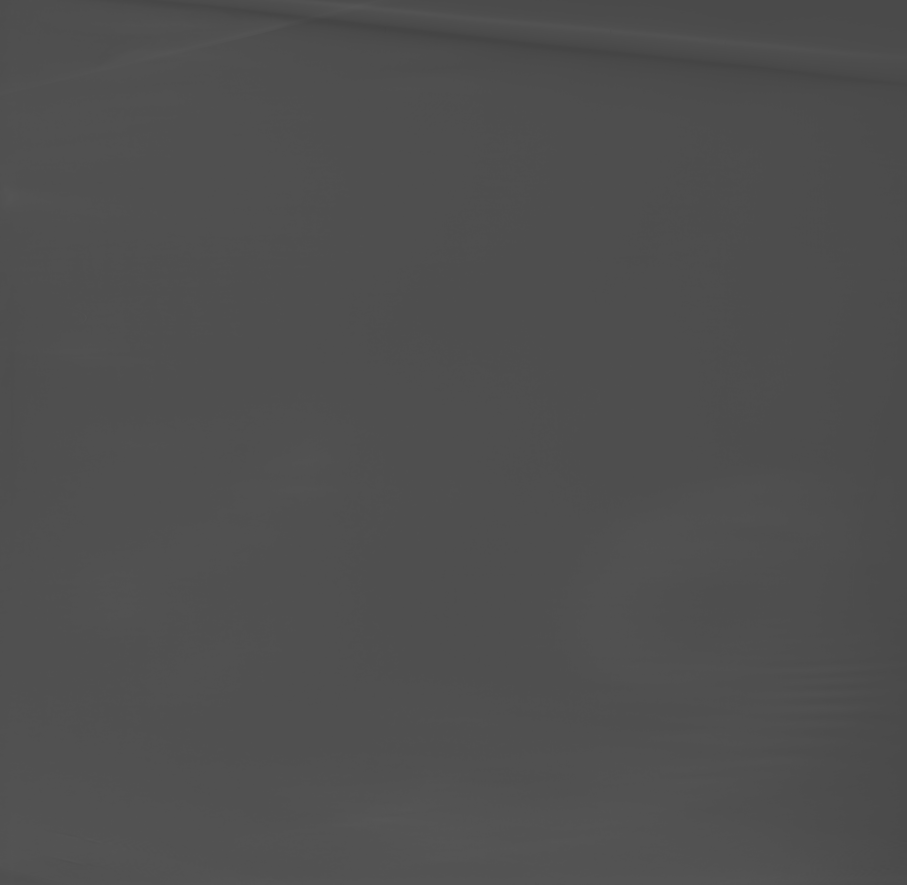

Supplement: Supplemental Information 2 — The used imagestack in the x-z-plane (bmp format) as it was obtained and used in SPIERS. [file peerj-05-3526-s002.zip › front_x-z-plane_096.bmp]

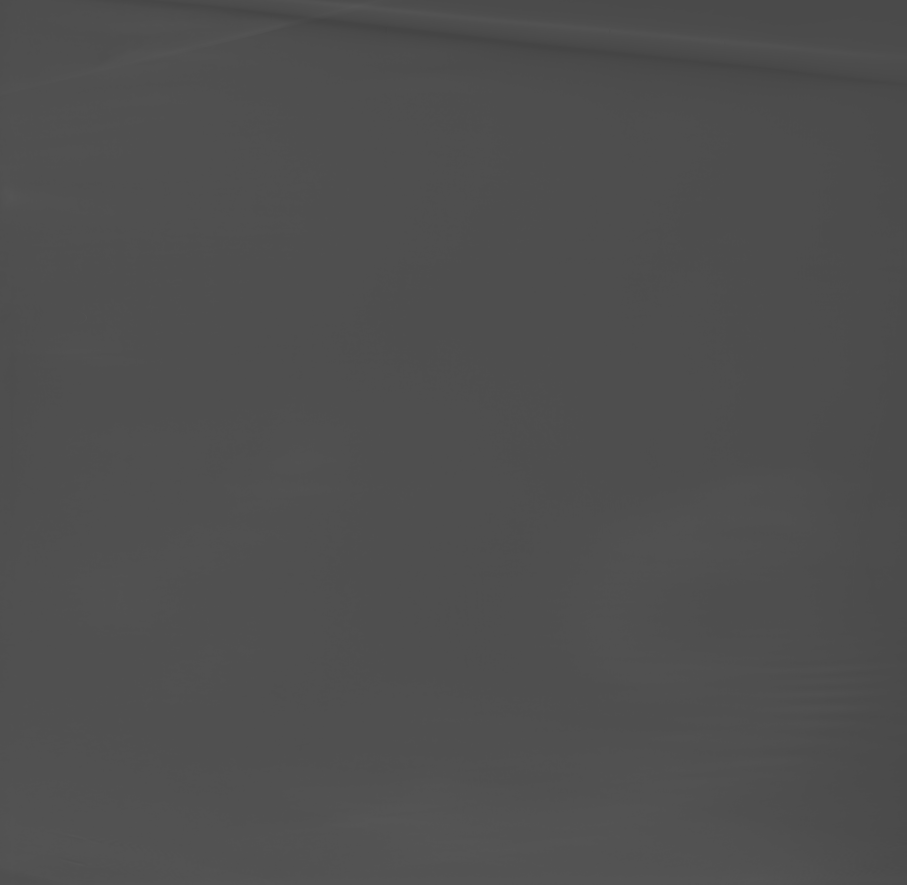

Supplement: Supplemental Information 2 — The used imagestack in the x-z-plane (bmp format) as it was obtained and used in SPIERS. [file peerj-05-3526-s002.zip › front_x-z-plane_097.bmp]

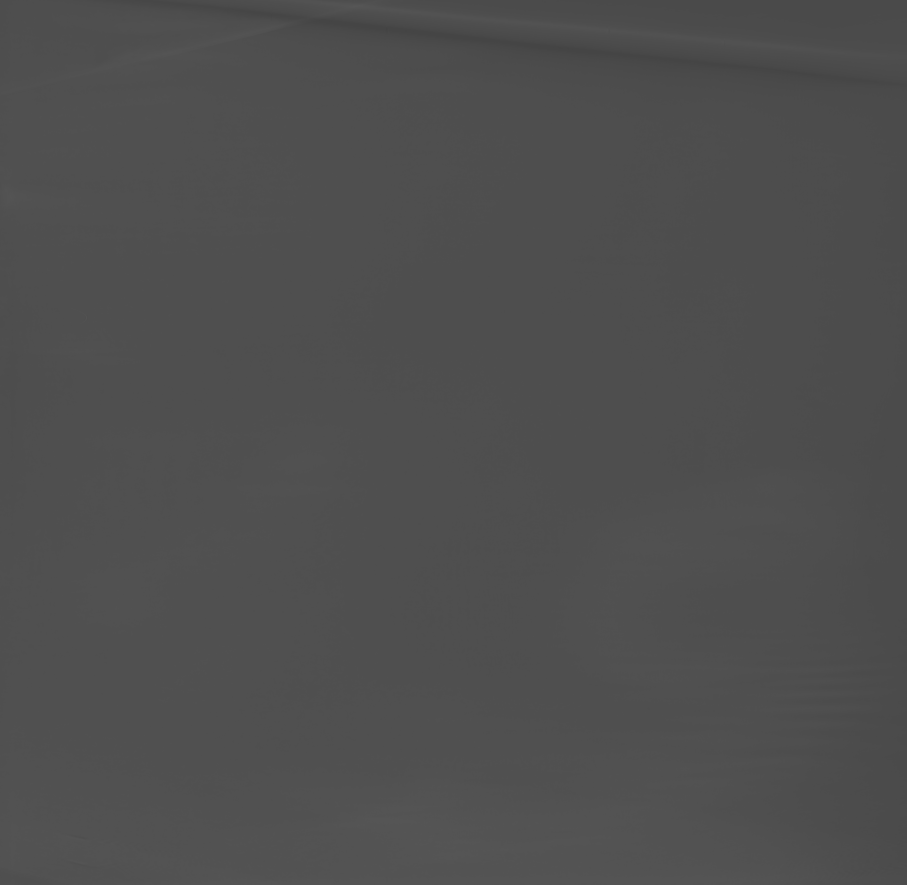

Supplement: Supplemental Information 2 — The used imagestack in the x-z-plane (bmp format) as it was obtained and used in SPIERS. [file peerj-05-3526-s002.zip › front_x-z-plane_098.bmp]

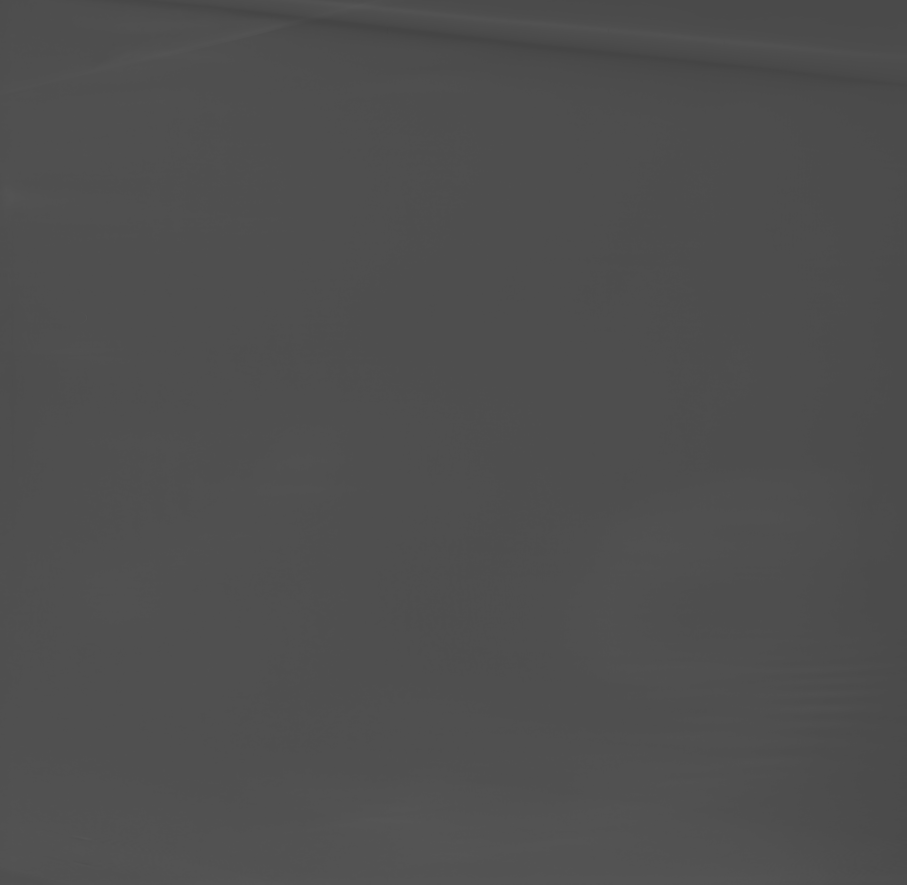

Supplement: Supplemental Information 2 — The used imagestack in the x-z-plane (bmp format) as it was obtained and used in SPIERS. [file peerj-05-3526-s002.zip › front_x-z-plane_099.bmp]
