# Supplementary material for: 3D-Analysis of a non-planispiral ammonoid from the Hunsrück Slate: natural or pathological variation?
Source: PeerJ. 2017 Jun 30;5:e3526. doi: 10.7717/peerj.3526 (PMC5494166; doi:10.7717/peerj.3526)
Supplement: Supplemental Information 3 — Text file with description of scan settings, voxel size, and specimen information (repository and accession number). [file peerj-05-3526-s003.rtf]

S C A N S H E E T_________________

3D-Scan - Scan-Date: 14.03.2011
Voxelsize: 118.1114µm 
___________________________________________________________________
Scientificname: Ivoites opitzi
Commonname: Ammonoid
Bodypart: 
Stratigraphy / Location: Middle Kaub Formation / Schieleberg-quarry near Herrstein
Constitution : Fossil
___________________________________________________________________
Taxa:
Group: Ammonoidea
Family: Mimosphinctidae
Genus: Ivoites
Species: opitzi
___________________________________________________________________
Collection:
Inventory-ID: KGM 1983/147

Karl Geib Museum Bad Kreuznach

Notes:
___________________________________________________________________
Scan-Parameter:
Voltage: 150 kV	Current: 160 µA	Timing: 400 ms
___________________________________________________________________
Scan-Device:
V|tome|x s Phoenix x-ray
Steinmann-Institut
___________________________________________________________________
Scientist: Poschmann, Markus
Operator: Bergmann, Alexandra
___________________________________________________________________
Additional Informations:
Encrusted ammonoid
___________________________________________________________________
